# Benmelstobart, anlotinib and chemotherapy in extensive-stage small-cell lung cancer: a randomized phase 3 trial

**DOI:** 10.1038/s41591-024-03132-1

**Published:** 2024-07-11

**Authors:** Ying Cheng, Jianhua Chen, Wei Zhang, Chao Xie, Qun Hu, Ningning Zhou, Chun Huang, Shihong Wei, Hong Sun, Xingya Li, Yan Yu, Jinhuo Lai, Huaping Yang, Haohui Fang, Hualin Chen, Peng Zhang, Kangsheng Gu, Qiming Wang, Jianhua Shi, Tienan Yi, Xingxiang Xu, Xianwei Ye, Daqing Wang, Conghua Xie, Chunling Liu, Yulong Zheng, Daren Lin, Wu Zhuang, Ping Lu, Guohua Yu, Jinzhang Li, Yuhai Gu, Baolan Li, Rong Wu, Ou Jiang, Zaiyi Wang, Guowu Wu, Haifeng Lin, Diansheng Zhong, Yanhua Xu, Yongqian Shu, Di Wu, Xingwu Chen, Jie Wang, Minghui Wang, Runxiang Yang

**Affiliations:** 1 Jilin Cancer Hospital, Changchun, China; 2 Hunan Cancer Hospital, Changsha, China; 3 The First Affiliated Hospital of Nanchang University, Nanchang, China; 4 Shandong Cancer Hospital and Institute, Shandong University, Jinan, China; 5 The Affiliated Hospital of Inner Mongolia University, Hohhot, China; 6 Sun Yat-sen University Cancer Center, Guangzhou, China; 7 Tianjin Medical University Cancer Institute and Hospital, Tianjin, China; 8 Gansu Provincial Cancer Hospital, Lanzhou, China; 9 The First Affiliated Hospital of Xi’an Jiaotong University, Xi’an, China; 10 The First Affiliated Hospital of Zhengzhou University, Zhengzhou, China; 11 Harbin Medical University Cancer Hospital, Harbin, China; 12 Fujian Medical University Union Hospital, Fuzhou, China; 13 Xiangya Hospital Central South University, Changsha, China; 14 Anhui Chest Hospital, Hefei, China; 15 Affiliated Hospital of Guangdong Medical University, Zhanjiang, China; 16 Shanghai Pulmonary Hospital, Shanghai, China; 17 The First Affiliated Hospital of Anhui Medical University, Hefei, China; 18 Henan Cancer Hospital, Affiliated Cancer Hospital of Zhengzhou University, Zhengzhou, China; 19 Linyi Cancer Hospital, Linyi, China; 20 Xiangyang Central Hospital, Xiangyang, China; 21 Northern Jiangsu People’s Hospital, Yangzhou, China; 22 Guizhou Provincial People’s Hospital, Guiyang, China; 23 Hengshui People’s Hospital, Hengshui, China; 24 Zhongnan Hospital of Wuhan University, Wuhan, China; 25 Cancer Hospital Affiliated to Xinjiang Medical University, Urumqi, China; 26 The First Affiliated Hospital, Zhejiang University School of Medicine, Hangzhou, China; 27 Jiangmen Central Hospital, Jiangmen, China; 28 Fujian Cancer Hospital, Fuzhou, China; 29 The First Affiliated Hospital of Xinxiang Medical College, Xinxiang, China; 30 Weifang People’s Hospital, Weifang, China; 31 Qinghai University Affiliated Hospital, Xining, China; 32 Qinghai Provincial People’s Hospital, Xining, China; 33 Beijing Chest Hospital, Capital Medical University, Beijing, China; 34 Shengjing Hospital of China Medical University, Shenyang, China; 35 The Second People’s Hospital of Neijiang, Neijiang, China; 36 The First Affiliated Hospital of Xinjiang Medical University, Urumqi, China; 37 Meizhou People’s Hospital, Meizhou, China; 38 The Second Affiliated Hospital of Hainan Medical University, Haikou, China; 39 Tianjin Medical University General Hospital, Tianjin, China; 40 Jingzhou Central Hospital, Jingzhou, China; 41 Jiangsu Province Hospital, Nanjing, China; 42 Shenzhen People’s Hospital, Shenzhen, China; 43 The First Affiliated Hospital of Wannan Medical College, Wuhu, China; 44 Cancer Hospital, Chinese Academy of Medical Sciences, Beijing, China; 45 Sun Yat-sen Memorial Hospital, Sun Yat-sen University, Guangzhou, China; 46 Yunnan Cancer Hospital, Kunming, China

**Keywords:** Lung cancer, Randomized controlled trials

## Abstract

Immunochemotherapy is the first-line standard for extensive-stage small-cell lung cancer (ES-SCLC). Combining the regimen with anti-angiogenesis may improve efficacy. ETER701 was a multicenter, double-blind, randomized, placebo-controlled phase 3 trial that investigated the efficacy and safety of benmelstobart (a novel programmed death-ligand 1 (PD-L1) inhibitor) with anlotinib (a multi-target anti-angiogenic small molecule) and standard chemotherapy in treatment-naive ES-SCLC. The ETER701 trial assessed two primary endpoints: Independent Review Committee-assessed progression-free survival per RECIST 1.1 and overall survival (OS). Here the prespecified final progression-free survival and interim OS analysis is reported. Patients randomly received benmelstobart and anlotinib plus etoposide/carboplatin (EC; *n* = 246), placebo and anlotinib plus EC (*n* = 245) or double placebo plus EC (‘EC alone’; *n* = 247), followed by matching maintenance therapy. Compared with EC alone, median OS was prolonged with benmelstobart and anlotinib plus EC (19.3 versus 11.9 months; hazard ratio 0.61; *P* = 0.0002), while improvement of OS was not statistically significant with anlotinib plus EC (13.3 versus 11.9 months; hazard ratio 0.86; *P* = 0.1723). The incidence of grade 3 or higher treatment-related adverse events was 93.1%, 94.3% and 87.0% in the benmelstobart and anlotinib plus EC, anlotinib plus EC, and EC alone groups, respectively. This study of immunochemotherapy plus multi-target anti-angiogenesis as first-line treatment achieved a median OS greater than recorded in prior randomized studies in patients with ES-SCLC. The safety profile was assessed as tolerable and manageable. Our findings suggest that the addition of anti-angiogenesis therapy to immunochemotherapy may represent an efficacious and safe approach to the management of ES-SCLC. ClinicalTrials.gov identifier: NCT04234607.

## Abstract (web summary)

In this triple-arm, placebo-controlled phase 3 trial, first-line treatment of patients with extensive-stage small-cell lung cancer with the anti-PD-L1 benmelstobart, tyrosine kinase inhibitor anlotinib and chemotherapy (CT) showed improved survival outcomes compared with anlotinib and CT or CT alone.

## Main

Small-cell lung cancer (SCLC) represents approximately 15% of lung cancer cases and has a poor prognosis; about two-thirds of patients present at an extensive-stage (ES) disease state, and the 5-year overall survival (OS) rate is limited to only 1–5% (refs. ^1,2^). The addition of an immune checkpoint inhibitor (ICI) to platinum-based chemotherapy has been associated with prolonged median survival^2^, and four randomized, controlled phase 3 studies (IMpower133, CASPIAN, CAPSTONE-1 and ASTRUM-005) have reported significant improvements in median OS on the order of 2–4 months^3–6^. The addition of a T cell immunoglobulin and ITIM domain inhibitor to immunochemotherapy has, so far, failed to demonstrate further improvement in survival^1^.

Anti-angiogenesis is known to augment the efficacy of ICIs in multiple cancer types^7^, and the normalization of blood vessels may promote immune cell infiltration and change the tumor microenvironment^8,9^. Anlotinib is a multitargeted anti-angiogenic agent with a potential synergistic effect with ICI therapy^10^, and anlotinib monotherapy has been approved for third-line treatment of ES-SCLC in China^11^. Benmelstobart (TQB2450) is a humanized anti-programmed death-ligand 1 (PD-L1) antibody that has demonstrated preclinical antitumor activity similar to other ICIs^12^. A phase 1b dose-escalation study that investigated the combination of anlotinib and benmelstobart demonstrated a favorable safety profile and, interestingly, reported that four out of six enrolled patients with ES-SCLC attained a partial response^13^.

This multicenter, double-blind, randomized, placebo-controlled phase 3 study compared three-drug regimens: benmelstobart and anlotinib plus etoposide/carboplatin (EC; *n* = 246), placebo/anlotinib plus EC (*n* = 245) or double placebo/EC chemotherapy alone (hereafter to referred to as ‘EC alone’; *n* = 247); the latter was the standard of care in China at the point of study protocol approval (August 2019). ETER701 (NCT04234607) was designed to address two questions: whether the addition of benmelstobart and anlotinib to chemotherapy showed synergistic benefit in the first-line treatment of ES-SCLC and whether the addition of anlotinib alone to chemotherapy provided clinical benefit. Because no programmed death 1 (PD-1)/PD-L1 inhibitors for ES-SCLC were available in China at the approval of the study protocol, EC chemotherapy was adopted as the control arm in ETER701.

## Results

### Patients and treatment

**Fig. 1:**
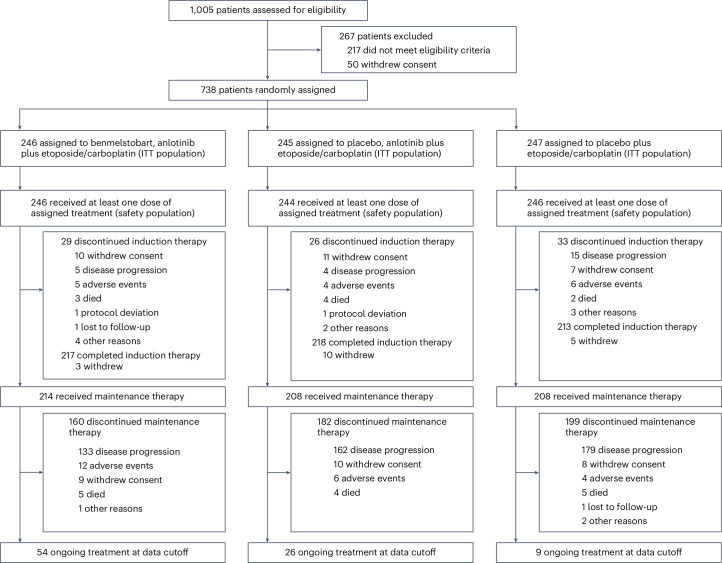
Patients disposition. The primary reason for treatment discontinuation is shown in each treatment group, mainly including disease progression or death.

From 18 March 2020 to 18 December 2021, 1,005 patients at 72 sites were assessed for eligibility. Overall, 738 were randomized to treatment (intention-to-treat (ITT) population); 246 were assigned to receive benmelstobart and anlotinib plus EC, 245 to receive anlotinib plus EC, and 247 to receive EC (Fig. 1). Baseline demographic and disease characteristics were well balanced across treatment groups (Table 1). Most patients had stage IV disease (660 of 738 (89.4%)), with a median age of 62 (range 30–75) years at diagnosis, and 73 patients (9.9%) had brain metastases at baseline.

**Table 1:** Baseline demographic and characteristics of patients in the ITT population

	Benmelstobart + anlotinib + EC group (*N* = 246)	Anlotinib + EC group (*N* = 245)	EC alone group (*N* = 247)
Age, years			
Median (range)	62.0 (36–75)	60.0 (32–75)	63.0 (30–75)
<65 years, *n* (%)	150 (61.0)	160 (65.3)	147 (59.5)
≥65 years, *n* (%)	96 (39.0)	85 (34.7)	100 (40.5)
Sex, *n* (%)			
Male	209 (85.0)	204 (83.3)	207 (83.8)
Female	37 (15.0)	41 (16.7)	40 (16.2)
ECOG performance status, *n* (%)			
0	47 (19.1)	47 (19.2)	48 (19.4)
1	199 (80.9)	198 (80.8)	199 (80.6)
Smoking status, *n* (%)			
Never	59 (24.0)	59 (24.1)	54 (21.9)
Former	154 (62.6)	148 (60.4)	158 (64.0)
Current	33 (13.4)	38 (15.5)	35 (14.2)
Clinical stage^a^, *n* (%)			
Limited stage	1 (0.4)	3 (1.2)	7 (2.8)
ES	245 (99.6)	242 (98.8)	240 (97.2)
Disease stage^b^, *n* (%)			
Stage II	–	–	1 (0.04%)^c^
Stage III	30 (12.2)	26 (10.6)	21 (8.5)
Stage IV	216 (87.8)	219 (89.4)	225 (91.1)
Brain metastases^d^, *n* (%)	25 (10.2)	22 (9.0)	26 (10.5)
Liver metastases^d^, *n* (%)	79 (32.1)	78 (31.8)	79 (32.0)
Bone metastases^d^, *n* (%)	69 (28.0)	74 (30.2)	69 (27.9)
Previous therapy^e^, *n* (%)			
Surgery	2 (0.8)	5 (2.0)	6 (2.4)
Chemotherapy	2 (0.8)	3 (1.2)	7 (2.8)
Radiotherapy	4 (1.6)	5 (2.0)	6 (2.4)
Traditional Chinese medicine	9 (3.7)	10 (4.1)	14 (5.7)

Data are median (range) or *n* (%).

^a^Clinical stage was classified according to Veterans Administration Lung Study Group (VALG) criteria at first diagnosis.

^b^Disease stage was classified according to the American Joint Committee on Cancer (AJCC) 8th edition

^c^The tumor lesions of this stage II patient include the primary tumor in the right lung (tumor >3 cm but ≤4 cm in greatest dimension), regional lymph node metastasis in the ipsilateral hilar and inferior lobe, which cannot be delineated in a target area of radiation therapy and determined as ES-SCLC (assessed by IRC and investigator).

^d^Metastatic lesions were categorized on the basis of their presence or absence. Any patient with liver metastasis, regardless of whether it was the sole metastatic site or coexisting with other metastases, was included in the liver metastasis group for analysis.

^e^‘Previous therapy’ refers to the treatment previously for limited-stage SCLC.

Of the 738 enrolled patients, 736 patients received at least one dose of protocol treatment (safety population). After the completion of induction therapy, 214 patients in the benmelstobart and anlotinib plus EC group, 208 in the anlotinib plus EC group and 208 in the EC alone group received planned maintenance therapy. At the planned interim analysis for OS (data cutoff 14 May 2022), 89 patients are still ongoing the assigned treatment. The primary reason for treatment discontinuation was disease progression (56.1%, 67.8% and 78.5%, respectively) or death (3.3%, 3.3% and 2.8%, respectively). Among the patients who withdrew from the study, subsequent systemic therapy was administered to 42.7% of the patients in the benmelstobart and anlotinib plus EC group, 58.4% in the anlotinib plus EC group and 71.3% in the EC alone group (Extended Data Table 1).

### Efficacy

#### Primary endpoint of OS

**Fig. 2:**
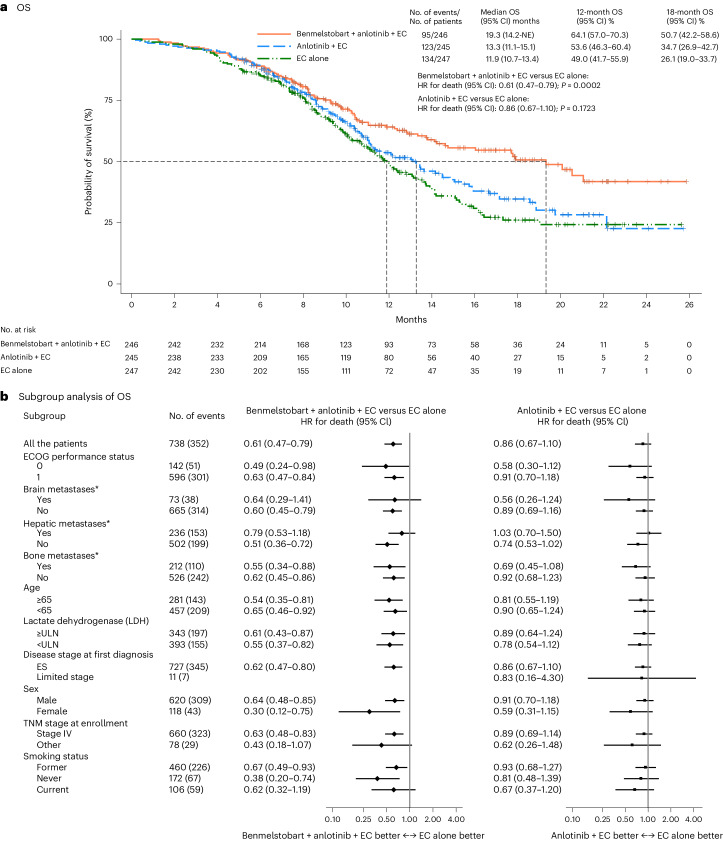
OS in ITT population. **a**, The Kaplan–Meier curves for OS in three treatment groups. The tick marks indicate censored data. Differences between the treatment groups were evaluated with the stratified log-rank test. *P* values are two-sided. **b**, The subgroup analysis of OS. A stratified Cox regression model was used to estimate the HR for death and 95% CIs. The circles indicate HR among subgroups of patients, the horizontal lines indicate corresponding 95% CIs and the vertical dotted line indicates the HR for the overall population. *Metastatic lesions were categorized on the basis of their presence or absence. Any patient with liver metastasis, regardless of whether it was the sole metastatic site or coexisting with other metastases, was included in the liver metastasis group for analysis. ULN, upper limit of normal; TNM, tumor, node, metastasis.

At the point of data cutoff, the median follow-up for OS was 14.0 months (95% confidence interval (CI) 12.8–15.5) for the ITT population. At the prespecified interim analysis for OS (at 352 deaths in the overall population; data cutoff 14 May 2022), median OS was greater in the benmelstobart and anlotinib plus EC group compared with the EC alone group (19.3 months (95% CI 14.2 to not estimable) versus 11.9 months (95% CI 10.7–13.4); hazard ratio (HR), 0.61 (95% CI 0.47–0.79); *P* = 0.0002), meeting the prespecified criteria for statistical significance (Fig. 2a). The anlotinib plus EC group had no significant OS benefit over the EC alone group (13.3 months (95% CI 11.1–15.1) versus 11.9 months (95% CI 10.7–13.4); HR, 0.86 (95% CI 0.67–1.10); *P* = 0.1723; Fig. 2a). The estimated OS rate at 12 months was 64.1% (95% CI 57.0–70.3), 53.6% (95% CI 46.3–60.4) and 49.0% (95% CI 41.7–55.9) and at 18 months was 50.7% (95% CI 42.2–58.6), 34.7% (95% CI 26.9–42.7) and 26.1% (95% CI 19.0–33.7) in the benmelstobart and anlotinib plus EC, anlotinib plus EC, and EC alone groups, respectively. OS favored the benmelstobart and anlotinib plus EC group or anlotinib plus EC group over the EC alone group across multiple prespecified subgroups (Fig. 2b).

#### Primary endpoint of PFS

**Fig. 3:**
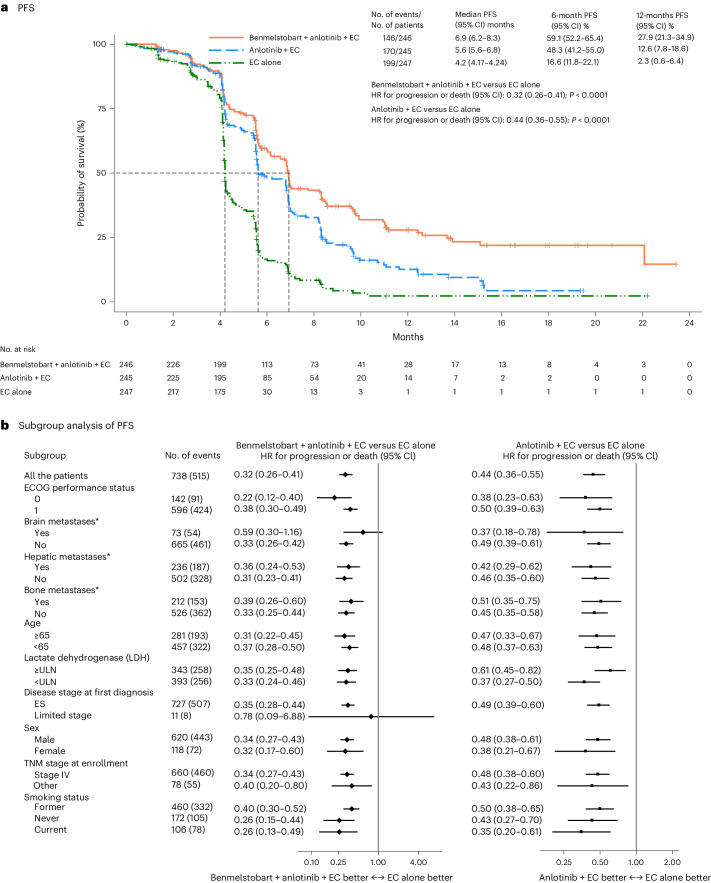
PFS in ITT population. **a**, The Kaplan–Meier curves for PFS in three treatment groups. The tick marks indicate censored data. PFS was assessed according to RECIST 1.1, by an independent review committee. Differences between the treatment groups were evaluated with the stratified log-rank test. *P* values are two-sided. **b**, The subgroup analysis of PFS. A stratified Cox regression model was used to estimate the HR for death and 95% CIs. The circles indicate HR among subgroups of patients, the horizontal lines indicate corresponding 95% CIs and the vertical dotted line indicates the HR for the overall population. *Metastatic lesions were categorized on the basis of their presence or absence. Any patient with liver metastasis, regardless of whether it was the sole metastatic site or coexisting with other metastases, was included in the liver metastasis group for analysis. ULN, upper limit of normal; TNM, tumor, node, metastasis.

Progression-free survival (PFS) was assessed by the Independent Review Committee (IRC). Here, we report the results of the prespecified final analysis for PFS after occurrence of 347 PFS events. With 515 events of disease progression or death, median IRC-assessed PFS was significantly longer, compared with the EC alone group, in both the benmelstobart and anlotinib plus EC group (6.9 months (95% CI 6.2–8.3) versus 4.2 months (95% CI 4.17–4.24); HR 0.32 (95% CI 0.26–0.41); *P* < 0.0001) and in the anlotinib plus EC group (5.6 months (95% CI 5.6–6.8) versus 4.2 months (95% CI 4.17–4.24); HR 0.44 (95% CI 0.36–0.55); *P* < 0.0001) (Fig. 3a). The PFS rate at 6 months was 59.1% (95% CI 52.2–65.4), 48.3% (95% CI 41.2–55.0) and 16.6% (95% CI 11.8–22.1) in the benmelstobart and anlotinib plus EC, anlotinib plus EC, and EC alone groups, respectively; at 12 months PFS rate was 27.9% (95% CI 21.3–34.9), 12.6% (95% CI 7.8–18.6) and 2.3% (95% CI 0.6–6.4), respectively. The PFS benefit of the benmelstobart and anlotinib plus EC group or anlotinib plus EC group was observed in all subgroups (Fig. 3b).

#### Secondary endpoints

The investigator assessment reported that both the benmelstobart and anlotinib plus EC group and the anlotinib plus EC group improved PFS compared with the EC alone group per Response Evaluation Criteria in Solid Tumors version 1.1 (RECIST 1.1) (Supplementary Fig. 1a) or Immunotherapy Response Evaluation Criteria in Solid Tumours (iRECIST) (Supplementary Fig. 1b); this was consistent with the IRC-assessed results based on RECIST 1.1. The percentage of patients with an IRC-assessed objective response was 81.3% (95% CI 75.9–86.0) in the benmelstobart and anlotinib plus EC group and 81.2% (95% CI 75.8–85.9) in the anlotinib plus EC group, which were both significantly higher than in the EC alone group (66.8%; 95% CI 60.6–72.6; comparative *P* = 0.0001 and *P* = 0.0003, respectively) (Table 2). Three (1.2%) patients in the benmelstobart and anlotinib plus EC group and one (0.4%) patient in the anlotinib plus EC group attained a complete response (Table 2). There was no significant difference in disease control rate between the benmelstobart and anlotinib plus EC and EC alone groups (90.7% versus 87.0%; *P* = 0.2003); however, a significant improvement with anlotinib plus EC compared with EC alone was observed (92.7% versus 87.0%; *P* = 0.0378). As assessed by IRC, the median duration of response (DoR) for all responders was 5.8 months (95% CI 5.5–7.2) in the benmelstobart and anlotinib plus EC group, 5.5 months (95% CI 4.3–5.6) in the anlotinib plus EC group, and 3.1 months (95% CI 2.9–4.1) in the EC alone group (Supplementary Fig. 2a). The results were consistent with those assessed by the investigators per either RECIST 1.1 or iRECIST (Supplementary Fig. 2b and Supplementary Tables 1 and 2).

**Table 2:** Tumor response as assessed by blinded independent central review using RECIST 1.1

	Benmelstobart + anlotinib + EC group (*N* = 246)	Anlotinib + EC group (*N* = 245)	EC alone group (*N* = 247)
Objective confirmed response			
No. of patients	200 (81.3)	199 (81.2)	165 (66.8)
95% CI	75.9–86.0	75.8–85.9	60.6–72.6
*P* value	0.0001	0.0003	Reference
Best objective response, no. (%)			
CR	3 (1.2)	1 (0.4)	0
PR	197 (80.1)	198 (80.8)	165 (66.8)
SD	23 (9.3)	28 (11.4)	50 (20.2)
PD	8 (3.3)	3 (1.2)	16 (6.5)
NE^a^	15 (6.1)	15 (6.1)	16 (6.5)

Differences in response rate between the treatment groups were assessed with the stratified Cochran–Mantel–Haenszel test. *P* values are two-sided.

^a^The best overall response could not be evaluated for patients who had no baseline or no postbaseline tumor assessments and at least one lesion that could not be evaluated.

CR, complete response; PR, partial response; SD, stable disease; PD, progressive disease; NE, not evaluated.

#### Safety

Treatment-emergent adverse events (TEAEs) of any grade (100.0% in each group) and of grade 3 or higher were similar (94.3%, 95.9% and 89.0%) in the benmelstobart and anlotinib plus EC, anlotinib plus EC, and EC alone groups, respectively (Supplementary Table 3), with the most common toxicities including thrombocytopenia, neutropenia and leukopenia (Extended Data Table 2). Treatment-related adverse events (TRAEs) were reported in 100.0%, 99.6% and 99.6% of patients in the three groups, respectively; of these, 93.1%, 94.3% and 87.0%, respectively, were grade 3 or higher, with hematologic toxicities and hypertension being the most common events (Table 3). TRAEs leading to benmelstobart/placebo interruption occurred similarly between benmelstobart and anlotinib plus EC (50.4%) and anlotinib plus EC (46.3%) groups, and were more common than in the EC alone (33.3%) group. The median time to first interruption of benmelstobart/placebo was 1.45 months (interquartile range (IQR) 0.49–2.60) in the benmelstobart and anlotinib plus EC group, 1.12 months (IQR 0.49–2.14) in the anlotinib plus EC group, and 1.35 months (IQR 0.49–2.46) in the EC alone group. A similar trend of TRAEs leading to anlotinib/placebo dose reduction or interruption was observed across three groups (61.4%, 61.1% and 37.0%, respectively), with a median time to first dose reduction/interruption of 1.38 months (IQR 0.49–2.66), 1.25 months (IQR 0.49–2.17) and 1.51 months (IQR 0.49–2.56), respectively. TRAEs caused death in 4.5%, 2.5% and 1.6% of patients in the benmelstobart and anlotinib plus EC, anlotinib plus EC, and EC alone groups, respectively. TRAEs leading to death are listed in Supplementary Table 4 and include pneumonitis (one, one and one patient), hemoptysis (two, one and zero), dyspnea/respiratory failure (one, two and zero) and sepsis (one, zero and zero) in the benmelstobart and anlotinib plus EC, anlotinib plus EC, and EC alone groups, respectively. Five patients had fatal TRAEs (attributable to pneumonitis, sepsis, empyema or acute heart failure and one patient with a combination of pulmonary infection with suspected paraneoplastic syndrome, encephalopathies and meningeal metastasis) in the benmelstobart and anlotinib plus EC group, and each one patient in other groups died due to EC-related AEs. Anlotinib-related AEs led to two deaths in the anlotinib plus EC group.

**Table 3:** Treatment-related adverse events

Events	Benmelstobart + anlotinib + EC group (*N* = 246)		Anlotinib + EC group (*N* = 244)		EC alone group (*N* = 246)	
	Any grade	Grade ≥3	Any grade	Grade ≥3	Any grade	Grade ≥3
Any TRAEs	246 (100.0)	229 (93.1)	243 (99.6)	230 (94.3)	245 (99.6)	214 (87.0)
Frequent TRAEs (≥10% incidence)						
Neutropenia	224 (91.1)	171 (69.5)	225 (92.2)	178 (73.0)	223 (90.7)	169 (68.7)
Leukopenia	223 (90.7)	94 (38.2)	217 (88.9)	75 (30.7)	225 (91.5)	85 (34.6)
Thrombocytopenia	219 (89.0)	122 (49.6)	220 (90.2)	131 (53.7)	201 (81.7)	88 (35.8)
Anemia	192 (78.0)	59 (24.0)	198 (81.2)	65 (26.6)	207 (84.2)	58 (23.6)
Nausea	99 (40.2)	1 (0.4)	107 (43.9)	2 (0.8)	108 (43.9)	0
Hypertension	77 (31.3)	38 (15.5)	66 (27.1)	29 (11.9)	18 (7.3)	4 (1.6)
Hypothyroidism	75 (30.5)	1 (0.4)	55 (22.5)	0	13 (5.3)	0
Anorexia	68 (27.6)	5 (2.0)	77 (31.6)	3 (1.2)	60 (24.4)	1 (0.4)
Alanine aminotransferase increased	68 (27.6)	2 (0.8)	64 (26.2)	2 (0.8)	73 (29.7)	5 (2.0)
Hypertriglyceridemia	67 (27.2)	8 (3.3)	82 (33.6)	20 (8.2)	46 (18.7)	2 (0.8)
Aspartate aminotransferase increased	66 (26.8)	3 (1.2)	62 (25.4)	3 (1.2)	60 (24.4)	1 (0.4)
Vomiting	62 (25.2)	1 (0.4)	60 (24.6)	2 (0.8)	70 (28.5)	1 (0.4)
Hypoalbuminemia	60 (24.4)	0	34 (13.9)	0	37 (15.0)	1 (0.4)
Proteinuria	59 (24.0)	2 (0.8)	53 (21.7)	1 (0.4)	28 (11.4)	1 (0.4)
Fatigue	57 (23.2)	6 (2.4)	51 (20.9)	4 (1.6)	42 (17.1)	0
Alopecia	55 (22.4)	0	47 (19.3)	0	44 (17.9)	1 (0.4)
Hypercholesterolemia	48 (19.5)	1 (0.4)	48 (19.7)	1 (0.4)	24 (9.8)	0
Weight loss	45 (18.3)	2 (0.8)	35 (14.3)	5 (2.1)	17 (6.9)	1 (0.4)
Diarrhea	44 (17.9)	2 (0.8)	39 (16.0)	3 (1.2)	16 (6.5)	2 (0.8)
Hyponatremia	39 (15.9)	11 (4.5)	38 (15.6)	9 (3.7)	39 (15.9)	12 (4.9)
Thyroid-stimulating hormone increased	33 (13.4)	0	41 (16.8)	0	8 (3.3)	0
Hemoptysis	33 (13.4)	3 (1.2)	30 (12.3)	3 (1.2)	18 (7.3)	2 (0.8)
Constipation	30 (12.2)	0	37 (15.2)	0	39 (15.9)	0
Hyperuricemia	30 (12.2)	0	30 (12.3)	0	31 (12.6)	0
Occult blood positive	29 (11.8)	0	26 (10.7)	0	17 (6.9)	2 (0.8)
Lymphocyte count decreased	29 (11.8)	8 (3.3)	24 (9.8)	7 (2.9)	24 (9.8)	7 (2.9)
Blood bilirubin increased	29 (11.8)	2 (0.8)	20 (8.2)	3 (1.2)	17 (6.9)	2 (0.8)
Palmar–plantar erythrodysesthesia syndrome	28 (11.4)	6 (2.4)	22 (9.0)	8 (3.3)	3 (1.2)	0
Gamma-glutamyltransferase increased	27 (11.0)	4 (1.6)	41 (16.8)	5 (2.1)	28 (11.4)	3 (1.2)
Fever	27 (11.0)	0	9 (3.7)	0	10 (4.1)	0
Hyperthyroidism	25 (10.2)	0	5 (2.1)	0	6 (2.4)	0
Alkaline phosphatase increased	22 (8.9)	2 (0.8)	29 (11.9)	0	19 (7.7)	0

Data are *n* (%).

Serious adverse events (SAEs) were reported in 135 (54.9%), 119 (48.8%) and 101 (41.1%) patients in the benmelstobart and anlotinib plus EC, anlotinib plus EC, and EC alone groups, respectively, with 115 (46.7%), 106 (43.4%) and 84 (34.1%), respectively, being grade 3 or higher. Of these, 83 (33.7%), 67 (27.5%) and 43 (17.5%), respectively, were considered to be benmelstobart/anlotinib-related SAEs. Immune-related adverse events (irAEs) assessed by investigators were more common in the benmelstobart and anlotinib plus EC group (42.7%) compared with the anlotinib plus EC group (27.5%) or the EC alone group (19.1%), with a similar observation for grade 3 or higher irAEs (16.7%, 8.2% and 6.9%, respectively). Commonly occurring irAEs in the benmelstobart and anlotinib plus EC group were hypothyroidism (19.9%) and immune-related pneumonitis (4.5%). Fatal irAEs in the benmelstobart and anlotinib plus EC group were immune-related pneumonitis, immune-mediated encephalopathies, acute heart failure, acute coronary syndrome and infectious pneumonia, and the irAE-associated death in the EC alone group was pneumonitis; no death due to irAEs occurred in the anlotinib plus EC group. Additional AE details are provided in Extended Data Table 2 and Supplementary Tables 3 and 4.

#### Health-related quality of life (HRQoL)

**Extended Data Fig. 1:**
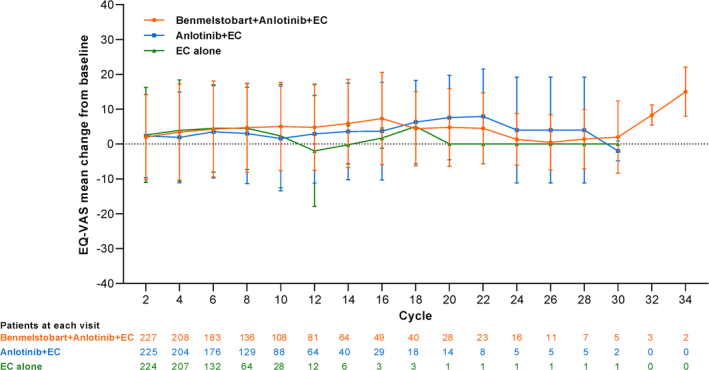
Patient-reported outcomes of health-related quality of life. Mixed model repeated measures change from baseline in least squares means for Benmelstobart and anlotinib plus EC, anlotinib plus EC, and EC alone in the overall population. Data are presented as mean values +/− standard deviation. At each cycle data are based on (n/N) participants where n denotes the number of participants completing a section of the questionnaire at a specific cycle and N the total number of participants reaching that cycle. Note: In the intention-to-treat population across all treatment groups, there was an overall increase in the mean change from baseline over time in EuroQol visual analogue scale (EQ-VAS) scores through week 34 (indicating improvements). But there were no significant differences in EQ-VAS scores between the study groups at each visit (*P* > 0.05 at all visits). Within the benmelstobart and anlotinib plus EC group, there was an improvement from baseline starting at cycle 2 (2.0 ± 12.2; *P* = 0.0128) through cycle 8 (4.7 ± 12.8; *P* < 0.0001). Within the anlotinib plus EC group, there was an improvement from baseline starting at cycle 2 (2.3 ± 11.9; *P* = 0.0052) through cycle 8 (3.0 ± 14.3; *P* = 0.0255). Within the EC group, there was an improvement from baseline starting at cycle 2 (2.6 ± 13.6; *P* = 0.0011) through cycle 6 (4.5 ± 12.5; *P* = 0.0001). EC = Etoposide/carboplatin.

There was an overall increase in the mean change from baseline over time in EuroQol visual analog scale (EQ-VAS) scores (indicating improvements) through week 34 in the ITT population across all treatment groups. There were no significant differences in EQ-VAS scores between the study groups (*P* > 0.05), which indicated that HRQoL was maintained during the treatment period (Extended Data Fig. 1).

## Discussion

Based on the final PFS analysis and interim OS analysis (the final OS will be confirmed after the prespecified 497 OS events have been reached), the ETER701 phase 3 trial demonstrated that benmelstobart and anlotinib plus EC provided significant improvements in survival compared with EC chemotherapy alone. The benmelstobart and anlotinib plus EC group had a median OS of 19.3 months and provided a 7.4-month prolongation versus EC alone. The median PFS with benmelstobart and anlotinib plus EC was 6.9 months, providing a 2.7**-**month prolongation compared with EC alone. The Kaplan–Meier survival curves displayed early (around month 4 for PFS and month 8 for OS) and sustained separation, with a more significant separation favoring benmelstobart and anlotinib plus EC as maintenance therapy continued. This effect in the maintenance period is similar to that observed in CASPIAN and IMpower133^3,4^, and may be related to early relapse, following good initial response, with cessation of chemotherapy in the control arm, and the maintenance therapy exerting benefit in a relatively large proportion of patients. Sustained responses were also observed with benmelstobart and anlotinib plus EC compared with EC alone, with a 14.5% increase in objective response rate (81.3% versus 66.8%) and a 2.7-month extension in median DoR (5.8 versus 3.1 months). This phase 3 trial demonstrated that the combination of anlotinib and immunochemotherapy could achieve both OS and PFS benefits for patients with ES-SCLC in the first-line setting.

The lack of OS benefit in the anlotinib plus EC arm may be partially explained by the difference in subsequent therapies between the anlotinib plus EC group (58.4%) and the EC alone group (71.3%; Extended Data Table 1). The other potential explanation is the potential synergistic effect of anti-angiogenesis and ICI therapy in the reprogramming of the tumor microenvironment; with anlotinib alone, we may not observe this long-term benefit. This concept has been explored in patients with advanced non-small-cell lung cancer^14–16^; IMpower150 demonstrated that the combination of an ICI plus anti-VEGF and chemotherapy was associated with greater OS than with anti-VEGF and chemotherapy (19.2 versus 14.7 months, respectively)^14^. So far, four phase 3 trials have reported positive findings for the efficacy of immunochemotherapy as first-line therapy for ES-SCLC^3–6^. The median OS in IMpower133^3^, CASPIAN^4^, CAPSTONE-1^5^ and ASTRUM-005^6^ was 12.3 months, 13.0 months, 15.3 months and 15.4 months, respectively. The median OS in the ETER701 trial was 19.3 months.

As reported previously, ICI treatment exhibits marginal effects on patients with SCLC with liver metastases^17^. The ETER701 trial included a patient population with liver metastases (32.1%) that was consistent with those in IMpower133, CASPIAN, CAPSTONE-1 and ASTRUM-005 (25.4–40.0%)^3–6^. Benmelstobart and anlotinib plus EC had a smaller effect on OS in patients with, compared with those without, liver metastases in the ETER701 trial. Similar trends of lower survival benefits from first-line immuno-oncology-based regimens in patients with liver metastases, compared with those without liver metastases, were demonstrated previously^3,5,17^. Increased risk of death in patients with SCLC receiving ICI treatment may be ascribed to inherent immunotolerant characteristics of the liver leading to an immunosuppressive microenvironment of tumor liver metastasis and thus constraining immunotherapy^18,19^.

The proportion of never-smokers in East Asian patients with SCLC has been reported to be higher than that in white patients^20^. The proportion of patients who had never smoked in ETER701 (24.0%) was higher than in IMpower133 (4.5%)^3^ and CASPIAN (8%)^4^ but was comparable to CAPSTONE-1 (22.0%)^5^ and ASTRUM-005 (20.8%)^6^, which were both conducted in Chinese populations. It is plausible to speculate that the high proportion of never-smokers may have contributed to the survival benefits. Although smoking history as a prognostic factor in SCLC is supported by several retrospective studies and meta-analyses^21,22^, the CASPIAN and ASTRUM-005 trials suggested a negative correlation^4,6^. Another potential explanation for the higher proportion of never-smokers in Chinese populations with SCLC is an early transformation of *EGFR* mutation-positive lung cancer. However, genomic analysis from ETER701 is not available at this stage, and data on this potential relationship are limited at present.

Post-study treatments may confound the true effect of first-line therapy on OS. In the ETER701 trial, 71.3% of patients who received EC alone also received post-study treatment, which was similar to the control arm in the CAPSTONE-1 (70%)^5^ and higher than reported in CASPIAN, ASTRUM-005 or IMpower133 (43.4–57.4%)^3,4,6^. The proportion of patients (42.7%) in the benmelstobart and anlotinib plus EC group who received post-study treatments in ETER701 was consistent with those with immunotherapy-based regimens in the CASPIAN (44%)^4^ and ASTRUM-005 studies (44.2%)^6^ and lower than in the IMpower133 (51.7%)^3^ and CAPSTONE-1 (59%)^5^. The percentage of cases receiving post-study immunotherapy with benmelstobart and anlotinib plus EC over the control arm in our trial was comparable to those in CAPSTONE-1^5^ and lower than in ASTRUM-005^6^. Because the improvements in OS in the benmelstobart and anlotinib plus EC group were observed in the absence of a high proportion of post-study treatment, this therapeutic approach in the first-line setting may predominantly contribute to significant OS prolongation.

A slightly higher incidence of grade 3 or higher TEAEs was observed with benmelstobart and anlotinib plus EC (94.3%) and anlotinib plus EC (95.9%) than with EC alone (89.0%), which was expected. Common TEAEs of grade 3 or higher across three groups, mainly including hematological toxicities and hypertension, were as expected and manageable. No unexpected AEs or new safety signals were identified. Hypertension, a known AE of anlotinib treatment, had a similar incidence and severity to that seen in previously published data^23^, suggesting that anlotinib did not increase the risk of toxicity in this combination. The incidence of fatal TRAEs is broadly comparable to data available from the phase 3 trials assessing PD-L1 agents in the treatment of ES-SCLC^3–5^. A total of 11 patients had treatment-related deaths in the benmelstobart and anlotinib plus EC group, which is probably related to the addition of anti-angiogenesis to therapy. Similar risks were observed in other anti-angiogenesis, immuno-oncology and chemotherapy combination studies such as IMpower150 and ORIENT-31^14–16^. Although SAEs (mainly hematological toxicities) occurred relatively frequently in two study arms, these events were resolved with supportive care or dose modifications. Study discontinuation or deaths due to benmelstobart/anlotinib-related AEs were infrequent. Despite a large proportion of patients (42.7%) experiencing benmelstobart-related irAEs, as assessed by investigators, relatively few incidences (16.7%) were scored as grade 3 or higher, which was comparable with other PD-L1 inhibitors. Dose reduction or interruption due to TRAEs occurred more frequently with benmelstobart and anlotinib plus EC (71.1%) and anlotinib plus EC (72.1%) compared with the EC alone (52.0%) group. Dose modification is a common strategy to maximize therapeutic benefit while reducing toxicity. When stratified by dose reduction/interruption status, patients who underwent dose modification had comparable survival outcomes compared with those without both in the benmelstobart and anlotinib plus EC group (median OS 17.91 versus 19.32 months, HR 1.05 (95% CI 0.66–1.66), *P* = 0.9997) and in the anlotinib plus EC group (median OS 13.27 versus 12.16 months, HR 1.16 (95% CI 0.77–1.76), *P* = 0.4726). Overall, the safety profile of combination therapy was generally consistent with the known profiles of each single agent^23,24^, as well as with the previously reported safety profiles for each combination^25,26^. The addition of benmelstobart plus anlotinib to EC in the first-line setting was associated with an acceptable safety profile in patients with ES-SCLC.

Several limitations should be acknowledged. This study provided no direct comparison with the current standard-of-care immunochemotherapy. At the time of trial design, study protocol approval (14 August 2019) and commencement, etoposide plus cisplatin/carboplatin or irinotecan plus cisplatin/carboplatin was recommended as first-line chemotherapy for patients with ES-SCLC (Grade I recommendation), while atezolizumab plus etoposide and carboplatin was a Grade III recommendation, according to the Chinese Society of Clinical Oncology Clinical Guidelines for Small-Cell Lung Cancer (version 2019). Since there were no PD-1/PD-L1 inhibitors for SCLC available in China at the point of approval of our study protocol (14 August 2019), the standard-of-care of EC chemotherapy, and not immunochemotherapy, was adopted as the control arm in ETER701. Subsequently, in the 2023 update to the Chinese Society of Clinical Oncology guidelines, immunochemotherapy has been recommended as the preferred first-line therapy option (Grade I); however, chemotherapy also remains one of the first-line recommended options.

In conclusion, the ETER701 trial met the primary endpoint at the preplanned interim analysis and confirmed that first-line therapy with benmelstobart and anlotinib plus EC significantly improved OS and PFS in patients with ES-SCLC. Anlotinib plus EC improved PFS and response rates compared with EC alone, but the difference in OS was not significant. The addition of anti-angiogenesis therapy to immunochemotherapy may represent an efficacious and safe approach to the management of patients with ES-SCLC.

## Methods

### Ethics statement

The trial was conducted per the principles of Good Clinical Practice guidelines and the Declaration of Helsinki. Approvals for the trial protocol (and any protocol modifications) were obtained from independent ethics committees of each participating center (listed in Supplementary Information), and central approval was obtained from the Jinlin Province Cancer Hospital Institutional Review Board (ethics approval no. 201909-053-01). All patients provided written informed consent for participation before enrollment. The trial was designed and conducted by a panel of academic advisors and the sponsor (Chia Tai Tianqing Pharmaceutical Group Co., Ltd.). An independent data and safety monitoring committee provided oversight of safety and efficacy at prespecified interim analysis. None of the study participants received compensation for participation in the study. This study is registered with ClinicalTrials.gov (NCT04234607).

### Trial design and patients

ETER701 was a multicenter, double-blind, randomized, placebo-controlled phase 3 trial conducted at 72 sites in China. Patients aged 18–75 years with histologically confirmed ES-SCLC (as defined according to the Veterans Administration Lung Study Group staging system^27^) were enrolled in this trial. Eligible patients received no previous systemic therapy for ES-SCLC and had an Eastern Cooperative Oncology Group performance status (ECOG PS) of 0 or 1, and at least one measurable lesion according to RECIST 1.1 (ref. ^28^). Patients with limited-stage SCLC who had relapsed over 6 months after radical surgery and adjuvant chemoradiotherapy were also eligible for inclusion. Other inclusion criteria included a predicted life expectancy of at least 3 months and adequate organ function. Key exclusion criteria included previous therapy with anti-angiogenic drugs or other PD-L1 inhibitors; brain metastasis (except for asymptomatic, or stable brain metastases after previous therapy) and/or cancerous meningitis; and active autoimmune diseases requiring systemic treatment that occurred within 2 years. Full eligibility criteria are listed in the full trial protocol (Supplementary Information).

### Randomization and masking

The study was conducted in a double-blind, double-dummy design. All eligible patients were centrally randomly assigned in a 1:1:1 ratio to receive one of the three regimens: benmelstobart, anlotinib and EC followed by benmelstobart and anlotinib maintenance (benmelstobart and anlotinib plus EC group); benmelstobart placebo, anlotinib and EC followed by anlotinib maintenance (anlotinib plus EC group); or placebos for both benmelstobart and anlotinib, plus EC followed by placebo maintenance (EC alone group). Randomization was conducted by the central stratified randomization method, and stratified by ECOG PS (0 versus 1), brain metastases (‘yes’ or ‘no’) and liver metastases (‘yes’ or ‘no’). The randomization list was generated by an independent statistician using a SAS statistical package (version 9.4, SAS Institute) and secured in the eRand central randomization system. Subjects enrolled in this trial and who met the inclusion/exclusion criteria were sequentially assigned to individual groups through the eRand central randomization system according to their randomization numbers. Patients and their families or guardians, investigators, local and central radiological reviewers, the study statistician, data management personnel who were involved in data cleaning and analysis of the data, and the study sponsor were masked to treatment allocation until the final database was locked. The interim analysis was conducted in a nonblinded manner by the Independent Data Monitoring Committee. The Independent Data Monitoring Committee consisted of two independent oncologists and one independent statistician and was independent of the sponsors and investigators.

### Procedures

The treatment procedure consisted of two phases: induction and maintenance (Supplementary Fig. 3). During the induction phase, patients received a protocol-specified treatment regimen for four cycles. Patients who achieved a complete response, partial response or stable disease with tolerable toxicity were scheduled for maintenance therapy according to the previous random assignment. Benmelstobart was given intravenously at a dose of 1,200 mg once daily on day 1 of each cycle. Anlotinib was given orally, once daily, at a dose of 12 mg for 2 weeks followed by 1 week off. Patients in all groups received etoposide 100 mg m^−2^ intravenously on days 1–3 of each cycle and carboplatin area under the curve of 5 mg ml^−1^ min^−1^ intravenously on day 1 of each cycle. All treatments were administered in 21-day cycles. Treatments continued until it was assessed that there was disease progression, a loss of clinical benefit or unacceptable toxicity; treatment could also be discontinued at the investigators’ discretion. Per protocol, the crossover between treatment groups within the trial was not permitted. Full details regarding dose reduction and interruptions are listed in ‘Methods’ section in Supplementary Protocol.

### Endpoints and assessments

The ETER701 trial assessed two primary efficacy endpoints: PFS assessed by an IRC per RECIST 1.1 (defined as the time from randomization to the first documented progressive disease or death from any cause) and OS (defined as the time from randomization to death from any cause). Secondary endpoints were as follows: PFS assessed by the investigator per RECIST 1.1 and iRECIST^29^; the IRC-assessed objective response rate as per RECIST 1.1 (defined as the percentage of participants achieving complete response and partial response); disease control rate (defined as the percentage of participants achieving a complete response, partial response, and stable disease); DoR (defined as the time from first documented evidence of complete response or partial response until progressive disease or death, whichever occurred first); 6-month and 12-month PFS probability; 12-month and 18-month OS probability; the HRQoL evaluated with the EQ-VAS questionnaire; and safety.

Tumor assessments per RECIST 1.1 and iRECIST were performed using computed tomography or magnetic resonance imaging at screening and every two cycles from randomization until imaging-based disease progression, the start of new antitumor treatments, consent withdrawal or death, whichever occurred first. Additional details regarding response assessments and brain and bone imaging are provided in the full trial protocol (Supplementary Information). Adverse events were monitored and graded according to the National Cancer Institute Common Terminology Criteria for Adverse Events (version 5.0).

### Statistical analysis

A fixed-sequence test was used for multiple testing between treatment groups (Supplementary Fig. 4). Testing begins with the first hypothesis, H1, and each test is carried out without a multiplicity adjustment, provided that significant results are observed in all preceding tests. The fixed-sequence procedure controls the family-wise error rate because, for each hypothesis, testing is conditional upon rejecting all hypotheses earlier in the sequence. The median PFS of 4 months and median OS of 10 months were expected in the EC alone group on the basis of existing data. Assuming a 12-month recruitment period, 18 months of follow-up and a 10% dropout rate, approximately 738 patients (combined sample size for PFS and OS) were needed for 1:1:1 randomization and to provide a power of 85% to detect an HR across treatment groups for disease progression or death at a two-sided significance level of 0.05. Sample size assumptions are provided in ‘Methods’ section in Supplementary Protocol.

The full statistical analysis plan prespecified the performance of one interim and final analysis for OS and PFS, respectively. The final analysis for PFS was planned after 347 progressive disease events occurred with an *α* level of 0.205 (one-sided). The interim analysis for OS was planned after approximately 348 events of death had occurred, with adjustments for multiple comparisons taken into account with the Lan–DeMets O’Brien–Fleming spending function. To account for the prespecified interim analysis of OS, the nominal significance level was 0.0074 (one-sided). The final analysis for OS was planned after 497 death events occurred with an *α* level of 0.0228 (one-sided). The PFS final analysis and the OS interim analysis were conducted simultaneously. The trial is continuing to evaluate outcomes with additional follow-up. All data reported herein were based on the interim analysis of OS and the final analysis of PFS.

Efficacy was assessed in the ITT population, which included all randomized patients. Safety was assessed in the safety set, which included all randomized patients who received at least one dose of the assigned therapy. Survival data were estimated with the Kaplan–Meier method and compared using the stratified log-rank test. HRs and associated 95% CIs were assessed using a stratified Cox proportional-hazards model with Efron’s method of handling ties. Differences in response rate were assessed with the stratified Cochran–Mantel–Haenszel test. The randomization stratification factors were applied to all stratified analyses. Survival was also analyzed in the per-protocol population (all randomized patients without major protocol deviation) as supportive results. Sensitivity analysis was done using an unstratified log-rank test and an unadjusted Cox proportional-hazards model. The mean change from baseline in the EQ-VAS was compared with the use of a mixed-model, repeated-measures method. Further details are available in the statistical analysis plan (Supplementary Information).

### Reporting summary

Further information on research design is available in the Nature Portfolio Reporting Summary linked to this article.

## Online content

Any methods, additional references, Nature Portfolio reporting summaries, source data, extended data, supplementary information, acknowledgements, peer review information; details of author contributions and competing interests; and statements of data and code availability are available at 10.1038/s41591-024-03132-1.

## Supplementary information

Supplementary Information Collaborators and Supplementary Figs. 1–4, Tables 1–4 and protocol.

Reporting Summary

## Figures and Tables

**Extended Data Table 1:**
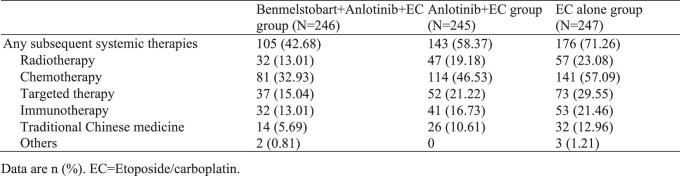
Summary of subsequent therapies during survival follow-up

**Extended Data Table 2:**
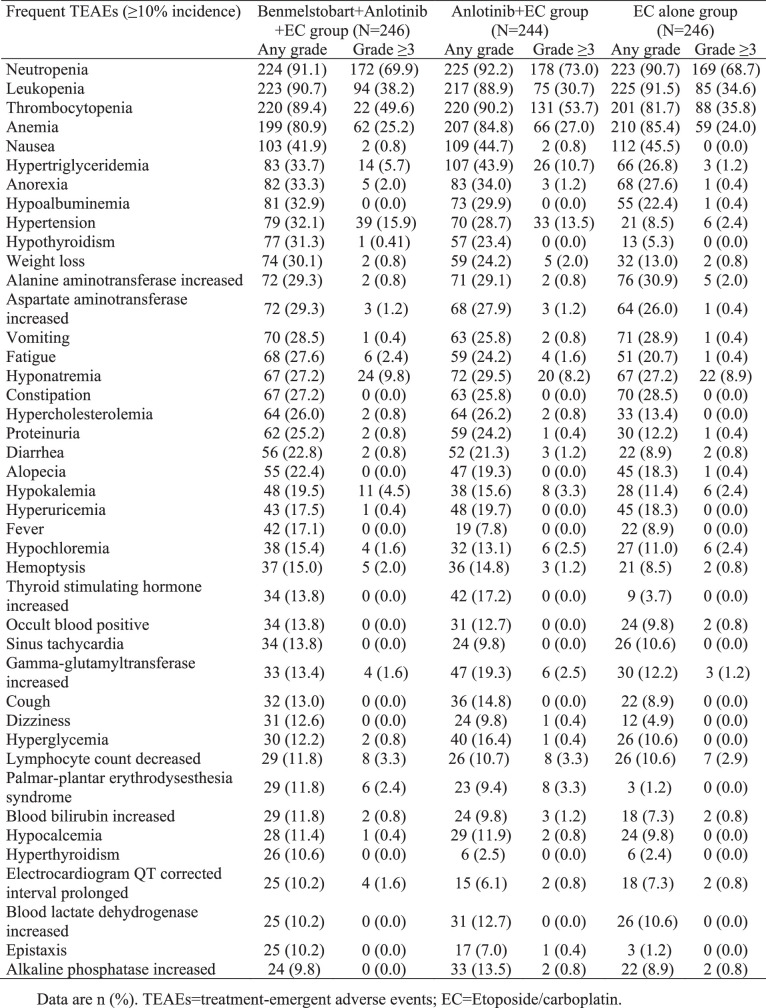
Frequent treatment-emergent adverse events reported in ≥10% of patients

## Data Availability

All data required to interpret, verify or build new research on the published claims are included in the article or uploaded in Supplementary Information. We cannot share individual deidentified participant data owing to the risk of reidentification and loss of patient confidentiality.
