# Supplementary material for: Benmelstobart, anlotinib and chemotherapy in extensive-stage small-cell lung cancer: a randomized phase 3 trial
Source: Nat Med. 2024 Jul 11;30(10):2967–76. doi: 10.1038/s41591-024-03132-1 (PMC11485241; doi:10.1038/s41591-024-03132-1)
Supplement: Supplementary file 1 — Collaborators and Supplementary Figs. 1–4, Tables 1–4 and protocol. [file 41591_2024_3132_MOESM1_ESM.pdf]

nature medicine

Supplementary information https://doi.org/10.1038/s41591-024-03132-1

Benmelstobart, anlotinib and chemotherapy in extensive-stage small-cell lung cancer: a randomized phase 3 trial

In the format provided by the authors and unedited

Benmelstobart, anlotinib and chemotherapy in extensive-stage small-cell lung cancer: a randomised phase 3 trial

Supplementary Materials

Collaborators 2

Supplementary Results 3

FIGURES........ 3

Figure S1. Progression-free survival as assessed by investigator review (intention-to-treat population).... 3

Figure S2. Duration of response as assessed by blinded independent central review and investigator review (intention-to-treat population)........ 4

Figure S3. Treatment procedure........ 5

Figure S4. Fixed-sequence test for multiple testing between treatment groups........ 5

TABLES 6

Table S1. Tumor response as assessed by investigator review using RECIST 1.1. 6

Table S2. Tumor response as assessed by investigator review using iRECIST. 6

Table S3. Safety summary........ 7

Table S4. Case summary for TRAEs, SAEs or irAEs leading to death. 8

Collaborators

Collaborator	Affiliation
Ying Cheng	Jilin Cancer Hospital, Changchun, China
Jianhua Chen	Hunan Cancer Hospital, Changsha, China
Wei Zhang	The First Affiliated Hospital of Nanchang University, Nanchang, China
Chao Xie	Shandong Cancer Hospital and Institute, Shandong University, Jinan, China
Qun Hu	The Affiliated Hospital of Inner Mongolia University, Hohhot, China
Ningning Zhou	Sun Yat-sen University Cancer Center, Guangzhou, China
Chun Huang	Tianjin Medical University Cancer Institute and Hospital, Tianjin, China
Shihong Wei	Gansu Provincial Cancer Hospital, Lanzhou, China
Hong Sun	The First Affiliated Hospital of Xi'an Jiaotong University, Xi'an, China
Xingya Li	The First Affiliated Hospital of Zhengzhou University, Zhengzhou, China
Yan Yu	Harbin Medical University Cancer Hospital, Harbin, China
Jinhuo Lai	Fujian Medical University Union Hospital, Fuzhou, China
Huaping Yang	Xiangya Hospital Central South University, Changsha, China
Haohui Fang	Anhui Chest Hospital, Hefei, China
Hualin Chen	Affiliated Hospital of Guangdong Medical University, Zhanjiang, China
Peng Zhang	Shanghai Pulmonary Hospital, Shanghai, China
Kangsheng Gu	The First Affiliated Hospital of Anhui Medical University, Hefei, China
Qiming Wang	Henan Cancer Hospital, Affiliated Cancer Hospital of Zhengzhou University, Zhengzhou, China
Jianhua Shi	Linyi Cancer Hospital, Linyi, China
Tienan Yi	Xiangyang Central Hospital, Xiangyang, China
Xingxiang Xu	Northern Jiangsu People's Hospital, Yangzhou, China
Xianwei Ye	Guizhou Provincial People's Hospital, Guiyang, China
Daqing Wang	Hengshui People's Hospital, Hengshui, China
Conghua Xie	Zhongnan Hospital of Wuhan University, Wuhan, China
Chunling Liu	Cancer Hospital Affiliated to Xinjiang Medical University, Urumqi, China
Yulong Zheng	The First Affiliated Hospital, Zhejiang University School of Medicine, Hangzhou, China
Daren Lin	Jiangmen Central Hospital, Jiangmen, China
Wu Zhuang	Fujian Cancer Hospital, Fuzhou, China
Ping Lu	The First Affiliated Hospital of Xinxiang Medical College, Xinxiang, China
Guohua Yu	Weifang People's Hospital, Weifang, China
Jinzhang Li	Qinghai University Affiliated Hospital, Xining, China
Yuhai Gu	Qinghai Provincial People's Hospital, Xining, China
Baolan Li	Beijing Chest Hospital, Capital Medical University, Beijing, China
Rong Wu	Shengjing Hospital of China Medical University, Shenyang, China
Ou Jiang	The Second People's Hospital of Neijiang, Neijiang, China
Zaiyi Wang	The First Affiliated Hospital of Xinjiang Medical University, Urumqi, China
Guowu Wu	Meizhou People's Hospital, Meizhou, China
Haifeng Lin	The Second Affiliated Hospital of Hainan Medical University, Haikou, China
Diansheng Zhong	Tianjin Medical University General Hospital, Tianjin, China
Yanhua Xu	Jingzhou Central Hospital, Jingzhou, China
Yongqian Shu	Jiangsu Province Hospital, Nanjing, China
Di Wu	Shenzhen People's Hospital, Shenzhen, China
Xingwu Chen	The First Affiliated Hospital of Wannan Medical College, Wuhu, China
Jie Wang	Cancer Hospital, Chinese Academy of Medical Sciences, Beijing, China
Minghui Wang	Sun Yat-sen Memorial Hospital, Sun Yat-sen University, Guangzhou, China
Runxiang Yang	Yunnan Cancer Hospital, Kunming, China

Supplementary Results

FIGURES

Figure S1. Progression-free survival as assessed by investigator review (intention-to-treat population).
Panel A shows the Kaplan–Meier curves for progression-free survival as assessed per RECIST 1.1, and Panel B shows the Kaplan–Meier curves for progression-free survival as assessed per iRECIST. Progression-free survival was estimated with the Kaplan–Meier method and compared between treatment groups using the stratified log-rank test. Hazard ratios and associated 95% two-sided confidence intervals were estimated by a Cox proportional hazards model. Stratification factors included Eastern Cooperative Oncology Group performance status, brain metastases, and liver metastases. *P* values are two-sided. Tick marks indicate censored data. EC=Etoposide/carboplatin.

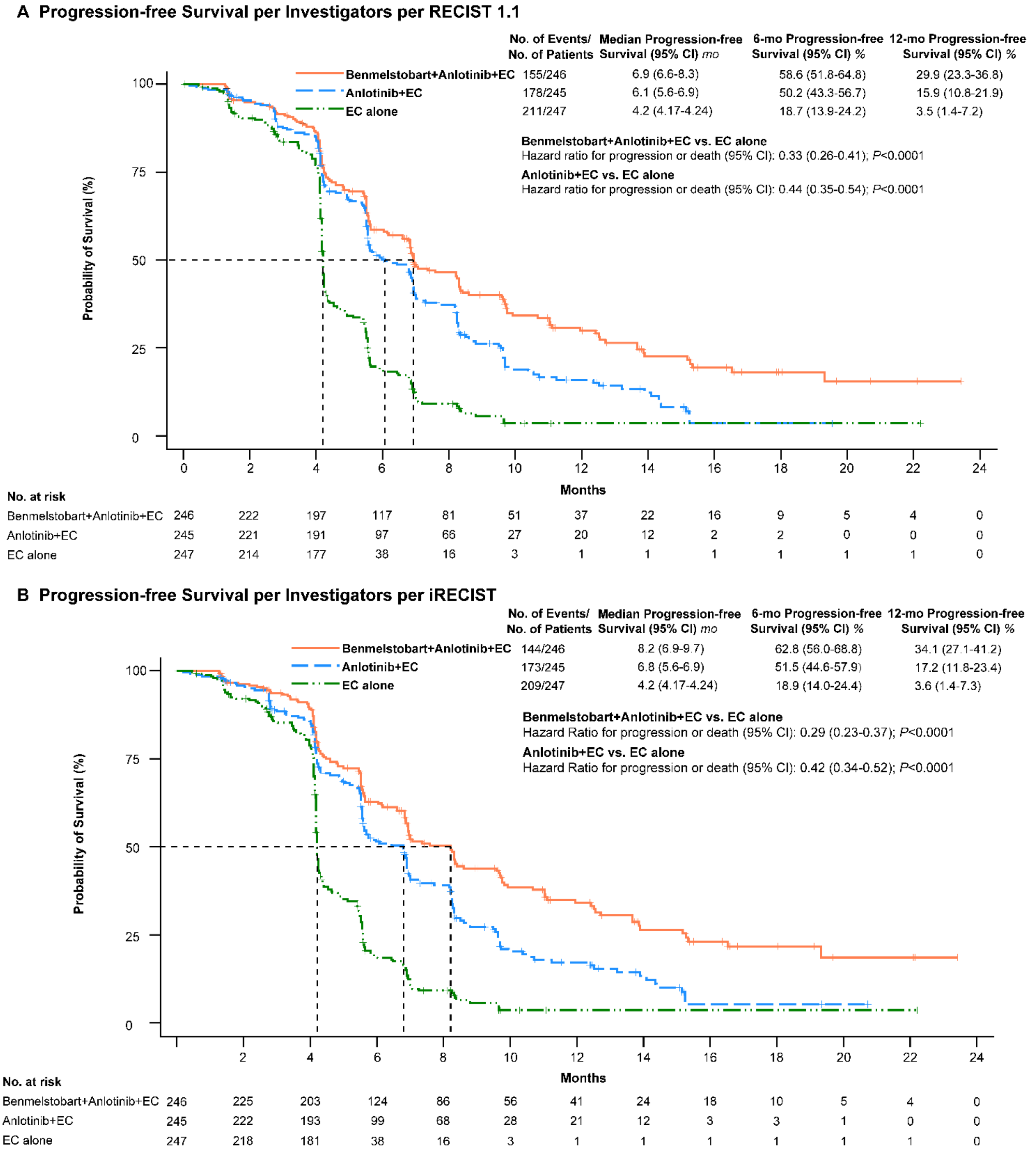

Figure S2. Duration of response as assessed by blinded independent central review and investigator review (intention-to-treat population).

Panel A shows the Kaplan–Meier curves for duration of response in patients who had a response assessed by blinded independent central review, and Panel B shows the Kaplan–Meier curves for duration of response in patients who had a response assessed by investigator review. This analysis included only patients who had an objective response as per RECIST 1.1. Duration of response was estimated with the Kaplan–Meier method and compared between treatment groups using the stratified log-rank test. Stratification factors included Eastern Cooperative Oncology Group performance status, brain metastases, and liver metastases. Hazard ratios and associated 95% two-sided confidence intervals were estimated by a Cox proportional hazards model. *P* values are two-sided. Tick marks indicate censored data. EC=Etoposide/carboplatin.

A Duration Response per Independent Review Committee

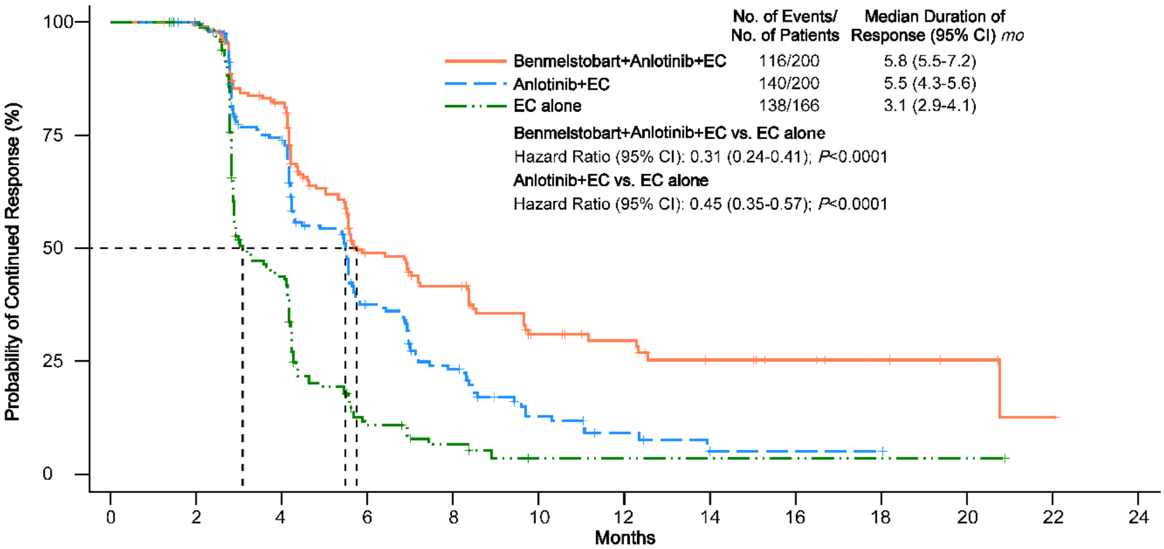

No. at risk	0	2	4	6	8	10	12	14	16	18	20	22	24
Benmelstobart+Anlotinib+EC	200	194	149	69	53	27	22	13	10	5	3	1	0
Anlotinib+EC	200	191	126	53	28	12	6	1	1	1	0	0	0
EC alone	166	162	65	12	5	1	1	1	1	1	1	0	0

B Duration Response per Investigators

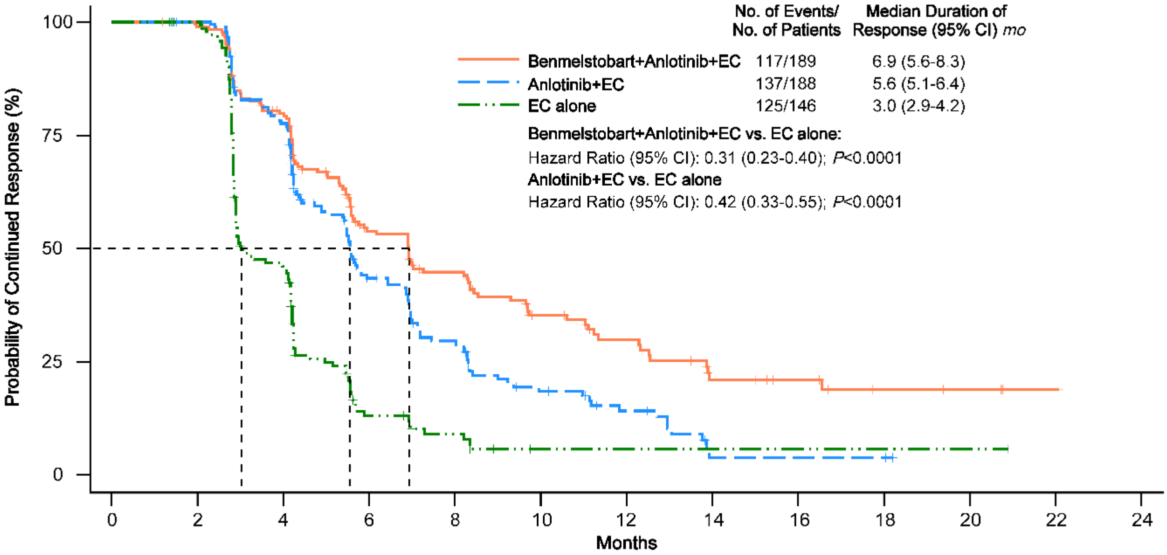

No. at risk	0	2	4	6	8	10	12	14	16	18	20	22	24
Benmelstobart+Anlotinib+EC	189	184	142	78	60	36	27	14	11	6	4	1	0
Anlotinib+EC	188	183	134	62	37	20	12	2	2	2	0	0	0
EC alone	146	142	63	15	8	1	1	1	1	1	1	0	0

Figure S3. Treatment procedure.

EC=Etoposide/carboplatin; CR=complete response; PR=partial response; SD=stable disease; PD=progressive disease; AUC=area under the curve.

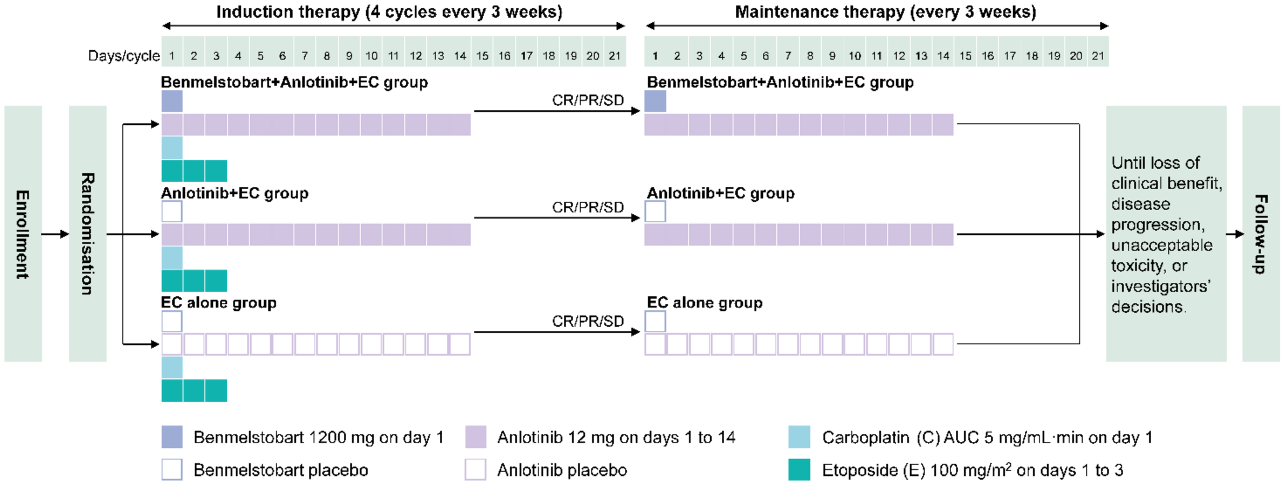

Figure S4. Fixed-sequence test for multiple testing between treatment groups.

A fixed-sequence test was used for multiple testing between treatment groups. Testing begins with the first hypothesis, H1, and each test is carried out without a multiplicity adjustment, provided that significant results are observed in all preceding tests. The fixed-sequence procedure controls the family-wise error rate because, for each hypothesis, testing is conditional upon rejecting all hypotheses earlier in the sequence. P_1=the one-side P value in the interim analysis; P_2=the one-side P value in the final analysis. A two-sided test with a significance level of α=0.05 was used for secondary efficacy endpoints and safety analysis. Two-sided P≤0.05 was considered statistically significant with a 95% CI.

OS=overall survival; PFS=progression-free survival.

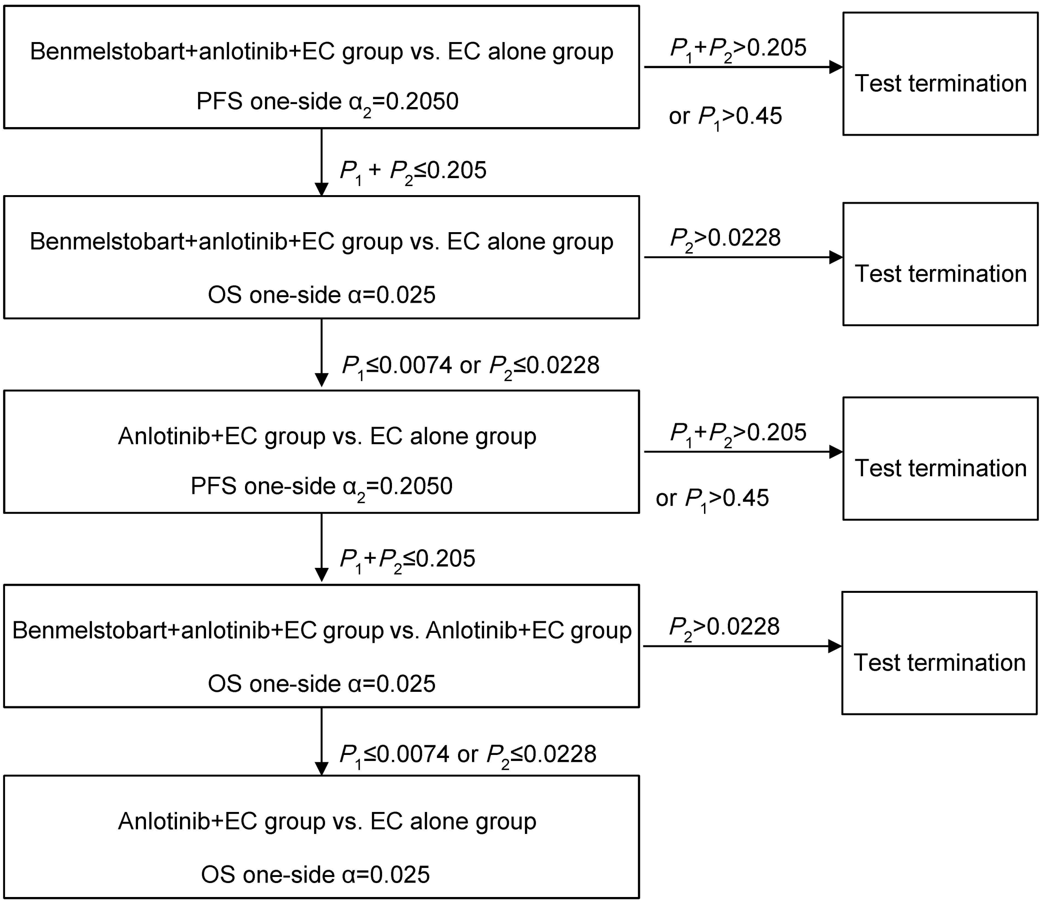

TABLES

Table S1. Tumor response as assessed by investigator review using RECIST 1.1.

	Benmelstobart+Anlotinib+EC group (N=246)	Anlotinib+EC group (N=245)	EC alone group (N=247)
Objective confirmed response			
No. of patients	188 (76.4)	187 (76.3)	145 (58.7)
95% CI	70.6-81.6	70.5-81.5	52.3-64.9
P value	<0.0001	<0.0001	Reference
Best objective response-no. (%)			
CR	1 (0.4)	1 (0.4)	0
PR	187 (76.0)	186 (75.9)	145 (58.7)
SD	32 (13.0)	36 (14.7)	64 (25.9)
PD	12 (4.9)	7 (2.9)	23 (9.3)
NE*	14 (5.7)	15 (6.1)	15 (6.1)

Differences in response rate between the treatment groups were assessed with the stratified Cochran-Mantel-Haenszel test. *P* values are two-sided.

* The best overall response could not be evaluated for patients who had no baseline or no postbaseline tumor assessments, and at least one lesion that could not be evaluated.
No.=number; CI, confidence interval; CR=complete response; PR=partial response; SD=stable disease; PD=progressive disease; NE=Not evaluated; EC=Etoposide/carboplatin.

Table S2. Tumor response as assessed by investigator review using iRECIST.

	Benmelstobart+Anlotinib+EC group (N=246)	Anlotinib+EC group (N=245)	EC alone group (N=247)
Objective response			
No. of patients	157 (63.8)	139 (56.7)	112 (45.3)
95% CI	57.5-69.8	50.3-63.0	39.0-51.8
P value	<0.0001	0.0114	Reference
Best objective response-no. (%)			
CR/iCR	1 (0.4)	0	0
PR/iPR	156 (63.4)	139 (56.7)	112 (45.3)
SD/iSD	27 (11.0)	29 (11.8)	54 (21.9)
PD	5 (2.03)	3 (1.2)	12 (4.9)
iUPD	14 (5.7)	13 (5.3)	8 (3.2)
iCPD	29 (11.8)	46 (18.8)	46 (18.6)
NE*	14 (5.7)	15 (6.1)	15 (6.1)

Differences in response rate between the treatment groups were assessed with the stratified Cochran-Mantel-Haenszel test. *P* values are two-sided.

* The best overall response could not be evaluated for patients who had no baseline or no postbaseline tumor assessments, and at least one lesion that could not be evaluated.

No.=number; CI, confidence interval; CR=complete response; iCR=immune complete response; PR=partial response; iPR=immune partial response; SD= stable disease; iSD=immune stable disease; PD=progressive disease; iUPD=immune unconfirmed progressive disease; iCPD=immune confirmed progressive disease; EC=Etoposide/carboplatin.

Table S3. Safety summary.

	Benmelstobart+Anlotinib+EC group (N=246)		Anlotinib+EC group (N=244)		EC alone group (N=246)	
	Any grade	Grade ≥3	Any grade	Grade ≥3	Any grade	Grade ≥3
**Any TEAEs**	246 (100.0)	232 (94.3)	244 (100.0)	234 (95.9)	246 (100.0)	219 (89.0)
TEAEs leading to any dose reduction or interruption	180 (73.2)	137 (55.7)	178 (73.0)	142 (58.2)	136 (55.3)	108 (43.9)
TEAEs leading to any discontinuation	49 (19.9)	37 (15.0)	37 (15.2)	26 (10.7)	26 (10.6)	16 (6.5)
TEAEs leading to death	26 (10.6)	26 (10.6)	15 (6.2)	15 (6.2)	17 (6.9)	17 (6.9)
**Any TRAEs**	246 (100.0)	229 (93.1)	243 (99.6)	230 (94.3)	245 (99.6)	214 (87.0)
TRAEs leading to any dose reduction or interruption	175 (71.1)	135 (54.9)	176 (72.1)	140 (57.4)	128 (52.0)	103 (41.9)
Benmelstobart/anlotinib-related AEs leading to dose reduction or interruption	124 (50.4)	83 (33.7)	116 (47.5)	79 (32.4)	57 (23.2)	39 (15.9)
TRAEs leading to benmelstobart/placebo interruption	124 (50.4)	87 (35.4)	113 (46.3)	80 (32.8)	82 (33.3)	55 (22.4)
TRAEs leading to anlotinib/placebo dose reduction or interruption	151 (61.4)	108 (43.9)	149 (61.1)	109 (44.7)	91 (37.0)	66 (26.8)
TRAEs leading to chemotherapy dose reduction or interruption	125 (50.8)	100 (40.7)	128 (52.5)	104 (42.6)	101 (41.1)	83 (33.7)
TRAEs leading to any discontinuation	41 (16.7)	29 (11.8)	30 (12.3)	21 (8.6)	15 (6.1)	10 (4.1)
TRAEs leading to benmelstobart/placebo discontinuation	32 (13.0)	23 (9.3)	15 (6.1)	12 (4.9)	11 (4.5)	8 (3.3)
TRAEs leading to anlotinib/placebo discontinuation	30 (12.2)	21 (8.5)	25 (10.2)	16 (6.6)	11 (4.5)	7 (2.8)
TRAEs leading to chemotherapy discontinuation	9 (3.7)	7 (2.8)	10 (4.1)	8 (3.3)	2 (0.8)	2 (0.8)
Benmelstobart/anlotinib-related AEs leading to discontinuation	21 (8.5)	14 (5.7)	17 (7.0)	11 (4.5)	7 (2.8)	5 (2.0)
Anlotinib-related AEs leading to discontinuation	3 (1.2)	1 (0.4)	11 (4.5)	7 (2.8)	2 (0.8)	1 (0.4)
TRAEs leading to death	11 (4.5)	11 (4.5)	6 (2.5)	6 (2.5)	4 (1.6)	4 (1.6)
Benmelstobart/anlotinib-related AEs leading to death	5 (2.0)	5 (2.0)	4 (1.6)	4 (1.6)	1 (0.4)	1 (0.4)
Anlotinib-related AEs leading to death	0	0	2 (0.8)	2 (0.8)	0	0
**Any SAEs**	135 (54.9)	115 (46.7)	119 (48.8)	106 (43.4)	101 (41.1)	84 (34.1)
Benmelstobart/anlotinib-related SAEs	83 (33.7)	67 (27.2)	67 (27.5)	57 (23.4)	43 (17.5)	35 (14.2)
SAEs leading to any dose reduction or interruption	103 (41.9)	85 (34.6)	92 (37.7)	79 (32.4)	69 (28.0)	59 (24.0)
SAEs leading to benmelstobart/placebo interruption	80 (32.5)	61 (24.8)	75 (30.7)	64 (26.2)	48 (19.5)	38 (15.4)
SAEs leading to anlotinib/placebo dose reduction or interruption	81 (32.9)	66 (26.8)	80 (32.8)	68 (27.9)	47 (19.1)	40 (16.3)
SAEs leading to chemotherapy dose reduction or interruption	72 (29.3)	60 (24.4)	67 (27.5)	60 (24.6)	53 (21.5)	46 (18.7)
SAEs leading to any discontinuation	36 (14.6)	30 (12.2)	24 (9.8)	20 (8.2)	18 (7.3)	13 (5.3)
SAEs leading to benmelstobart/placebo discontinuation	32 (13.0)	27 (11.0)	19 (7.8)	16 (6.6)	15 (6.1)	13 (5.3)
SAEs leading to anlotinib/placebo discontinuation	28 (11.4)	23 (9.3)	21 (8.6)	17 (7.0)	16 (6.5)	11 (4.5)
SAEs leading to chemotherapy discontinuation	10 (4.1)	10 (4.1)	9 (3.7)	8 (3.3)	5 (2.0)	4 (1.6)
SAEs leading to death	26 (10.6)	24 (9.8)	15 (6.1)	15 (6.1)	17 (6.9)	17 (6.9)

**Any irAEs***	105 (42.7)	41 (16.7)	67 (27.5)	20 (8.2)	47 (19.1)	17 (6.9)
irAEs leading to any dose reduction or interruption	47 (19.1)	26 (10.6)	22 (9.0)	10 (4.1)	12 (4.9)	8 (3.3)
irAEs leading to benmelstobart/placebo interruption	40 (16.3)	20 (8.1)	15 (6.1)	6 (2.5)	8 (3.3)	6 (2.4)
irAEs leading to anlotinib/placebo dose reduction or interruption	37 (15.0)	24 (9.8)	17 (7.0)	9 (3.7)	9 (3.7)	8 (3.3)
irAEs leading to chemotherapy dose reduction or interruption	20 (8.1)	12 (4.9)	7 (2.9)	3 (1.2)	11 (4.5)	7 (2.8)
irAEs leading to any discontinuation	20 (8.1)	15 (6.1)	3 (1.2)	2 (0.8)	4 (1.6)	3 (1.2)
irAEs leading to benmelstobart/placebo discontinuation	17 (6.9)	12 (4.9)	2 (0.8)	2 (0.8)	3 (1.2)	3 (1.2)
irAEs leading to anlotinib/placebo discontinuation	14 (5.7)	11 (4.5)	2 (0.8)	1 (0.4)	2 (0.8)	1 (0.4)
irAEs leading to chemotherapy discontinuation	1 (0.4)	1 (0.4)	0	0	1 (0.4)	1 (0.4)
irAEs leading to death	5 (2.0)	5 (2.0)	0	0	1 (0.4)	1 (0.4)

Data are n (%). * irAEs were assessed by investigators at each participating centers through review of patient-reported symptoms and laboratory results.

TEAEs=Treatment-emergent adverse events; TRAEs=Treatment-related adverse events; SAEs=serious adverse events; irAEs=immune-related adverse events; EC=Etoposide/carboplatin.

Table S4. Case summary for TRAEs, SAEs or irAEs leading to death.

**TRAEs leading to death**								
**Benmelstobart+Anlotinib+EC group**			**Anlotinib+EC group**			**EC alone group**		
Patient ID	Events	Causality*	Patient ID	Events	Causality*	Patient ID	Events	Causality*
13013	Hemoptysis	[5]	50003	Hepatic dysfunction	[4]	24003	Pneumonitis	[4]
21002	Immune-related pneumonitis	[4]	61011	Respiratory failure	[9]	26043	Death (NOS)	[4]
36020	Death (NOS)	[1]	63001	Interstitial pneumonitis	[7]	45004	Septic shock	[9]
40025	Pulmonary infection, paraneoplastic syndrome (suspected), immune-related encephalopathies (suspected), meningeal metastasis (suspected), impaired consciousness	[7]	68006	Hemoptysis	[8]	62005	Death (NOS)	[4]
44003	Death (NOS)	[1]	81002	Acute respiratory failure	[7]			
47003	Dyspnea	[10]	92007	Death (NOS)	[8]			
61013	Sepsis, septic shock, heart failure	[7]						
72006	Empyema, shock	[4]						
86001	Acute heart failure, myocarditis, cardiogenic shock	[1]						
91006	Acute coronary syndrome, diabetic ketoacidosis	[4]						
92005	Death (NOS), hemoptysis	[5]						

**SAEs leading to death**								
08003/10007/21005/26013/26018/36003/49003/77001/81008	PD	[11]	01033	Respiratory failure	[11]	01029/26025/26027/26029/26041/49005/61004/77010	PD	[11]
13001/20018/36020/44003/56006/92005	Death (NOS)	[11]	26002/26042/40011/40022/44002	PD	[11]	13005/26009/26043/62005/92008	Death (NOS)	[11]
13013/53012/92005	Hemoptysis	[5]	37002/61011/81002	Respiratory failure	[5]	15019	Pleural effusion	[11]
21002	Immune-related pneumonitis	[3]	50003	Hepatic dysfunction	[4]	24003	Pneumonitis	[4]
36016	Traffic accident	[11]	63001	Interstitial pneumonitis	[7]	31003	Acute myocardial infarction	[11]
40025	Infectious pneumonia, paraneoplastic syndrome, Immune-related encephalopathy, convulsion	[11]	63003/67001/92007	Death (NOS)	[11]	45004	Septic shock	[9]
47003	Dyspnea	[9]	68006	Hemoptysis	[8]			
61013	Sepsis, Septic shock, heart failure	[6]						
72006	Infectious pleural effusion, shock	[3]						
72009	Central nervous system metastasis	[11]						
86001	Acute heart failure, myocarditis, cardiogenic shock	[2]						
91006	Acute coronary syndrome, diabetic ketoacidosis	[4]						
92005	Death (NOS), hemoptysis	[5]						
**irAEs leading to death**								
21002	Immune-related pneumonitis	[11]				24003	Pneumonitis	[4]
40025	Infectious pneumonia, paraneoplastic syndrome, Immune-related encephalopathy, convulsion	[11]						
61013	Sepsis, septic shock, heart failure	[7]						

86001	Acute heart failure, myocarditis, cardiogenic shock	[1]
91006	Acute coronary syndrome, diabetic ketoacidosis	[4]

* Causality: [1] Related to benmelstobart, anlotinib, and EC; [2] Potentially related to benmelstobart, anlotinib and EC; [3] Related to benmelstobart and anlotinib; [4] Potentially related to benmelstobart and anlotinib; [5] Potentially related to anlotinib and EC; [6] Related to benmelstobart; [7] Potentially related to benmelstobart; [8] Potentially related to anlotinib; [9] Related to EC; [10] Potentially related to EC; [11] Unrelated to study drugs;
TRAEs=treatment-related adverse events; SAEs=serious adverse events; irAEs= Immune-mediated adverse events; EC=Etoposide/carboplatin; NOS, not otherwise specified; PD, disease progression

Clinical Study Protocol

Study Title: A Phase III Randomized, Double-blind, Parallel controlled, Multi-Center Trial of TQB2450 or Placebo plus Anlotinib and Etoposide/Carboplatin (EC) versus EC as First-line Therapy in Extensive-stage Small Cell Lung Cancer

Protocol Number: TQB2450-III-04

Version: 3.0 (6/6/2022)

Principal Center: Jilin Cancer Hospital

Principal Investigator: Ying Cheng

Participating Sites: Multicenter

Unit of Data Management: Chia Tai Tianqing Pharmaceutical Group Co., Ltd.

Statistician: Department of Biostatistics, School of Public Health, Nanjing Medical University

Sponsor: Chia Tai Tianqing Pharmaceutical Group Co., Ltd.

NO. 1099, Fuying Road, Jiangning District, Nanjing 210046, Jiangsu Province, China.
Tel: +86 025-85109999

Web: http://www.njcttq.com/

Confidential

This document contains confidential information and is only used by clinical researchers. The copyright is owned by Chia Tai Tianqing Pharmaceutical Group Co., Ltd. No information contained in this document may be released or disclosed to any third party (group or individual) without prior written permission. If you have this document without prior authorization, please contact Chia Tai Tianqing Pharmaceutical Group Co., Ltd., and return the document and its copy to the company.

Organization Structure Information Page

This study will be conducted by the clinical trial sponsor, implementation center, data management center, statistical analysis center, and experimental testing.

Sponsor of the clinical trial: Chia Tai Tianqing Pharmaceutical Co., Ltd.

Address: NO. 1099, Fuying Road, Jiangning District, Nanjing 210046, Jiangsu Province, China.

Responsibilities: Responsible for initiating and applying for work, providing investigator manuals, research drugs, and trial funding as well as being responsible for organizing, monitoring, auditing, and assisting in the design of clinical trial protocols.

The main investigators are as follows:

Position	Name	Telephone	E-mail
Chief of Project	Yue-Zhen Gao	18913929597	gaozhenyue2010@163.com
Chief of Medicine	Ding Yu	18251887468	ding.yu@cttq.com

- Clinical research implementation center (leader): Jilin Cancer Hospital

  Address: No.1018, Huguang Road, Changchun

  Main responsibilities: Recruiting subjects, designing clinical study protocols, organizing and implementing studies, and accepting inspections by regulatory authorities

  Principal investigator: Ying Cheng

  Telephone: 0431-85871902

- Clinical research data management center: Chia Tai Tianqing Pharmaceutical Co., Ltd.

  Address: NO. 1099, Fuying Road, Jiangning District, Nanjing

  Main responsibilities: Responsible for data management

  Responsible person: Ya-Dong Miu

  Telephone: 18551674600

- Clinical research statistics center: Department of Biostatistics, School of Public Health, Nanjing Medical University

  Address: NO. 818, Tianyuan East Road, Jiangning District, Nanjing

  Main responsibilities: Responsible for statistical analysis of clinical trials

  Responsible person: Hao Yu

  Telephone: 13814056262

Sponsor Signature Page

Protocol Title: A Phase III Randomized, Double-blind, Parallel controlled, Multi-Center Trial of TQB2450 or Placebo plus Anlotinib and Etoposide/Carboplatin (EC) versus EC as First-line Therapy in Extensive-stage Small Cell Lung Cancer

Protocol Number: TQB2450-III-04

Version: 3.0 (6/6/2022)

Sponsor: Chia Tai Tianqing Pharmaceutical Group Co., Ltd.

We have read and confirmed this protocol (Protocol Number: TQB2450-III-04; Version: 3.0; Version Date: 6/6/2022). We agree to fulfill the responsibilities of the sponsor in accordance with Chinese laws, the Helsinki Declaration, the Chinese GCP, and this protocol.

Sponsor's Authorized Representative: Xun-Qiang Wang

Print/Type Name

Signed: ____________________ **Date:** ______________________

Investigator's Agreement

Protocol Title: A Phase III Randomized, Double-blind, Parallel controlled, Multi-Center Trial of TQB2450 or Placebo plus Anlotinib and Etoposide/Carboplatin (EC) versus EC as First-line Therapy in Extensive-stage Small Cell Lung Cancer

Protocol Number: TQB2450-III-04

Version: 3.0 (6/6/2022)

PI: Dr. Ying Cheng

I have read and confirmed the protocol (Protocol Number: TQB2450-III-04; Version: 3.0; Version Date: 6/6/2022). I will conscientiously perform my duties according to the current GCP regulations in China and personally participate in or directly guide this clinical trial. I agree to perform my duties as a researcher in accordance with Chinese laws, Helsinki Declaration, China GCP, and this protocol.

Principal Investigator: Ying Cheng

Print/Type Name

Signed: ____________________ **Date:** ______________________

Coordinating Study Center Signature Page

Protocol Title: A Phase III Randomized, Double-blind, Parallel controlled, Multi-Center Trial of TQB2450 or Placebo plus Anlotinib and Etoposide/Carboplatin (EC) versus EC as First-line Therapy in Extensive-stage Small Cell Lung Cancer

Protocol Number: TQB2450-III-04

Version: 3.0 (6/6/2022)

Center: XXX

I have read and confirmed the protocol (Protocol Number: TQB2450-III-04; Version: 3.0; Version Date: 6/6/2022). I will conscientiously perform my duties according to the current GCP regulations in China and personally participate in or directly guide this clinical trial. I agree to perform my duties as a researcher in accordance with Chinese laws, Helsinki Declaration, China GCP, and this protocol.

Authorized Medical Expert: _________________

Print/Type Name

Signed: ___________________ **Date:** _____________________

Statistician Signature Page

Protocol Title: A Phase III Randomized, Double-blind, Parallel controlled, Multi-Center Trial of TQB2450 or Placebo plus Anlotinib and Etoposide/Carboplatin (EC) versus EC as First-line Therapy in Extensive-stage Small Cell Lung Cancer

Protocol Number: TQB2450-III-04

Version: 3.0 (6/6/2022)

Statistician: Department of Biostatistics, School of Public Health, Nanjing Medical University

I have read and confirmed the protocol (Protocol Number: TQB2450-III-04; Version: 3.0; Version Date: 6/6/2022). I will conscientiously perform my duties according to the current GCP regulations in China and personally participate in or directly guide this clinical trial. I agree to perform my duties as the statistician in accordance with Chinese laws, Helsinki Declaration, China GCP, and this protocol.

Authorized Statistician: Hao Yu________

Print/Type Name

Signed: ___________________ **Date:** _____________________

Version History/ Revision History

Version number	Version date	Brief rationale and description of changes
1.0	14Aug2019	Original version
2.0	18Oct2021	(1) Clarifying that the primary endpoint of PFS is assessed by IRC and the secondary endpoint of PFS is assessed by investigators;<br>(2) Myocardial damage markers, adrenocortical function, echocardiography, and lung function tests will be added during the screening period and the study to ensure the safety of the subjects;<br>(3) Clarifying the conditions for emergency unblinding;<br>(4) Addition of TQB2450 of 600 mg;<br>(5) Clarifying the maximum dose of carboplatin of 750 mg;<br>(6) Revised SAE reporting process;<br>(7) The correction of type I error in the interim analysis: considering that the O'Brien-Flemming method is used with a fixed sample size in each stage, it is not suitable for the adaptive design. Thus, the O 'Brien-Flemming method is revised to the Method Based on the Sum of P-values (MSP);<br>(8) Early the futility boundary: the futility boundary b1 of added P value (MSP method);<br>(9) Sample size estimation: sample size estimation by the PROC POWER process in SAS is replaced by computer simulation.
3.0	06Jun2022	(1) Addition of interim analysis for OS after 70% of the planned OS events;<br>(2) The test sequence will be adjusted: the OS test (OS3) of intervention arm 1 vs. intervention arm 2; then, the OS test (OS2) of intervention arm 2 vs. control group;

Contents

Protocol Synopsis....13
1. Introduction: Background Information and Scientific Rationale....17
1.1 Background Information....18
1.2 Overview of TQB2450 and Anlotinib....18
1.3 Overview of Preclinical Pharmacodynamics of TQB2450....19
1.4 Overview of Preclinical Pharmacokinetics of TQB2450....20
1.5 Overview of Preclinical Toxicology of TQB2450....20
1.6 Human Tolerance, Pharmacokinetics, and Other Clinical Studies....24
1.7 Pharmacological Study of Anlotinib....24
1.8 Toxicology of Anlotinib....24
1.9 Pharmacokinetics of Anlotinib....25
1.10 Safety and Tolerability of Anlotinib with Consecutive 2 Weeks and Stop 1 Week....29
1.11 Results of the Phase II Study of Anlotinib as Third-line or Later Therapy in SCLC....30
1.12 Current Status of First-line Therapy for SCLC....32
2. Objectives....33
3. Inclusion Criteria and Endpoints....34
3.1 Inclusion and Exclusion Criteria....34
3.2 Participant Withdrawal, Removal, or Termination....36
3.3 Criteria of Participant Removal....37
3.4 Criteria of Termination....37
3.5 Criteria of Continuous Treatment after PCI at the Maintenance Stage....37
3.6 Endpoints of the Study....38
4. Study Design....38
4.1 Sample Size Calculation....38
4.2 Interim Analysis....39
4.3 Randomization....40
4.4 Blind Design....40
4.5 Image Evaluation Approach....41
4.6 Handling of Randomization Errors....41

5. Study Drugs 41

5.1 Drug Information 42

5.2 Medication Management......42

5.3 Dosage Regimen 44

5.4 Disease Progression 50

6. Biological Samples 50

6.1 Serum Anti-TQB2450 Antibody (ADA) Assessment 50

6.2 Biomarker Assessment......50

7. Assessments 50

Screening......50

Treatment Period......52

End-of-treatment (EOT)......54

Post-treatment Follow-ups 54

8. Concomitant Medications 55

8.1 Prohibited Medications 55

8.2 Medications Given Cautiously......55

8.3 Permitted Concomitant Medications/Treatments......56

9. Efficacy Evaluation......56

10. Safety Analysis......57

10.1 AEs......57

10.2 AEs Grade 57

10.3 Record of AEs 57

10.4 Follow-up of AEs 60

10.5 Causality to Study Drugs 60

10.6 SAEs 61

10.7 Management of SAEs 61

10.8 Management of Common AEs......61

11. QoL 61

12. Data Management......62

12.1 Data Entry 62

12.2 Data Verification 62
12.3 Data Cleaning....62
12.4 Electronic Signature....62
12.5 Database Locking....63
12.6 Data Transfer....63
13. Statistical Analysis....63
13.1 Analysis Datasets 63
13.2 Statistical Analysis Plans....63
13.3 Study Population....67
13.4 Efficacy Analysis 68
13.5 Safety Analysis....69
13.6 Safety Analysis Software 71
14. Data Keeping 71
15. Sponsor/Investigator Responsibilities....71
15.1 Sponsor 71
15.2 Investigator....71
16. Ethical Considerations and Informed Consent....72
17. Study Summary....72
18. References....72

List of Abbreviations

Abbreviation	Full term
AEs	Adverse events
ALT	Alanine aminotransferase
AST	Aspartate aminotransferase
AUC	Area under the curve
ANC	Absolute neutrophil count
APTT	Activated partial thromboplastin time
ADA	Anti-TQB2450 antibody
ATC	Anatomical therapeutic chemical

BUN	Blood urea nitrogen
BCG	Bacille calmette-guerin
CCr	Creatinine clearance rate
Cr	Creatinine
CTCAE	Common Terminology Criteria for Adverse Events
CNS	Central nervous system
CT	Computed tomography
CEA	Carcinoembryonic antigen
CMH	Cochran-mantel-haenszel
CRA	Clinical research associates
DB	Direct bilirubin
DCR	Disease control rate
dMMR	Deficient mismatch repair
DLT	Dose-limiting toxicities
DOR	Duration of response
ECOG PS	Eastern Cooperative Oncology Group Performance Status
EC	Etoposide/Carboplatin
ES-SCLC	Extensive-stage small cell lung cancer
EOT	End-of-treatment
eCRF	Electronic case report form
EQ-5D	EuroQol five-dimensional
EDC	Electronic data capture
ECG	Electrocardiograms
EQ-VAS	EQ visual analog scale
FWER	Familywise error rate
FBG	Fasting blood glucose
FAS	Full analysis set
GCP	Good Clinical Practice
HBsAg	Hepatitis B virus surface antigen
HBV	Hepatitis B Virus

HCV	Hepatitis C Virus
Hb	Hemoglobin
HIV	Human immunodeficiency virus
HR	Hazard ratio
HRQOL	Health-related quality of life
IRC	Independent review committee
iRECIST	Immunotherapy Response Evaluation Criteria in Solid Tumors
INR	International normalization ratio
IUD	Intrauterine device
iDMC	Independent data monitoring committee
IV	Intravenous
irAEs	Immune-related adverse events
ITT	Intention-to-treat
LVEF	Left ventricular ejection fraction
MSI-H	High microsatellite instability
MTD	Maximum tolerated dose
MSP	Method based on sum of P-values
MRI	Magnetic resonance imaging
MedDRA	Medical dictionary for regulatory affairs
NOAEL	No-observed-adverse-effect level
NYHA	New York Heart Association
NSCLC	Non-small cell lung cancer
NSE	Neural specific enolase
NCI-CTCAE	National Cancer Institute Common Terminology Criteria for Adverse Events
ORR	Overall response rate
OS	Overall survival
PT	Prothrombin time
PCI	Prophylactic cranial irradiation
PFS	Progression-free survival

PLT	Platelets
PD	Progressive disease
PD-1	Programmed cell death protein 1
PD-L1	Programmed death ligand 1
PPS	Per-protocol Set
QTc	Corrected QT interval
QOL	Quality of life
RECIST	Response evaluation criteria in solid tumors
SAEs	Serious adverse events
SCLC	Small cell lung cancer
SD	Stable disease
SOP	Standard operating procedure
SS	Safety analysis set
SOC	System organ class
TBIL	Total bilirubin
TMB	Tumor mutational burden
TR	Thoracic radiotherapy
TKI	Tyrosine kinase inhibitor
TEAEs	Treatment-emergent AEs
ULN	Upper limit of normal value
VALG	Veterans Administration Lung Study Group
WBC	White blood cell count
WHO	World Health Organization

Protocol Synopsis

Protocol title	A Phase III Randomized, Double-blind, Parallel controlled, Multi-Center Trial of TQB2450 or Placebo plus Anlotinib and Etoposide/Carboplatin (EC) versus EC as First-line Therapy in Extensive-stage Small Cell Lung Cancer
Objectives	Primary objective<br>• To determine the progression-free survival (PFS) and overall survival (OS) of TQB2450 or placebo plus anlotinib and EC versus EC as first-line therapy in extensive-stage small cell lung cancer (ES-SCLC).
	Secondary objectives<br>• To compare the overall response rate (ORR), disease control rate (DCR), duration of response (DOR), 6-month PFS rate, 12-month PFS rate, 12-month OS rate, 18-month OS rate, and quality of life (QOL) across the three treatment arms.<br>• To compare safety and tolerability based on incidence and severity of adverse events (AEs) and serious adverse events (SAEs), as well as abnormal laboratory test indicators across the three treatment arms.<br>• To evaluate the immunogenicity of TQB2450 plus anlotinib and EC.<br>• To explore additional biomarkers related to efficacy, mechanisms of action, safety, and/or pathological mechanisms.
Endpoints	Primary Endpoints<br>• To evaluate PFS and OS, per Response evaluation criteria in solid tumors (RECIST) v1.1 assessed by the independent review committee (IRC).
	Secondary Endpoints<br>• To evaluate PFS per RECIST v1.1 and Immunotherapy RECIST (iRECIST), ORR, DCR, DOR, 6-month PFS rate, 12-month PFS rate, 12-month OS rate, 18-month OS rate, and QOL assessed by investigators.<br>• To evaluate safety and tolerability based on incidence and severity of AEs and SAEs, as well as abnormal laboratory test indicators.
Study population	ES-SCLC patients without previous systematic treatment
Sample size	738
Study design	Multi-center randomized, double-blind, parallel controlled, phase III trial.
Inclusion criteria	A subject can participate in the study only if all the following criteria are met:<br>(1) Pathologically confirmed diagnosis of ES-SCLC per Veterans Administration

	Lung Study Group (VALG) stage;<br>(2) No previous systemic treatment for ES-SCLC;<br>(3) Patients with prior chemoradiotherapy for limited-stage SCLC must have received radical therapy and have at least 6 months of no treatment interval between the end of chemotherapy, radiotherapy, or chemoradiotherapy and the diagnosis of ES-SCLC (calculated as the end time of the last chemotherapy cycle or the last radiotherapy);<br>(4) At least one measurable lesion based on RECIST v1.1; a previously irradiated lesion can be considered as a measurable lesion only if it has made definite progress after radiotherapy and is not the only one;<br>(5) Age 18-75 years; Eastern Cooperative Oncology Group performance status (ECOG PS) of 0-1; predicted life expectancy of ≥3 months.<br>(6) Adequate function of the important organs as evidenced by the following:<br>a) Hemanalysis (no blood transfusion within 14 days, no correction with hematopoietic stimulator drugs): hemoglobin (Hb) ≥90 g/L; absolute neutrophil count (ANC) $\geq 1.5\times10^9$/L; platelets (PLT) $\geq 75\times10^9$/L; white blood cell count (WBC) $\geq 3\times10^9$/L;<br>b) Biochemistry: alanine aminotransferase (ALT) and aspartate aminotransferase (AST) ≤2.5×upper limit of normal value (ULN) or ≤5×ULN for patients with liver metastases; total bilirubin (TBIL) ≤1.5×ULN or ≤3×ULN for patients with Gilbert syndrome; Creatinine (Cr) ≤1.5×ULN or creatinine clearance rate (CCr) ≥50 mL/min;<br>c) Coagulation function: activated partial thromboplastin time (APTT), international normalization ratio (INR), or prothrombin time (PT) ≤ 1.5×ULN;<br>d) Doppler ultrasound assessment: left ventricular ejection fraction (LVEF) ≥ 50%;<br>(7) Women of childbearing potential and men must agree to contraception for the duration of study treatment and 6 months after the last dose of study treatment, such as an intrauterine device (IUD), birth control pills, or condoms for women; women must have a negative serum pregnancy test within 7 days prior to the study grouping and be on-lactating;<br>(8) Be willing and able to provide written informed consent for the trial, and comply with all aspects of the protocol.
Exclusion criteria	Subjects who meet any of the following criteria are not eligible to enter the study:<br>(1) Previous therapy with antiangiogenic drugs (anlotinib, apatinib, bevacizumab, etc.) and related immunotherapy targeting PD-1, PD-L1, etc;<br>(2) Patients with brain metastasis and/or cancerous meningitis; (excluding asymptomatic patients or patients who are stable and have no imaging evidence of new/expanded central nervous system (CNS) metastasis at least 2 weeks after brain metastasis treatment and have stopped steroid or anticonvulsant treatment for at least

	14 days before the study; if active or new untreated and asymptomatic CNS metastasis are found at the screening stage, the subjects must receive radiotherapy or have no imaging evidence of new/expanded brain metastasis for at least 2 weeks without treatment);<br><br>(3) Patients with other malignancies within 5 years (other than cured cervical carcinoma in situ or skin basal cell carcinoma);<br><br>(4) Factors affecting oral medication (swallowing difficulty, post-gastrointestinal resection, chronic diarrhea, intestinal obstruction, et al.);<br><br>(5) Uncontrolled pleural effusion, pericardial effusion, or ascites requiring repeated drainage;<br><br>(6) Spinal cord compression without radically cured or alleviated by surgery and/or radiotherapy, or no clinical evidence of disease stabilization ≥1 week after treatment of previously diagnosed spinal cord compression prior to randomization;<br><br>(7) Invasion or unclear boundary between tumors and great vessels by imaging (computed tomography [CT]/ magnetic resonance imaging [MRI]);<br><br>(8) Evidence or history of a bleeding tendency within 2 months prior to the first dose, regardless of the severity; history of hemoptysis (defined as 1/2 teaspoon of bright red blood), or current non-healing wounds/ulceration/fracture within 2 weeks prior to the first dose;<br><br>(9) Previous unrelieved treatment-related toxicity (>grade 1) per the National Cancer Institute Common Terminology Criteria for Adverse Events (NCI-CTCAE) (excluding alopecia);<br><br>(10) Major surgical operation or obvious traumatic injury within 28 days prior to randomization;<br><br>(11) arterial and venous thrombosis (such as cerebrovascular accidents [including temporary ischemic attacks], deep vein thrombosis, and pulmonary embolism) within 6 months prior to randomization;<br><br>(12) A history of psychotropic substance abuse with the inability to quit, or dysphrenia;<br><br>(13) Any severe and/or uncontrolled disease:<br><br>a) Poor blood pressure control (systolic blood pressure ≥150 mmHg or diastolic blood pressure ≥100 mmHg);<br><br>b) class II or more of myocardial ischemia or myocardial infarction, arrhythmia (including men with corrected QT interval [QTc] ≥450 ms and women with QTc ≥470 ms); congestive heart failure in New York Heart Association (NYHA) functional class II-IV;<br><br>c) Active or uncontrolled severe infection (≥ grade 2);

	d) Liver cirrhosis, active hepatitis (Hepatitis B: HBsAg positive, and HBV DNA >ULN; Hepatitis C: HCV antibodies positive and HCV virus titers >ULN);<br>e) HIV-positive;<br>f) Poor diabetes control (fasting blood glucose [FBG] >10 mmol/L);<br>g) Urinary protein ≥++, and confirmed 24-hour urinary protein >1.0 g;<br>(14) Administration with prophylactic or attenuated vaccines within 4 weeks prior to the first dose;<br>(15) Severe hypersensitivity to other monoclonal antibodies;<br>(16) Active autoimmune diseases (e.g., autoimmune hepatitis, interstitial pneumonia, enteritis, vasculitis, nephritis, etc.; excluding asthma requiring medical intervention with bronchodilators) requiring systemic treatment (e.g., disease-modifying drugs, corticosteroids, or immunosuppressants) within 2 years prior to the study;<br>(17) Patients with immune deficiency or receiving immunosuppressive therapy of systemic glucocorticoid therapy or others (>10 mg/ day of prednisone or other equivalent hormones) within 2 weeks prior to the study; replacement therapy (e.g., thyroxine, insulin, or physical corticosteroids for adrenal or pituitary insufficiency) is not considered systemic therapy;<br>(18) Participation in other clinical trials within 4 weeks prior to the study;<br>(19) Concomitant diseases seriously endanger patients' safety or interfere with the completion of the study in the opinion of investigators.
Study procedures	Eligible subjects with ES-SCLC were randomly assigned to 3 treatment groups (intervention arm 1, intervention arm 2, and control arm). The treatment was divided into two stages: the induction stage and the maintenance stage.<br>**Stage 1: induction stage (21-day cycle for 4 cycles)**<br>The planned dose and scheme will follow the scheme below:<br>Intervention arm 1: 1200 mg intravenous TQB2450 on day 1; 12 mg oral anlotinib once daily on days 1-14; intravenous carboplatin at AUC 5 mg/mL/min on day 1 (the maximum dose was 750 mg); 100 mg/m^2 intravenous etoposide on days 1-3;<br>Intervention arm 2: 0 mg intravenous TQB2450 placebo on day 1; 12 mg oral anlotinib once daily on days 1-14; intravenous carboplatin at AUC 5 mg/mL/min on day 1 (the maximum dose was 750 mg); 100 mg/m^2 intravenous etoposide on days 1-3;<br>Control arm: 0 mg intravenous TQB2450 placebo on day 1; 0 mg oral anlotinib once daily on days 1-14; intravenous carboplatin at AUC 5 mg/mL/min on day 1 (the maximum dose was 750 mg); 100 mg/m^2 intravenous etoposide on days 1-3;<br>**Stage 1: maintenance stage (21-day cycle)**

	Participants with maintenance therapy will be administered until loss of clinical benefit, unacceptable toxicity, progressive disease (PD), or unsuitable continued medication as determined by the investigators. During maintenance therapy, patients were allowed to receive prophylactic cranial irradiation (PCI), but not radical thoracic radiotherapy (TR). The maintenance stage will be administered as follows:<br>Intervention arm 1: 1200 mg intravenous TQB2450 on day 1; 12 mg oral anlotinib once daily on days 1-14;<br>Intervention arm 2: 0 mg intravenous TQB2450 placebo on day 1; 12 mg oral anlotinib once daily on days 1-14;<br>Control arm: 0 mg intravenous TQB2450 placebo on day 1; 0 mg oral anlotinib placebo once daily on days 1-14;<br>**Dose reduction:**<br>Anlotinib: A maximum of two dose reductions of anlotinib (to 10/0 mg, 8/0 mg, and then discontinuation) was allowed according to drug-related AEs and cross-dose adjustment is not permitted. For subjects with a dose reduction to 10/0 or 8/0 mg, if subjects may experience disease progression per the investigators but still benefit from the increased dose after a period of medication, subjects could resume only one reduced dose when the safety profile is stable and cross-dose adjustment is not permitted.<br>TQB2450: No dose reduction will be considered.<br>Efficacy is assessed every 2 cycles. Subjects with disease control and tolerable AEs will continue treatment until loss of clinical benefit, unacceptable toxicity, PD, or unsuitable continued medication as determined by the investigators.<br>No other antitumor therapies will be considered during the study treatment.
Criteria for evaluation	**Safety:**<br>The AEs will be assessed by the NCI-CTCAE v5.0.<br>**Efficacy:**<br>Response to treatment will be mainly based on RECIST v1.1, and meanwhile iRECIST for further confirmation of the efficacy. Namely, patients diagnosed with PD according to RECIST v1.1 were further identified by iRECIST to determine whether to take further observation.
Safety monitoring	Hemanalysis, urinalysis and stool analysis, biochemistry, 12-lead electrocardiogram, thyroid function, coagulation function, etc.
Duration of trial	Aug 2019-Dec 2022

1. Introduction: Background Information and Scientific Rationale

1.1 Background Information

Anlotinib hydrochloride is a novel class 1.1 drug independently developed in China, which is the multitargeted antiangiogenic small molecule tyrosine kinase inhibitor (TKI). ALTER 0303 demonstrated that anlotinib as the third-line or later therapy could significantly prolong PFS and OS in patients with advanced non-small cell lung cancer (NSCLC) compared with a placebo. Anlotinib received its first approval by CFDA on 10 May 2018 based on the ALTER 0303 study. Results of the phase II study of anlotinib hydrochloride as the third-line or later therapy in small cell lung cancer (SCLC) were presented at the 2018 World Lung Cancer Congress. Significantly prolonged PFS and OS in patients with advanced SCLC were observed in anlotinib over placebo. Clinical studies conducted at the same time showed the encouraging efficacy of anlotinib hydrochloride in patients with advanced soft tissue sarcoma, medullary thyroid cancer, and differentiated thyroid cancer.

Usually, the programmed cell death protein 1 (PD-1) and its ligand, programmed death ligand 1 (PD-L1) costimulatory signals may play an important role in immunoregulation. In recent years, the PD-L1/PD-1 pathway has attracted wide attention due to its involvement in the tumor immune escape. The activation of the PD-1/PD-L1 pathway could lead to the establishment of an immunosuppressive microenvironment, allowing tumor cells to escape immune surveillance and killing. In contrast, blockade of the PD-1/PD-L1 pathway could reverse the tumor immune microenvironment and enhance the endogenous antitumor immune response. PD-L1 is highly expressed in various solid malignancies, including NSCLC, melanoma, renal cell cancer, prostate cancer, breast cancer, glioma, etc. PD-L1, whose expression profiles may vary in different tumors, could not only promote the development of tumor cells but also induce the apoptosis of T lymphocytes.

Nowadays, accumulating clinical studies on immunotherapy are performed. Mechanically, vascular anomalies could promote tissue hypoxia and enhance lactic acid, thus triggering the immunosuppression function and inhibiting T cell activation. Anti-angiogenesis could enhance the infiltration of immune effector cells by inducing tumor vessel normalization and reducing immunosuppression. Taken together, the current clinical study of anlotinib plus TQB2450 and chemotherapy is conducted by Chia Tai Tianqing Pharmaceutical Group Co., Ltd.

1.2 Overview of TQB2450 and Anlotinib

TQB2450:

TQB2450 is a novel humanized monoclonal antibody against PD-L1. TQB2450 could prevent the binding of PD-L1 to PD-1 and B7-1, reactivate the T cells, and enhance the immune response, thus harboring the potential to treat various tumors.

Molecular formula: $C_{6444}H_{9968}N_{1692}O_{2002}S_{46}$

Molecular weight: 144,651 Da (no glycosylation)

Anlotinib:

Anlotinib hydrochloride is a hard capsule. The chemical structure of anlotinib is as follows:

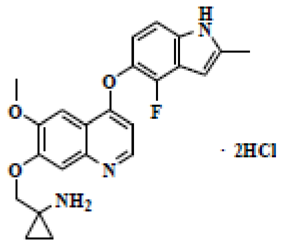

Molecular formula: $C_{23}H_{22}FN_3O_3 \cdot 2HCl$

Molecular weight: 480.36

1.3 Overview of Preclinical Pharmacodynamics of TQB2450

1.3.1 Results of In Vitro Pharmacodynamic Study

TQB2450 and Tecentriq® could bind to human PD-L1 and show equivalent binding activity with EC_{50} of 21.3 ng/mL and 27.6 ng/mL, respectively.

Both TQB2450 and Tecentriq® significantly induced IFN-γ secretion in MLR with DC cultured with CD4+ cells in a dose-dependent manner. Basically, equivalent induction to stimulation in TQB2450 (EC_{50}, 35.0±11.3 ng/mL) and Tecentriq® (EC_{50}, 26.6±1.4 ng/mL) indicated that the binding between TQB2450 and PD-L1 expressed on DC cells could inhibit PD-L1/PD-1 pathway and stimulate the secretion of IFN-γ in CD4+ T cells.

In the mixed reaction assay of DC and Tregs, IFN-γ was barely secreted, and TQB2450 showed no stimulatory activity on the IFN-γ. However, the opposite trend was observed in the Treg-free mixed reaction assay of DC and CD4+ CD25-T cells. If Treg, CD4+ CD25-T cells, and DC cells were mixed, Treg significantly inhibited IFN-γ secretion, while TQB2450 remarkably reversed the inhibitory activity of Treg. Overall, TQB2450 showed comparable activity to the control antibody Tecentriq®.

Neither TQB2450 nor Tecentriq® showed significant ADCC activity against MC-38/H-11 cells.

1.3.2 Results of In Vivo Pharmacodynamic Study

The antitumor rates of TQB2450 (1.5, 5, 15 mg/kg, IP, Q2D×11) on the subcutaneously transplanted tumor of MC-38/H-11 mice were 95.8%, 63.0%, and 91.7%, respectively. The results were calculated based on the anti-tumor rate (93.8%) of Tecentriq® (15 mg/kg, IP, Q2D×11) on MC-38/H-11 mice (all calculated per the median tumor volume). The therapeutic effect of TQB2450 on the subcutaneously transplanted tumor of MC-38/H-11 mice with colon cancer was comparable to that of Tecentriq®.

TQB2450 (1.5, 5, 15 mg/kg, IP, Q2D×11) significantly prolonged the survival of mice intraperitoneally inoculated with MC-38/H-11 cells. Thereinto, the median survival time was >98 days, and the survival rate to the end of the experiment (D98) was 70%, 80%, and 80%, respectively (P<0.01 compared with the human IgG 15 mg/kg group). The results are calculated based on the significantly prolonged survival, the median survival time of >98 days, and the survival rate of 100% of Tecentriq® (15 mg/kg, IP, Q2D×11) on mice with abdominal tumors. The efficacy (including survival time and survival rate) of TQB2450 on MC-38/H-11 mice with colon cancer was

comparable to those of Tecentriq®.

The antitumor rate of TQB2450 (1.5 mg/kg, IP, Q2D×4) on the subcutaneously transplanted tumor of human PD-1 transgenic MC-38/H-11 mice was 62% ($P<0.05$ compared with the human IgG 1.5 mg/kg group).

TQB2450 of 5-10 mg/kg (22 days, 3 times a week) and 10 mg/kg (22 days, once a week) by tail vein injection could inhibit the growth of transplanted tumors of A375 human melanoma in NGG mice in a dose-dependent manner. Thereinto, 10 mg/kg of TQB2450 at different administration frequencies (3 times a week and once a week) had the equivalent antitumor effect as the positive control of ATEZOLIZUMAB (3 times a week). The antitumor effect of TQB2450 may be related to the activation of human T cells and the increased release of IFN-γ in tumors. In vitro and in vivo pharmacodynamic studies confirmed that TQB2450 could prevent PD-L1 from binding to the PD-1 and B7.1 receptors on the T cells, and restore the activity of T cells, thus enhancing the immune response and playing the antitumor role.

1.4 Overview of Preclinical Pharmacokinetics of TQB2450

After single intravenous doses of TQB2450 at 1, 10, and 60 mg/kg, pharmacokinetic data in cynomolgus monkeys showed a linear dose-dependent relationship. Following multiple doses (10 mg/kg, once a week on weeks 1-4), there were no statistical differences in the peak time and half-life compared with a single dose. However, the peak concentration and drug exposure were higher than that of a single dose. Besides, the accumulation factor was 1.73±0.65 at the interval of 168 hr.

The radioactive distribution of TQB2450 was ranked by the area under the curve (AUC): serum, lung, liver, bone marrow, gonads, heart, adrenal gland, spleen, kidney, bladder, lymph nodes, small intestine, submandibular gland, fat, thymus, eyeball, large intestine, urine, pancreas, muscle, brain. Tissues and organs with abundant blood perfusion had greater radioactivity, such as the lung, liver, bone marrow, gonads, heart, etc. In contrast, organs with poor blood perfusion had less radioactivity, including fat, eyeballs, muscles, etc. Low radioactivity in the brain suggested that TQB2450 did not readily cross the blood-brain barrier.

TQB2450 was mainly excreted through urine and slightly through feces. The excretion rate of TQB2450 was slow.

1.5 Overview of Preclinical Toxicology of TQB2450

1.5.1 General Pharmacological Profile

Tissue cross-reactivity:

The degree of tissue cross-reactivity was assessed by a two-step method for immunohistochemistry. The results revealed that TQB2450 had specific tissue cross-reactivity with 18 normal tissues and 15 tissues of cynomolgus monkeys. The details were as follows:

Table 1.5.1 Tissue cross-reactivity

Species	Group	Cross-reactive tissue
Human	TQB2450	The pituitary gland, brain, cerebellum, lung, liver, skeletal muscle, heart, colon,

		small intestine, stomach, kidney, bladder, lymph nodes, spleen, thymus, bone marrow, blood cells, and placenta.
	Homotype negative control	Lungs, skeletal muscles, colon, bladder, kidney, stomach, heart, blood cells, and bone marrow.
Cynomolgus monkeys	TQB2450	The pituitary gland, lung, liver, muscle, heart, colon, small intestine, stomach, kidney, bladder, lymph nodes, spleen, thymus, bone marrow, and blood cells.
	Homotype negative control	Kidneys, blood cells, and bone marrow.

Effects on the cardiovascular system in awake monkeys (safety pharmacology study with jacket telemetry systems):

The cynomolgus monkeys were divided into 4 groups: control group, TQB2450 50 mg/kg group, TQB2450 100 mg/kg group, and TQB2450 200 mg/kg group. Each group had 5 male cynomolgus monkeys and 5 females. Each group was intravenously given control substances (placebo injection without TQB2450) or a corresponding concentration of TQB2450 at the volume of 20 mL/kg. The drugs were delivered once a week for 5 consecutive weeks, the infusion rate was ~2 mL/min. Then, the drug was suspended for 8 weeks. The day of first dose was the first day of the study. Lead II electrocardiogram (heart rate, QRS wave duration, PR interval, RR interval, P-wave travel time, QT interval (QTc), corrected QTc), blood pressure (systolic pressure, diastolic pressure, and mean blood pressure), and respiratory rate were determined at ~0-1, ~5-6, ~24-25, ~48-49, ~96-97 hours before and after the first dose, and at ~0-1 hour after the fifth dose, as well as 1 day before the autopsy following the recovery period.

Results showed that the electrocardiographic parameters, blood pressure, and respiratory rate of female and male monkeys in each group had no obvious abnormal alterations.

1.5.2 Acute Toxicity Test

Injection into cynomolgus monkeys:

Six cynomolgus monkeys were divided into 2 groups with 3 (both male and female) in each group. The two groups were given TQB2450 of 200 and 400 mg/kg, respectively, and the two were delivered single intravenous doses (10 mg/mL) of TQB2450 at the volume of 20 or 40 mL/kg, respectively. The day of first dose was the first day of the study.

The general condition of cynomolgus monkeys in each group was observed daily on days 1-14 after administration. Body mass was measured before infusion and on days 4, 9, and 14 of the study. The food intake was measured on days 2~3, 8~9, and 12~13 of the study. Temperature, lead II electrocardiogram, and blood pressure was measured at ~0-1 hour before and after the first dose and on day 14 of the study. Hematology and serum biochemical tests were performed on the 4th and 14th days of the study. All cynomolgus monkeys in each group were euthanized after anesthesia on day 15, and gross anatomy was observed.

Results indicated that general observation, body mass, food intake, temperature, electrocardiogram, blood pressure, hemanalysis, serum biochemistry, urinalysis, and gross anatomy of cynomolgus monkeys showed no obvious abnormalities following single intravenous doses of TQB2450 of 200 and 400 mg/kg. The maximum tolerated dose (MTD) was 400 mg/kg.

1.5.3 Long-term Toxicity Test

Toxicity and toxicokinetics of TQB2450 on cynomolgus monkey following intravenous injection for 4 weeks:

Cynomolgus monkeys were divided into a control group, TQB2450 50 mg/kg group, TQB2450 100 mg/kg group, and TQB2450 200 mg/kg group with 5 males and 5 females in each group. Each group was intravenously given control substances (placebo injection without TQB2450) or a corresponding concentration of TQB2450 at the volume of 20 mL/kg. The drugs were delivered once a week for 5 consecutive weeks, and the infusion rate was ~2 mL/min. Then, the drug was suspended for 8 weeks.

General condition, body mass, food intake, temperature, lead II electrocardiogram, blood pressure, respiratory rate, eye examination, hemanalysis, serum biochemistry, urinalysis, bone marrow examination, complement, circulating immune complex, lymphocyte subpopulation (CD3+, CD3+CD4+, CD3+CD8+, CD3+CD4+/CD3+CD8+, CD3-CD14+, CD3-CD16+, CD20+), hormones (T3, T4, TSH), cytokines (IFN-γ, TNF-α, IL-2, IL-6, IL-10), organ weight and coefficient, gross anatomy, and histopathological examination of each group had no obviously abnormal alterations.

TQB2450 had certain immunogenicity. The antibody-positive rates in 50, 100, and 200 mg/kg groups were 10%, 10%, and 0, respectively, and were detected within 6-8 weeks after discontinuance. The exposure of TQB2450 in cynomolgus monkeys increased proportionally with the increasing dose between 50 mg/kg and 200 mg/kg. Besides, certain accumulation was observed after multiple doses.

TQB2450 was injected intravenously into cynomolgus monkeys for 4 weeks and suspended for 4 weeks. The no-observed-adverse-effect level (NOAEL) was 200 mg/kg (AUC_{0-t} was 507909.7±139794.1 hr*µg/mL at this dose after 5 doses).

Table 1.5.3 Average toxicokinetic parameters of different doses of TQB2450 in cynomolgus monkeys

Time	Dose (mg/kg)		TQB2450		
			50	100	200
First dose	C_{max}	µg/mL	1112.9±166.7	2122.4±382.1	4120.8±476.4
	AUC_{0-t}	µg·h/mL	69524.6±10057.9	136371.1±20213.0	272611.4±45291.7
Last dose	C_{max}	µg/mL	1663.5±325.1	3343.6±810.9	6062.6±1245.5
	AUC_{0-t}	µg·h/mL	139614.2±27136.6	281160.2±97989.0	507909.7±139794.1

Accumulation factor	AR	—	2.0±0.4	2.0±0.5	1.9±0.4

1.5.4 Hemolysis Test and Vascular Stimulation Test

Hemolysis test

The hemolysis test consisted of a negative control group (0.9% sodium chloride injection), a positive control group (sterilization water for injection), and TQB2450 dose groups (0.1, 0.2, 0.3, 0.4, 0.5 mL/tube, 10 mg/mL, respectively). The mixture was composed of a set scale of 2% rabbit red blood cell suspension, TQB2450, 0.9% sodium chloride injection, and sterilization water for injection. The mixture was placed in the incubator at 37±0.5 °C and observed once at 0, 15, 30, 45, 60, 120, and 180 minutes, respectively. Make 3 parallel tubes in each group.

The results showed that after 3 hours at 37±0.5°C, hemolysis and coagulation were not observed in the negative control group, while hemolysis was presented in the positive control group. Besides, the supernatant was colorless and transparent in all TQB2450 sample groups. Red blood cells naturally sank and were redispersed following adequate oscillation. Hemolysis and coagulation were not observed in all TQB2450 sample groups.

Vascular stimulation test

The vascular stimulation test included the TQB2450 group with 4 male rabbits and 4 females. The highest planned concentration of 10 mg/mL was given by a single injection into the right auricular vein at the volume of 6 mL/kg. In parallel, the same volume of 0.9% sodium chloride injection as the control was administered through injection into the left auricular vein. The day of first dose was the first day of the study.

The general condition and injection site of rabbits were observed daily during the experiment. Four rabbits including 2 males and 2 females were euthanized and then dissected at ~72 hours and 16 days after injection, respectively. The injection site was observed by the naked eye, and the blood vessels and surrounding tissues of the injection site were examined by histopathology. A single injection of TQB2450 with a concentration of 10 mg/mL at a volume of 6 mL/kg was delivered in the ear vein of Japanese white rabbits, which had no irritation to the blood vessels and surrounding tissues of the injection site.

Comprehensive assessment:

TQB2450 did not significantly change the electrocardiographic parameters, blood pressure, and respiratory rate of male and female monkeys.

The general observation, body mass, food intake, temperature, electrocardiogram, blood pressure, hemanalysis, serum biochemistry, urinalysis, and gross anatomy showed no obvious abnormalities following single intravenous doses of TQB2450 of 200 and 400 mg/kg. The MTD was 400 mg/kg.

TQB2450 was intravenously injected for 4 weeks and suspended for 4 weeks. The NOAEL was 200 mg/kg (AUC0-t was 507909.7±139794.1 hr*µg/mL at this dose after 5 doses).

Hemolysis and coagulation were not observed in all TQB2450 groups. TQB2450 had no irritation to the blood vessels and surrounding tissues of the injection site.

1.6 Human Tolerance, Pharmacokinetics, and Other Clinical Studies

Patients with advanced malignancies without standard therapy were included in phase I clinical study of TQB2450. Exploratory studies with 6 dose groups including 1 mg/kg, 3 mg/kg, 10 mg/kg, 20 mg/kg, 30 mg/kg, and a fixed dose of 1200 mg were successively carried out according to the dose increment design. Patients were administered TQB2450 once every 3 weeks until progressive disease (PD) or intolerable toxicity.

Thirty-three patients with various advanced tumors were enrolled by February 2019. Preliminary results showed that the majority of the receptor occupation was more than 90% at day 21 of medication in the 3-30 mg/kg dose group. It suggested that TQB2450 could maintain saturation until the next dose. After intravenous injection of TQB2450, drug exposure and C_{max} showed good linearity in the 3-20 mg/kg dose group, and an estimated half-life was 320 hours at 1200 mg dose.

The patients in all dose groups had good tolerance. The adverse events (AEs) mainly were abnormities in various examinations, most of which were grade 1 or 2. No dose-limiting toxicity (DLT) was observed within 21 days after medication.

The short-term efficacy including objective remission and long-term tumor control was observed in patients with advanced Hodgkin's lymphoma and various solid tumors (including lung cancer, kidney cancer, etc.), which demonstrated the promising antitumor activity of TQB2450. The recommended regimen was TQB2450 of 1200 mg every 3 weeks according to the results of the Phase I study.

Clinical studies are performed based on the above results, including TQB2450 monotherapy in relapsed refractory Hodgkin's lymphoma and mediastinal large B-cell lymphoma, and TQB2450 plus chemotherapy as the first-line therapy in head and neck squamous cell carcinoma. In parallel, numerous exploratory studies of TQB2450 plus anlotinib in soft tissue sarcoma and hepatobiliary tract tumors have been initiated.

1.7 Pharmacological Study of Anlotinib

No significant effect on the general behavior and spontaneous activity of mice gavaged with a single dose of 2, 6, and 20 mg/kg after 24 hours was observed. The Beagle dogs were given different concentrations of anlotinib hydrochloride (0.3, 0.9, 3 mg/kg) through the duodenum after anesthesia. The abnormal systolic blood pressure, diastolic blood pressure, mean arterial pressure, and arrhythmia of anesthesia dogs were not observed in each dose group within 240 minutes after dosing. In parallel, anlotinib had no obvious effects on heart rate, PR, QRs, QTc interval of electrocardiograms (ECG), respiratory rate, and respiratory amplitude.

1.8 Toxicology of Anlotinib

1.8.1 Acute Toxicity Test

After ICR mice were gavaged with a single dose of anlotinib, the LD50 at days 1-14 was 1735.9 mg/kg with a 95% confidence limit of 1365.5-5474.6 mg/kg, and the LD50 at days 1-22 was 982.8 mg/kg with a 95% confidence limit of 657.28-1180.3 mg/kg. Possible target organs were the liver, gallbladder, small intestine (mainly duodenum), kidney, spleen, and testis. The Beagle dogs were given anlotinib as single intragastric administration and were then suspended and observed for 14

days. The MTD was 20 mg/kg and the minimum lethal dose was 67.5 mg/kg. The drug-related toxicities resembled those reported in similar drugs.

1.8.2 Long-term Toxicity Test

The SD rats were given anlotinib orally for 13 weeks and suspended for 6 weeks. The NOAEL was 0.25 mg/kg and the toxic dose was ≥1 mg/kg. The target organs were teeth, hepatobiliary system, duodenum, pancreas, adrenal gland, kidney, and blood system. The lesions of the hepatobiliary system, teeth, and blood system recovered significantly after discontinuance, while the lesions of other organs recovered completely.

The Beagle dogs were given anlotinib by intragastric administration for 13 weeks and suspended for 4 weeks. The main toxicities at the dose of 0.40 mg/kg ($AUC_{0\rightarrow 8h}$ 195 ng·h/mL) were gastrointestinal reactions, slight slowing of heart rate, and influence on liver and kidney functional indexes. The NOAEL was 0.12 mg/kg with the $AUC_{0\rightarrow 8h}$ of 51.7 ng·h/mL. After discontinuance for 4 weeks, all the above toxicities were recovered, and no delayed toxic reactions were observed.

1.8.3 Mutagenicity Test and Previous Literature

Anlotinib had no mutagenic effect on Salmonella typhimurium in Salmonella Typhimurium Histidine Reversion Test (Ames Test). Anlotinib did not induce chromosome structural aberration of Chinese hamster lung fibroblast cells in the chromosome aberration test. Anlotinib did not induce the increased micronucleus rate of mice marrow polychromatic erythrocytes in the micronucleus test.

1.8.4 Reproductive Toxicity Test and Previous Literature

The NOAEL for fetal development was <0.3 mg/kg when pregnant SD rats were intragastrically administered with anlotinib.

1.9 Pharmacokinetics of Anlotinib

1.9.1 Overview of Preclinical Pharmacokinetics in Animals

Pharmacokinetic parameters:

Results of plasma pharmacokinetic studies in rats and dogs indicated slower absorption of anlotinib hydrochloride in the gastrointestinal tract after oral administration. Bioavailability was ~34% in rats and 67% in dogs.

Plasma protein binding rate:

The binding rates between anlotinib and plasma protein in rats, dogs, and humans were 97%, 96%, and 93%, respectively. The rates did not change with the drug concentration of anlotinib.

Tissue distribution:

The drug concentration in all tissues was higher than that in blood at the same time, and there was no significant difference in the tissue distribution of anlotinib. The peak concentration in the lung was 184 (male) and 331 (female) times the plasma concentration at the same time. The peak concentrations in the spleen, adrenal gland, large intestine, small intestine, ovary, and kidney were 65-144 times the plasma concentration. The peak concentrations in the uterus, heart, liver, stomach,

bladder, bone marrow, and fat were 20-47 times the plasma concentration. The peak concentrations in skeletal muscle, pancreas, testis, and brain were 1.7-13 times plasma concentration.

Tissue distribution in tumor-bearing mice:

After 4 hours of oral administration, the concentration of anlotinib in the tissue of tumor-bearing nude mice was the highest. The AUC of anlotinib in all tissues was positively correlated with the dose, and the AUC in liver tissue was linearly correlated with the dose. The concentrations of anlotinib in the lung and liver were the highest (~10-14 times plasma AUC), followed by the kidney (~5.9-8.6 times plasma AUC). The AUC of anlotinib in the tumor was ~2.4-2.6 times of plasma AUC. The concentration in the colon was similar to that in plasma (~0.8-1.0 times plasma AUC).

Excretion:

The cumulative amount of anlotinib excreted (Cum. Ae) by urine (0-72 h), feces (0-72 h), and bile (0-24 h) was less than 5% of the injection dose (1.5 mg/kg). It suggested that metabolic transformation was the main pathway of elimination of anlotinib.

Metabolite:

Twenty-three metabolites were detected in rat bile; 16 metabolites were detected in rat urine; 12 metabolites were detected in feces; 8 metabolites were detected in plasma. Thereinto, M16, M21, and M23 were the 3 primary plasma metabolites. The cumulative excretion of M16 was the highest in urine and bile, while that of M18 was the highest in feces.

Metabolic enzyme activity:

Anlotinib could reversibly inhibit 7 CYP enzymes (8 substrates) in human liver microsomes, with IC_{50} values of >100 μM (CYP1A2), 4.96 μM (CYP2B6), 3.71 μM (CYP2C8), 1.56 μM (CYP2C9), 1.67 μM (CYP2C19), 24.7 and 18.5 μM (CYP2D6), 9.56 μM (CYP3A4-midazolam), and 2.09 μM (CYP3A4-testosterone), respectively.

1.9.2 Phase I Study of Pharmacokinetics in Humans

According to the protocol of the phase I study, all solid tumor subjects participating in the early tolerance study received blood drug concentration monitoring using liquid-mass combination technology (LC/MS/MS). The results were used to estimate the pharmacokinetic parameters of anlotinib hydrochloride.

Pharmacokinetics of a single dose:

Eligible subjects were given anlotinib once for a single-dose clinical pharmacokinetic study. Plasma concentrations of 19 subjects in 3 dose groups (10 mg, 16 mg, and 12 mg) were detected. The mean time curve of each dose group was shown on the left of **Figure 1.9.2-1**. The pharmacokinetic parameters estimated were shown in **Table 1.9.2-1**.

After single oral administration of anlotinib, anlotinib reached a high level of plasma concentration in 4-8 h and achieved a long half-life. At the dose of 10, 12, and 16 mg/person, the in vivo exposure of anlotinib (AUC_{0-168h}) was positively correlated with the dose, but the linear correlation was uncertain.

After a single oral administration, cumulative urinary excretion of anlotinib was ~<4% of the oral dose.

Table 1.9.2-1 pharmacokinetics of single administration of anlotinib in humans

Pharmacokinetic parameters	Mean±SD (RSD%)		
	10 mg/person (n=4)	12 mg/person (n=11)	16 mg/person (n=4)
C_{max} (ng/mL)	5.78±2.76 (47.7)	10.5±2.9（28.0）	15.8±3.2 (20.1)
T_{max} (h; p.o)	6.0± 4.4(73.3)	7.3±3.3（45.6）	11.0±8.9 (80.6)
$AUC_{0\text{-}t}$ (ng·h/mL)	385±175 (45.7)	875±240（27.5）	1290±384 (29.7)
$AUC_{0\text{-}\infty}$ (ng·h/mL)	562±328 (58.3)	1066±263（24.6）	1585±470 (29.6)
$t_{1/2}$ (h)	95.3±21.7 (23)	116±47（40.5）	97.9±14.8 (15.1)

Pharmacokinetics of multiple doses:

Eligible subjects received anlotinib once for a single-dose clinical pharmacokinetic study. Thereafter, the pharmacokinetic study with continuous administration was initiated following a ≥7-day wash-out period. Subjects received anlotinib on days 1-14 every 3 weeks and were monitored for at least 2 cycles. A total of 21 subjects in 3 dose groups (10 mg, 16 mg, and 12 mg) were given anlotinib and monitored the plasma concentration meanwhile. The mean time curve of each dose group was shown on the right of **Figure 1.9.2-1**. The pharmacokinetic parameters estimated were shown in **Table 1.9.2-2**.

After multiple doses, the plasma concentration of anlotinib increased along with the administration times due to the long elimination half-life of anlotinib in humans. The administration regimen with consecutive 2 weeks and stop 1 week was applied to control the plasma concentration of anlotinib following the administration. Anlotinib reached its maximum plasma concentration on day 14 following the administration regimen. The plasma concentration of anlotinib was almost <100 ng/mL at the dose of 10 and 12 mg/person/day.

Table 1.9.2-2 pharmacokinetics of anlotinib in 2 cycles of administration regimen

Pharmacokinetic parameters	Mean±SD (RSD%)		
	10 mg/person/day (n=3)	12 mg/person/day (n=15)	16 mg/person/day (n=3)
C_{max} (ng/mL)	65.2±28.9 (44.3)	61.6±16.3（27）	93.7±27.8 (30.0)
$AUC_{0\text{-}42}$ (ng·h/mL)	1550±864 (58)	1467±395（27）	2237±814 (36.4)

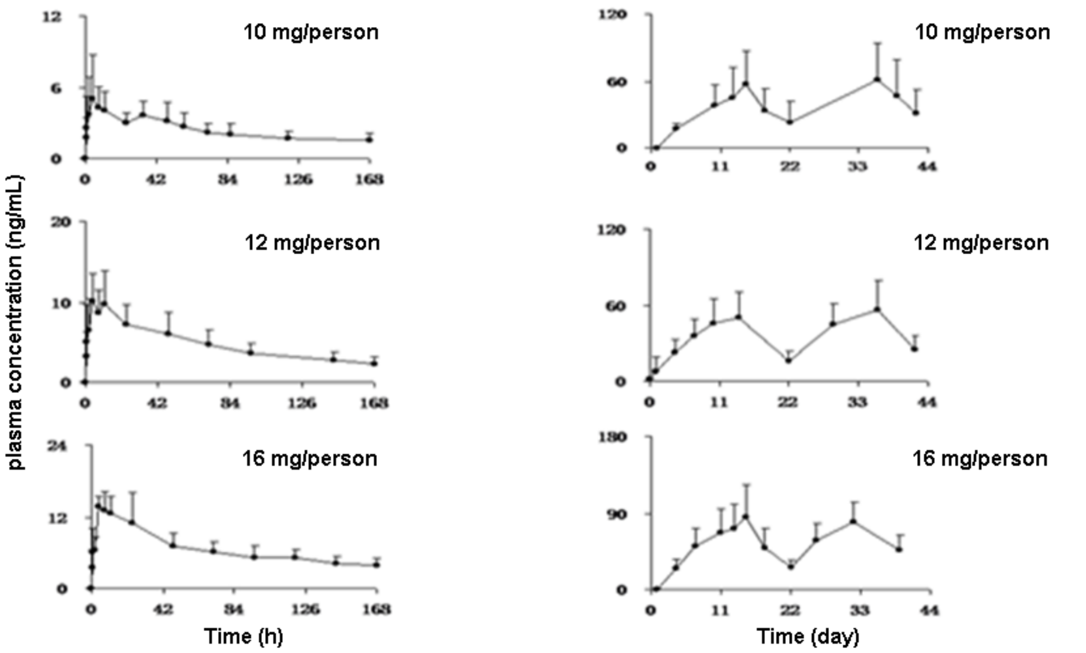

The time curve of a single dose The time curve of the administration regimen

Figure 1.9.2-1 The time curve of a single dose or administration regimen

Effect of food on the pharmacokinetics in healthy subjects:

The study was conducted through a randomized, two-period, self-crossover design. Twelve healthy subjects aged 18-40 were randomly and evenly divided into two groups: a fasting group with 3 males and 3 females and a high-fat diet group (3 males and 3 females). After fasting for 10 h, subjects were given 5 mg of anlotinib on an empty stomach or after a meal. Following a 28-day washout, subjects in the two groups were switched and the dose remained unchanged.

The pharmacokinetic parameters of administration in the fasting and postprandial state were similar, as detailed in **Figure 1.9.2-2** and **Table 1.9.2-3**. The results showed that, compared with fasting administration, the peak time of anlotinib in humans was extended following administration in the postprandial state, while the drug absorption was slightly reduced (~80% of fasting administration). Thus, it is recommended to take anlotinib in the fasting state in the subsequent clinical practice.

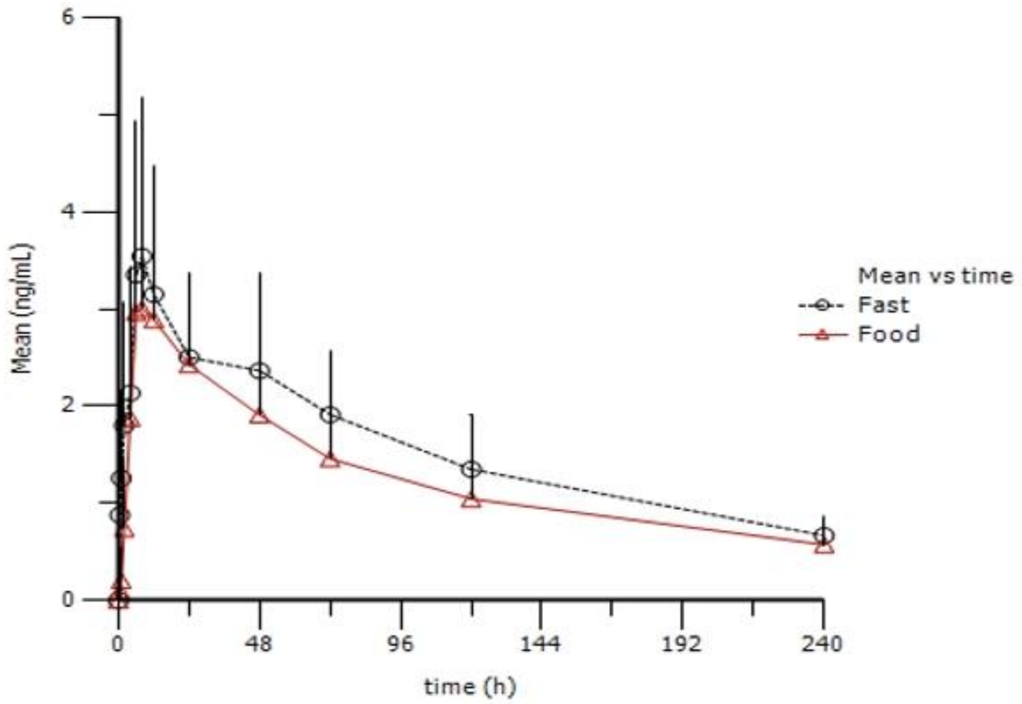

Figure 1.9.2-2 Effect of food on the pharmacokinetics in healthy subjects

Table 1.9.2-3 Effect of food on the pharmacokinetic parameters in healthy subjects

Pharmacokinetic parameters	High-fat diet group	Fasting group
C_{max} (ng/mL^{-1})	3.35±1.35	3.90±1.60
T_{max} (h)	10.5±6.7	9.3±5.1
AUC_{last}/AUC_{0-t} (ng·h·mL^{-1})	308±120	377±134
$AUC_{inf}/AUC_{0-\infty}$ (ng·h·mL^{-1})	392±145	486±158
MRT_{last}/MRT_{0-t} (h)	83.7±6.8	86.0±6.5
$MRT_{inf}/MRT_{0-\infty}$ (h)	153.2±43.7	161.7±41.3
$t_{1/2}$ (h)	107.2±27.4	113.2±30.3

1.10 Safety and Tolerability of Anlotinib with Consecutive 2 Weeks and Stop 1 Week

Safety profiles from 19 completed clinical trials involving 1576 patients exposed to anlotinib were summarized. These patients received anlotinib of the initial dose of 12 mg for 2 weeks, followed by 1-week discontinuation. Common AEs (≥20%) were hypertension, fatigue, hand-foot skin reaction, diarrhea, hyperlipidemia, proteinuria, hypothyroidism, oropharyngeal pain, cough, dysphonia, and various types of bleeding.

The safety profile of anlotinib in advanced NSCLC was primarily obtained from a multicenter, randomized, double-blind, placebo-controlled phase III trial (ALTER0303, n=437) and a multicenter, randomized, double-blind, placebo-controlled phase II trial (ALTER0302, n=117).

The ALTER0303 study enrolled patients with locally advanced/metastatic NSCLC and progression or intolerance following at least two types of systemic chemotherapy. Eligible patients with EGFR mutation or ALK-positive had progression or intolerance following targeted therapy. The Eastern Cooperative Oncology Group performance status (ECOG PS) of patients was 0-1. Patients with bleeding tendencies, poor blood pressure control, abnormal coagulation profile, and 24-hour urinary protein >1.0 g were excluded. A total of 294 patients received 12 mg oral anlotinib once daily on days 1-14 every 3 weeks, and 57.14% (168/294) of the subjects received treatment of ≥6 cycles. Twenty-five patients (8.5%) in the anlotinib group reduced the dose to 10 mg and three (1.0%) experienced two dose reductions (to 10 mg and then 8 mg). The main AEs leading to dose reduction were hand-foot skin reaction, hypertension, diarrhea, anorexia, oral mucositis, abnormal liver function, proteinuria, hyperlipidemia, and fatigue. The incidence of AEs with all grades in the anlotinib and placebo groups was 97.3% and 88.1%, respectively. The incidence of AEs with ≥grade 3 in the anlotinib and placebo groups was 47.3% and 18.2%, respectively.

Patients enrolled in the ALTER0302 study were similar to those in ALETR0303, but the ALTER0302 study included patients with an ECOG PS of 2 and unclear genetic status. Sixty patients received 12 mg oral anlotinib once daily on days 1-14 every 3 weeks. The incidence of AEs in the anlotinib and placebo groups was 86.7% (52/60) and 52.6% (30/57), respectively. The incidence of AEs with grade 3 or 4 in the anlotinib and placebo groups was 21.6% (13/60) and 5.3% (3/57), respectively. The AEs with an incidence of ≥5% in the anlotinib group included hypertension (53.3%), hand-foot skin reaction (25.0%), fatigue (18.3%), diarrhea (15.6%), oral mucositis (13.3%), oropharyngeal pain (11.7%), rash (10.0%), cough (8.3%), hoarseness (8.3%), hypothyroidism (6.7%), and hemoptysis (5.0%). Abnormal laboratory results of ≥5% included elevated blood thyroid-stimulating hormone (31.7%), hypertriglyceridemia (18.3%), hypercholesterolemia (16.7%), proteinuria (15.0%), elevated low-density lipoprotein (11.7%), elevated γ-glutamyl transpeptidase (11.7%), increased alanine aminotransferase (10.0%), and increased blood bilirubin (6.7%). The safety profiles of the phase II study were consistent with those of the phase III trial.

1.11 Results of the Phase II Study of Anlotinib as Third-line or Later Therapy in SCLC

The ALTER1202 study was a randomized, double-blind, placebo-controlled (2:1), multicenter study. Patients with pathologically confirmed advanced and recurrent SCLC following at least two prior chemotherapies were enrolled. A total of 120 subjects (82 in the anlotinib group and 38 in the control group) were enrolled in the study.

As of June 30, 2018, data from 120 SCLC patients who had previously received at least two chemotherapy regimens were analyzed. As of June 30, 2018, 25 patients in the anlotinib group and 1 patient in the placebo group continued the medication. The median PFS in the anlotinib group was 4.07 months (95%CI, 2.79-4.24), and that in the placebo group was 0.72 months (95%CI, 0.69-0.82), with a statistically significant difference (P<0.0001). Compared with the placebo, anlotinib extended the median PFS by 3.35 months and reduced the risk of tumor recurrence by 80.8% in patients with SCLC (hazard ratio [HR]=0.192; 95%CI, 0.117-0.315; P<0.0001).

The objective response rate (ORR) was 4.94% in the anlotinib group and 2.63% in the placebo group, respectively. The disease control rate (DCR) was 71.60% in the anlotinib group and 13.16% in the placebo group, with statistically significant differences between the two groups (P<0.0001).

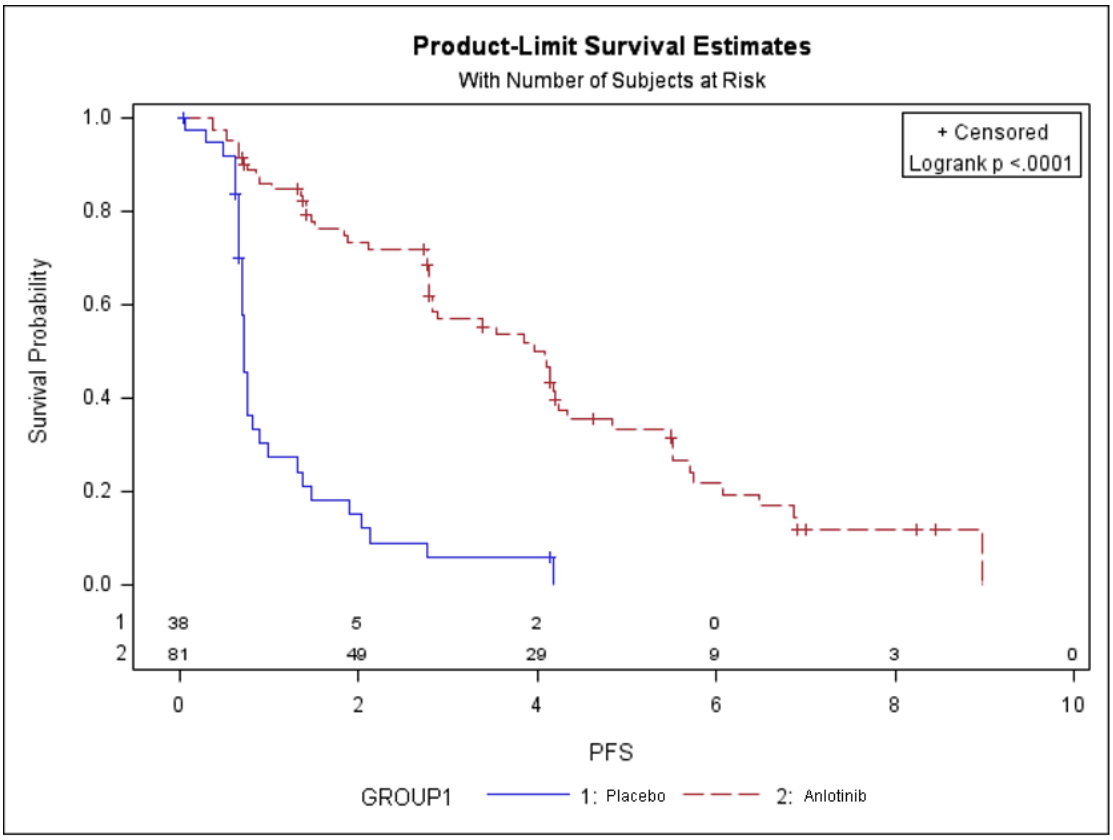

In the subgroup analysis with or without brain metastases at baseline, anlotinib extended the median PFS by 3.08 months in 30 SCLC patients with brain metastases over placebo (HR=0.146; 95%CI, 0.042-0.508; P=0.0032).

As of June 30, 2018, a total of 53 (44.54%) deaths were collected in 119 SCLC patients (1 case excluded from the full analysis set (FAS) due to misdiagnosis), including 21 (55.26%) in the placebo group and 32 (39.51%) in the anlotinib group. The median OS in the anlotinib group was 7.29 months (95%CI, 6.50-10.51), and that in the placebo group was 4.90 months (95%CI, 2.56-6.67), with a statistically significant difference (P=0.021). Compared with the placebo, anlotinib extended the OS by 2.39 months and reduced the risk of death by 47.2% in patients with SCLC (HR=0.528; 95%CI, 0.304-0.918; P=0.021). The 6-month survival rates in the anlotinib group and the placebo group were 70.3% and 33.2%, respectively.

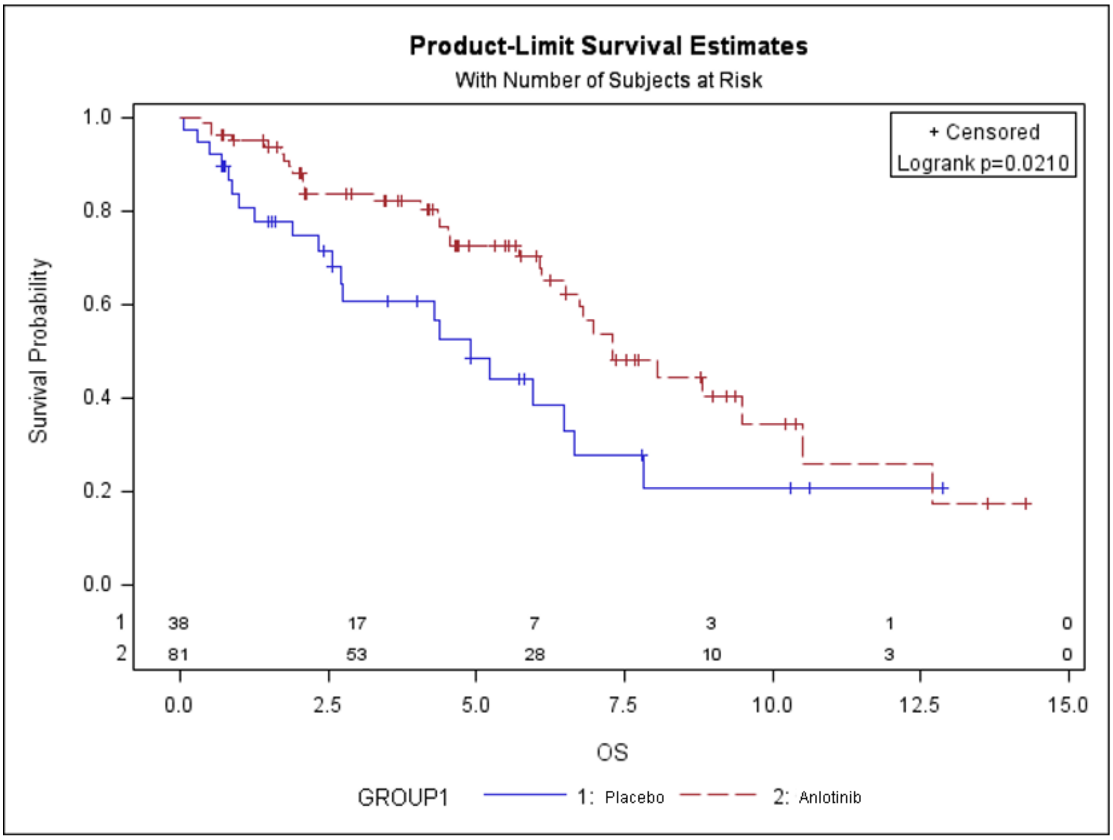

All 81 (100%) patients in the anlotinib group experienced AEs. Thirty-six (36/39, 92.31%) patients in the placebo group had AEs. There was a statistical difference between the two groups (P=0.0325).

In order to increase drug exposure in patients, dose reduction was considered when AEs occurred during the study. As of June 30, 2018, the dose of 4 (4.92%) patients in the anlotinib group was reduced to 10 mg, while not in the placebo group. The differences were not statistically significant between the two groups (P=0.3049). The reasons leading to the dose reduction in the anlotinib group were hand-foot skin reaction (n=3) and hypertension (n=1). The patients recovered from AEs at the reduced dose of anlotinib.

All AEs of any grade (≥10%) in the anlotinib group were presented as follows: hypertension (39.51%), decreased appetite (24.69%), fatigue (24.69%), hand-foot skin reaction (20.99%), elevated thyroid stimulating hormone (17.28%), hypertriglyceridemia (16.05%), decreased white blood cell count (16.05%), diarrhea (16.05%), increased alanine aminotransferase (16.05%), decreased lymphocyte count (16.05%), decreased body weight (14.81%), increased aspartate aminotransferase (14.81%), prolonged QTc (13.58%), hypothyroidism (13.58%), increased γ-glutamyl transferase (12.35%), proteinuria (12.35%) 12.35%), hypercholesterolemia (12.35%), decreased platelet count (11.11%), hyponatremia (11.11%), sinus tachycardia (11.11%).

1.12 Current Status of First-line Therapy for SCLC

Lung cancer is a malignant tumor with high morbidity and mortality worldwide. The incidence and mortality of lung cancer have increased rapidly in recent years. At present, the World Health Organization (WHO) has divided lung cancer into 2 major classes based on its biology, therapy, and prognosis: NSCLC and SCLC. SCLC accounts for 15-20% of lung cancer. SCLC is considered a

systemic disease since it is different from NSCLC in terms of tissue source, biological characteristics, response to treatment, and prognosis. SCLC is highly invasive and has a poor prognosis with a 5-year survival rate of <5%. The average survival of untreated patients was only 2-4 months.

Although most patients with SCLC missed the opportunity of radical therapy at first diagnosis, combination chemotherapy remained an effective treatment for extensive-stage small cell lung cancer (ES-SCLC), which could palliate symptoms and prolong survival. The response rate of ES-SCLC patients with first-line chemotherapy was 40%-70%, and the median survival was 7-11 months. Despite the high response rate of initial chemotherapy, most patients with complete response had disease progression within 3 months and poor long-term outcomes. EP regimen was the standard first-line therapy for ES-SCLC. In clinical practice, CBP was usually used to replace DDP to reduce gastrointestinal reactions, renal toxicity, and neurotoxicity, while CBP has a greater risk of inhibition of bone marrow hematopoietic function over DDP.

Although numerous combination chemotherapy regimens have been studied for first-line therapy of ES-SCLC, none replaced the standard therapy. On March 2019, the FDA approved atezolizumab plus chemotherapy as the first-line therapy of ES-SCLC based on the IMpower133 study. IMpower133 was a double-blind, randomized, placebo-controlled study of atezolizumab plus EC as the first-line therapy of ES-SCLC. ES-SCLC patients without prior treatment and mandatory detection of PD-L1were enrolled.

Patients were randomly assigned 1:1 to receive 4 cycles of intravenous carboplatin of AUC 5 mg/mL/min on day 1 and intravenous etoposide of 100 mg/m^2 on days 1-3 plus intravenous atezolizumab of 1200 mg on day 1 or placebo as initial therapy every 3 weeks. Thereafter, atezolizumab or placebo as maintenance therapy until disease progression, intolerant toxicity, or loss of clinical benefits. Patients could receive preventive brain irradiation (PCI) at the maintenance stage. However, radical thoracic radiotherapy (TR) was not allowed. The primary endpoints were PFS assessed by investigators and OS. The results of this study were announced at the World Lung Cancer Congress in 2018. The atezolizumab significantly prolonged the OS and mPFS of SCLC patients compared with the placebo (OS: 12.3 vs 10.3 months; mPFS: 5.2 vs 4.3 months).

2. Objectives

Primary objective:

- To determine the progression-free survival (PFS) and overall survival (OS) of TQB2450 or placebo plus anlotinib and EC versus EC as first-line therapy in ES-SCLC.

Secondary objectives:

- To compare the ORR, DCR, duration of response (DOR), 6-month PFS rate, 12-month PFS rate, 12-month OS rate, 18-month OS rate, and quality of life (QOL) across the three treatment arms.
- To compare safety and tolerability based on incidence and severity of AEs and serious adverse events (SAEs), as well as abnormal laboratory test indicators across the three treatment arms.
- To evaluate the immunogenicity of TQB2450 plus anlotinib and EC.

- To explore additional biomarkers related to efficacy, mechanisms of action, safety, and/or pathological mechanisms.

3. Inclusion Criteria and Endpoints

3.1 Inclusion and Exclusion Criteria

Inclusion criteria:

A subject can participate in the study only if all the following criteria are met:

(1) Pathologically confirmed diagnosis of ES-SCLC per Veterans Administration Lung Study Group (VALG) stage;

(2) No previous systemic treatment for ES-SCLC;

(3) Patients with prior chemoradiotherapy for limited-stage SCLC must have received radical therapy and have at least 6 months of no treatment interval between the end of chemotherapy, radiotherapy, or chemoradiotherapy and the diagnosis of ES-SCLC (calculated as the end time of the last chemotherapy cycle or the last radiotherapy);

(4) At least one measurable lesion based on RECIST v1.1; a previously irradiated lesion can be considered as a measurable lesion only if it has made definite progress after radiotherapy and is not the only one;

(5) Age 18-75 years; ECOG PS of 0-1; predicted life expectancy of ≥3 months;

(6) Adequate function of the important organs as evidenced by the following:

a) Hemanalysis (no blood transfusion within 14 days, no correction with hematopoietic stimulator drugs): hemoglobin (Hb) ≥90 g/L; absolute neutrophil count (ANC) ≥1.5×109/L; platelets (PLT) ≥75×109/L; white blood cell count (WBC) ≥3×109/L;

b) Biochemistry: alanine aminotransferase (ALT) and aspartate aminotransferase (AST) ≤2.5×upper limit of normal value (ULN) or ≤5×ULN for patients with liver metastases; total bilirubin (TBIL) ≤1.5×ULN or ≤3×ULN for patients with Gilbert syndrome; Creatinine (Cr) ≤1.5×ULN or creatinine clearance rate (CCr) ≥50 mL/min;

c) Coagulation function: activated partial thromboplastin time (APTT), international normalization ratio (INR), or prothrombin time (PT) ≤ 1.5×ULN;

d) Doppler ultrasound assessment: left ventricular ejection fraction (LVEF) ≥ 50%;

(7) Women of childbearing potential and men must agree to contraception for the duration of study treatment and 6 months after the last dose of study treatment, such as an intrauterine device (IUD), birth control pills, or condoms for women; women must have a negative serum pregnancy test within 7 days prior to the study grouping and be on-lactating;

(8) Be willing and able to provide written informed consent for the trial, and comply with all aspects of the protocol.

Exclusion criteria:

Subjects who meet any of the following criteria are not eligible to enter the study:

(1) Previous therapy with antiangiogenic drugs (anlotinib, apatinib, bevacizumab, etc.) and related immunotherapy targeting PD-1, PD-L1, etc;

(2) Patients with brain metastasis and/or cancerous meningitis; (excluding asymptomatic patients or patients who are stable and have no imaging evidence of new/expanded central nervous system (CNS) metastasis at least 2 weeks after brain metastasis treatment and have stopped steroid or anticonvulsant treatment for at least 14 days before the study; if active or new untreated and asymptomatic CNS metastasis are found at the screening stage, the subjects must receive radiotherapy or have no imaging evidence of new/expanded brain metastasis for at least 2 weeks without treatment);

(3) Patients with other malignancies within 5 years (other than cured cervical carcinoma in situ or skin basal cell carcinoma);

(4) Factors affecting oral medication (swallowing difficulty, post-gastrointestinal resection, chronic diarrhea, intestinal obstruction, et al.);

(5) Uncontrolled pleural effusion, pericardial effusion, or ascites requiring repeated drainage;

(6) Spinal cord compression without radically cured or alleviated by surgery and/or radiotherapy, or no clinical evidence of disease stabilization ≥1 week after treatment of previously diagnosed spinal cord compression prior to randomization;

(7) Invasion or unclear boundary between tumors and great vessels by imaging (computed tomography [CT]/ magnetic resonance imaging [MRI]);

(8) Evidence or history of a bleeding tendency within 2 months prior to the first dose, regardless of the severity; history of hemoptysis (defined as 1/2 teaspoon of bright red blood), or current non-healing wounds/ulceration/fracture within 2 weeks prior to the first dose;

(9) Previous unrelieved treatment-related toxicity (>grade 1) per the National Cancer Institute Common Terminology Criteria for Adverse Events (NCI-CTCAE) (excluding alopecia);

(10) Major surgical operation or obvious traumatic injury within 28 days prior to randomization;

(11) arterial and venous thrombosis (such as cerebrovascular accidents [including temporary ischemic attacks], deep vein thrombosis, and pulmonary embolism) within 6 months prior to randomization;

(12) A history of psychotropic substance abuse with the inability to quit, or dysphrenia;

(13) Any severe and/or uncontrolled disease:

a) Poor blood pressure control (systolic blood pressure ≥150 mmHg or diastolic blood pressure ≥100 mmHg);

b) class II or more of myocardial ischemia or myocardial infarction, arrhythmia (including men with corrected QT interval [QTc] ≥450 ms and women with QTc ≥470 ms); congestive heart failure in New York Heart Association (NYHA) functional class II-IV;

c) Active or uncontrolled severe infection (≥ grade 2);

d) Liver cirrhosis, active hepatitis (Hepatitis B: HBsAg positive, and HBV DNA >ULN; Hepatitis C: HCV antibodies positive and HCV virus titers >ULN);

e) HIV-positive;

f) Poor diabetes control (fasting blood glucose [FBG] >10 mmol/L);

g) Urinary protein ≥++, and confirmed 24-hour urinary protein >1.0 g;

(14) Administration with prophylactic or attenuated vaccines within 4 weeks prior to the first dose;

(15) Severe hypersensitivity to other monoclonal antibodies;

(16) Active autoimmune diseases (e.g., autoimmune hepatitis, interstitial pneumonia, enteritis, vasculitis, nephritis, etc.; excluding asthma requiring medical intervention with bronchodilators) requiring systemic treatment (e.g., disease-modifying drugs, corticosteroids, or immunosuppressants) within 2 years prior to the study;

(17) Patients with immune deficiency or receiving immunosuppressive therapy of systemic glucocorticoid therapy or others (>10 mg/ day of prednisone or other equivalent hormones) within 2 weeks prior to the study; replacement therapy (e.g., thyroxine, insulin, or physical corticosteroids for adrenal or pituitary insufficiency) is not considered systemic therapy;

(18) Participation in other clinical trials within 4 weeks prior to the study;

(19) Concomitant diseases seriously endanger patients' safety or interfere with the completion of the study in the opinion of investigators.

3.2 Participant Withdrawal, Removal, or Termination

3.2.1 Criteria for Participant Withdrawals

(1) The patients developed disease progression and could not benefit from further treatment determined by the investigators;

(2) The patients experienced the AEs without tolerance and alleviation;

(3) Subjects who experienced SAEs and were not suitable for further study;

(4) Patients who did not meet the eligibility criteria following the additional review;

(5) The serious protocol deviation or violation affected the evaluation of safety and efficacy;

(6) Patients who withdrew consent;

(7) Patients with failure to follow up for any reason.

3.2.2 Handling of Participant Withdrawals

Investigators should clearly collect the reason and time of withdrawal, conduct corresponding observation and evaluation of the withdrawn subjects, complete the evaluation content when withdrawing from the study as specified in the study plan, fill in the corresponding original records, and describe the reasons for withdrawal. Subjects who discontinue the study should continue to be

followed for AEs. If possible, these subjects should continue to follow up AEs until it recovers to the baseline level or the investigator believes that follow-up observation is unnecessary.

During the study period, all subjects who received the drug but did not complete the relevant study content of the protocol should have their last examination results transferred to the final result. Subjects who did not follow up as required and evidence of contact should be recorded in the corresponding original records and electronic data collections (EDCs; such as time and date of telephone contact, mailing receipt of registered mail, etc.) and properly preserved.

3.3 Criteria of Participant Removal

(1) Patients with chemotherapy, surgery, or other study drugs beyond the protocol during the trial;

(2) Patients who failed to meet the inclusion criteria were mistakenly included.

3.4 Criteria of Termination

(1) The researchers found severe safety problems based on the decisions of investigators;

(2) Withdrawal of the trial by the administrative department;

(3) Termination by the sponsors.

3.5 Criteria of Continuous Treatment after PCI at the Maintenance Stage

Brain metastases have been found in >50% of patients with SCLC. Studies showed that PCI could reduce the risks of brain metastases in SCLC patients. The randomized controlled EORTC study evaluated the differences in 286 ES-SCLC patients receiving or not PCI following response to initial chemotherapy. Compared with the control group, the symptoms of brain metastases were alleviated (14.6% vs. 40.4%) and the 1-year survival rate increased (27.1% vs. 13.3%) in the PCI group. In a randomized phase 3 study of evaluation of PCI in ES-SCLC conducted in Japan, although PCI reduced the risks of brain metastases (48% vs. 69%; P<0.0001), there was no overall survival benefit (11.6 months vs. 13.7 months; P=0.094). Therefore, PCI was recommended with caution for patients with ES-SCLC. The Chinese Society of Clinical Oncology (CSCO) Guidelines 2019 for the Diagnosis and Treatment of Primary Lung Cancer suggested that PCI should be started about 3 weeks after chemoradiotherapy. Brain-enhanced MRI should be performed before PCI, and PCI could be conducted without brain metastases. The dose of PCI was 25 Gy in 10 fractions over 2 weeks.

During maintenance therapy, patients are allowed to receive PCI, but not radical TR. The maintenance therapy after the PCI is conducted unless meeting the following criteria:

- Subjects within 28 days after completing required tests and assessments in the screening period still meet all inclusion criteria, but did not meet exclusion criteria (except the first-line therapy);
- Subjects do not receive antitumor treatments other than study therapy and PCI;
- PCI with drug-eluting stents for 2 weeks;
- The review and approval by the principal investigator.

If subjects continue to receive the maintenance therapy after PCI, subsequent follow-up should be

performed according to protocol until meeting the criteria for termination.

3.6 Endpoints of the Study

Primary endpoints:

- ✓ PFS and OS, per RECIST version 1.1 assessed by IRC.

Secondary endpoints:

- ✓ PFS per RECIST version 1.1 and iRECIST, ORR, DCR, DOR, 6-month PFS rate, 12-month PFS rate, 12-month OS rate, 18-month OS rate, and QOL assessed by investigators.
- ✓ Safety and tolerability based on incidence and severity of AEs and SAEs, as well as abnormal laboratory test indicators.

4. Study Design

This is a multicenter, randomized, double-blind, parallel-controlled phase III clinical trial.

4.1 Sample Size Calculation

4.1.1 Method of Sample Size Calculation

There are 3 arms in this study: Intervention arm 1 (TQB2450, Anlotinib, Carboplatin, and Etoposide); Intervention arm 2 (TQB2450 placebo, Anlotinib, Carboplatin, and Etoposide); Control arm (TQB2450 placebo, Anlotinib placebo, Carboplatin, and Etoposide). The primary efficacy endpoints of this trial are PFS and OS. In this study, a fixed-sequence test will be used for comparisons between treatment groups.

The safety analysis and re-estimation of sample size will be conducted by the Independent Data Monitoring Committee (iDMC). The re-estimation of sample size based on PFS is planned after 50% of the planned PFS events (174 cases). At the same time, a safety analysis will be performed. The interim analysis will be conducted by iDMC. One interim analysis for OS is planned for this study after 70% of the planned OS events. If expected efficacy is observed, early declaration of the study due to efficacy would be considered. The interim analysis will be conducted by iDMC.

The previous study showed that the median OS and PFS in the control arm were 10 and 4 months, respectively [5]. Patients were enrolled within a 12-month accrual period with an 18-month follow-up and were randomly assigned (1:1:1) to three groups. The power was 85% with a type I error rate of 0.050 and a dropout incidence of 10%. The type I error rate in the interim analysis for PFS will be controlled by the Method Based on the Sum of P-values (MSP). The initial sample size in this study will be estimated based on PFS using a computer simulation program.

4.1.2 Sample Size Estimation in the Intervention Arm 1

Assuming that the HR for PFS in the intervention arm 1 compared with the control arm is 0.6, the hazard rate in the intervention arm 1 is 0.1040, and that in the control arm is 0.1733 along with exponential distribution. Approximately 165 PFS events are expected if 174 patients are enrolled. A total of 194 patients are recruited and randomly assigned (1:1) to intervention arm 1 and control arm, considering an approximate dropout incidence of 10%. The interim analysis will be conducted when the planned PFS events are ~83. The final analysis will be performed after ~165 of the planned

PFS events.

Assuming that the HR for OS in intervention arm 1 compared with the control arm is 0.7, 284 OS events are expected with the type I error rate of 0.050 if 381 patients are enrolled. A total of 424 patients were recruited and randomly assigned (1:1) to intervention arm 1 and control arm, considering an approximate dropout incidence of 10%.

4.1.3 Sample Size Estimation in the Intervention Arm 2

Assuming that the HR for PFS in the intervention arm 2 compared with the control arm is 0.65, the hazard rate in the intervention arm 2 is 0.1126, and that in the control arm is 0.1733 along with exponential distribution. Approximately 231 PFS events are expected if 242 patients are enrolled. A total of 270 patients are recruited and randomly assigned (1:1) to intervention arm 2 and control arm, considering an approximate dropout incidence of 10%. The interim analysis will be conducted when the planned PFS events are ~116. The final analysis will be performed after ~231 of the planned PFS events.

Assuming that the HR for OS in intervention arm 2 compared with the control arm is 0.72, 331 OS events are expected with the type I error rate of 0.050 if 442 patients are enrolled. A total of 492 patients were recruited and randomly assigned (1:1) to intervention arm 2 and control arm, considering an approximate dropout incidence of 10%.

4.1.4 Determination of Total Sample Size

Considering the sample size estimation in sections 4.1.2 and 4.1.3, 738 subjects will be finally enrolled in this trial and be randomly assigned (1:1:1) to three groups. The interim analysis and final analysis for PFS are conducted for this trial after 174 and 347 cases of the planned PFS events, respectively. The interim analysis and final analysis for OS are performed for this trial following 348 and 497 cases of the planned OS events, respectively.

4.2 Interim Analysis

The interim analysis will be conducted by iDMC to assess the risk-benefit in the phase III trial. The iDMC, consisting of two independent oncologists and one independent statistician, is independent of the sponsors and investigators.

The analysis of safety and efficacy and re-estimation of sample size will be performed by iDMC in this trial. The re-estimation of sample size is mainly based on PFS and is planned after 50% of the planned PFS events (174 cases). At the same time, a safety analysis will be performed. If the planned sample size cannot achieve adequate power in the final analysis, the sample size will be increased. In contrast, if obtained power is adequate, the sample size will not be changed. The determinations for sample size are detailed in the iDMC guidelines. In parallel, efficacy analysis for OS is planned in this study after 70% of the planned OS events. If expected efficacy is observed, early declaration of the study due to efficacy would be considered. The interim analysis for OS and the final analysis for PFS will be carried out at the same time, whichever comes later.

Based on the analysis results, IDMC will provide a written recommendation to the sponsor on whether to carry out the trial or to continue the trial after modifying the protocol. The sponsor will make the appropriate decisions on the research project based on IDMC's recommendations, such as

termination of the trial due to safety or lack of efficacy.

4.3 Randomization

This is a multicenter, randomized, double-blind, parallel-controlled phase III clinical trial. A centralized randomized procedure will be used, with each center competing for enrollment. The centralized randomization procedure will use the eRand centralized randomization system. The independent statisticians upload the blinding code after the project's official launch. For patients with signing the informed consent form and were eligible for screening, the investigators log in to the randomization system and fill in the patient information (including participating center, initials, gender, date of birth, stratification factors for subjects, etc.). After confirming the basic information and stratification factors of subjects, randomization will be conducted by the investigator or authorized personnel to obtain the random number and the corresponding drug number. The appropriate research drugs are issued by the drug administrators of each participating center. The random number of each subject is a unique and permanent identifier. For those who are successfully randomized but do not receive the research drug, their drug and drug number shall be discarded and cannot be redistributed to others. The supervisor can log in to the system to verify the patient information and the drug use of each center, and send questions to the responsible individuals for any information in doubt. Subjects must start study treatment within 3 days following randomization. To ensure the equilibrium across three groups, stratification factors are designed as follows:

- ECOG PS: 0 or 1;
- Brain metastases (yes/no);
- Liver metastases (yes/no).

4.4 Blind Design

4.4.1 Double-blind Review Process

In this trial, a double-blind (double-simulation) design will be used in which the investigators, the research staff involved in the efficacy evaluation, the data managers, statisticians, and the subjects and their guardians are blinded to the grouping. The TQB2450 placebo and anlotinib hydrochloride placebo used in the control arm are manufactured, prepared, and tested by the sponsor to ensure the identification of the appearance, packaging, and smell.

4.4.2 Blinding Code

The independent statisticians irrelevant to the final analysis of the trial develop the blinding code and upload blinding code to the eRand centralized randomization system after the project's official launch. The blinding code will be saved in the eRand centralized randomization system during the trial.

4.4.3 Emergency Unblinding

Conditions for emergency unblinding

Emergency unblinding can be conducted when the subjects need to be rescued due to emergency events and the rescue operations depend on the known treatment the patient has received.

Process for emergency unblinding

An online emergency unblinding method will be adopted in this trial. If necessary, the primary investigator at each center can request an online emergency unblinding for a specific subject through the centralized randomization system: (1) The primary investigator at each center locates the record of the corresponding subject in the centralized randomization system; (2) Click the "emergency unblinding" and fill in the detailed reason for the emergency unblinding in the pop-up page; (3) After clicking "Confirm", the sponsor will receive an application email of emergency unblinding, and click "Agree" or "reject"; (4) If the sponsor clicks "Agree", the online emergency unblinding is successful, then the investigators will receive the emergency unblinding receipt, and the system will notify relevant personnel by email. Only the primary investigator of each center has access to emergency unblinding.

Emergency unblinding for pharmacovigilance

In the case of SUSAR, the independent pharmacovigil commissioners shall apply for the blinding code to the system manager. The system manager will directly send the blinding code to the pharmacovigil commissioners who shall be responsible for keeping and maintaining the blinding code.

The record of emergency unblinding

After successful emergency unblinding, the centralized randomization system will generate an emergency unblinding receipt. The investigators will sign the printed emergency unblinding receipt for confirmation, and the original copy will be kept in the center. The subsequent emergency unblinding shall be recorded in the corresponding original medical record/ electronic case report form (eCRF). The investigators fill in the emergency unblinding record and send the scanned copy to the sponsor for preservation.

Handling after emergency unblinding

Once the subject is unblinded, the subject with this number will withdraw from the trial and will not be replaced. The condition of the subject shall be followed up until the patients improve or reach a stable state.

4.5 Image Evaluation Approach

The primary endpoints of this study are PFS and OS. The PFS is evaluated in parallel by an independent image review committee.

4.6 Handling of Randomization Errors

If a subject is identified who does not meet the study inclusion/exclusion criteria, or if there is a randomization error due to procedural manipulation, the sponsor's representative and the investigator must discuss whether to continue or withdraw from the trial. Sponsors are required to ensure appropriate record-keeping of such decisions.

5. Study Drugs

5.1 Drug Information

(1) Anlotinib hydrochloride capsules: Chia Tai Tianqing Pharmaceutical Group Co., Ltd. production

Specifications: 12 mg/tablet, 10 mg/tablet, 8 mg/tablet;

(2) Anlotinib hydrochloride placebo capsules: Chia Tai Tianqing Pharmaceutical Group Co., Ltd. production

Specifications: 0 mg/tablet;

(3) TQB2450 injection: Chia Tai Tianqing Pharmaceutical Group Co., Ltd. production

Specifications: 100 mg/10 mL, 600 mg/20 mL;

(4) TQB2450 placebo injection: Chia Tai Tianqing Pharmaceutical Group Co., Ltd. production

Specifications: 0 mg/10 mL, 0 mg/20 mL;

(5) Chemotherapy: carboplatin injection: purchased and supplied by Chia Tai Tianqing Pharmaceutical Group Co., Ltd.

Specifications: 0.1 g;

(6) Chemotherapy: etoposide injection: purchased and supplied by Chia Tai Tianqing Pharmaceutical Group Co., Ltd.

Specifications: 5 mL: 0.1 g;

The batch number is subject to the drug test report and package.

5.2 Medication Management

5.2.1 Administration Schedule

Eligible patients with ES-SCLC are randomly assigned to intervention arm 1, intervention arm 2, or control arm. The treatment is divided into the induction stage and the maintenance stage.

Stage 1: induction stage (21-day cycle for 4 cycles)

Intervention arm 1: 1200 mg intravenous TQB2450 injection every 3 weeks; 12 mg oral anlotinib once daily on days 1-14; intravenous carboplatin at AUC 5 mg/mL/min on day 1 (the maximum dose was 750 mg); 100 mg/m^2 intravenous etoposide on days 1-3;

Intervention arm 2: 0 mg intravenous TQB2450 placebo injection every 3 weeks; 12 mg oral anlotinib once daily on days 1-14; intravenous carboplatin at AUC 5 mg/mL/min on day 1 (the maximum dose was 750 mg); 100 mg/m^2 intravenous etoposide on days 1-3;

Control arm: 0 mg intravenous TQB2450 placebo injection every 3 weeks; 0 mg oral anlotinib placebo once daily on days 1-14; intravenous carboplatin at AUC 5 mg/mL/min on day 1 (the maximum dose was 750 mg); 100 mg/m^2 intravenous etoposide on days 1-3;

Stage 2: maintenance stage (21-day cycle)

Participants with maintenance therapy will be administered until loss of clinical benefit,

unacceptable toxicity, PD, or unsuitable continued medication as determined by the investigators.

Intervention arm 1: 1200 mg intravenous TQB2450 injection every 3 weeks; 12 mg oral anlotinib once daily on days 1-14;

Intervention arm 2: 0 mg intravenous TQB2450 placebo injection every 3 weeks; 12 mg oral anlotinib once daily on days 1-14;

Control arm: 0 mg intravenous TQB2450 placebo injection every 3 weeks; 0 mg oral anlotinib placebo once daily on days 1-14;

During maintenance therapy, patients were allowed to receive PCI, but not radical TR.

Criteria for dose reduction:

Anlotinib/placebo: A maximum of two dose reductions of anlotinib (to 10/0 mg, 8/0 mg, and then discontinuation) are allowed according to drug-related AEs, but the cross-dose adjustment is not permitted. For subjects with a dose reduction to 10/0 or 8/0 mg, if subjects may experience disease progression per the investigators but still benefit from the increased dose after a period of medication, subjects could resume only one reduced dose when the safety profile is stable, but the cross-dose adjustment is not permitted.

TQB2450 injection: No dose reduction will be considered.

Efficacy is assessed every 2 cycles. Subjects with disease control and tolerable AEs will continue treatment until loss of clinical benefit, unacceptable toxicity, PD, or unsuitable continued medication as determined by the investigators.

No other antitumor therapies will be considered during the study treatment.

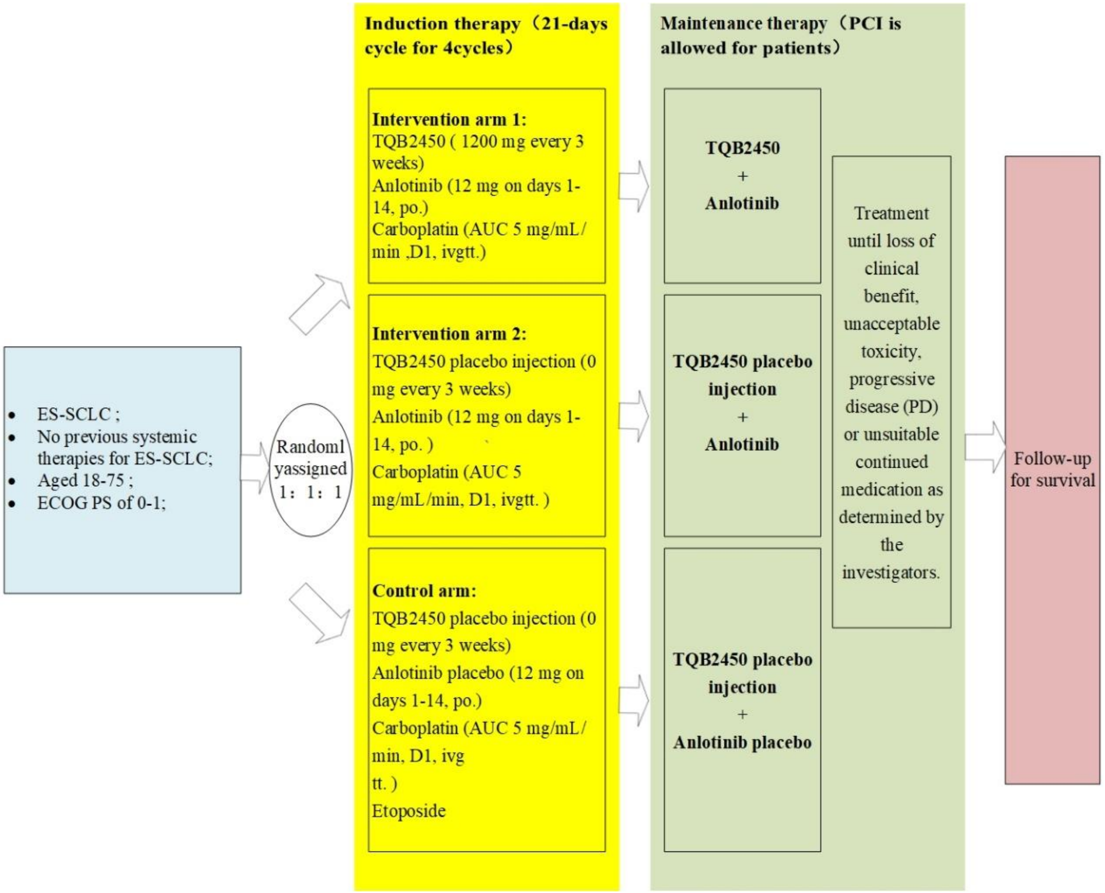

5.2.2 Drug Distribution, Use, and Recovery

According to the requirements of Good Clinical Practice (GCP), the research medication is governed by hospital custody.

Anlotinib for the test is released and recycled once every cycle. TQB2450 injection is used at the hospitals and is administrated, prepared, injected, and recycled by the major investigator's authorized personnel. Used or partially used drug containers, infusion bags, and syringes can be destroyed on-site according to guidelines and operating procedures established by the research center and local institutions. Unused drugs are returned to the sponsor unless the drug has serious safety problems that require immediate destruction in accordance with local laws and regulations.

Drug administration such as issuance and recycling requires complete records. The recovered drugs should be regularly monitored by the sponsor. Inspectors regularly check the usage and record of the drugs.

5.3 Dosage Regimen

5.3.1 Administration Method

TQB2450/placebo injection:

TQB2450 injection (1200/0 mg) will be diluted with normal saline to a final volume of 250 mL and infused for 60±10 minutes. Patients are administrated with TQB2450 once every 21 days. The infusion time starts with the beginning of the TQB2450 infusion and ends with the completion of

the TQB2450 infusion and normal saline flushing (20 mL normal saline flushing as a recommendation).

Observe the injection for particulate matter and discoloration before dilution. Do not use it if particulate matter or discoloration is confirmed.

Discard normal saline equal to the volume of TQB2450 injection that should be drawn from the 250 mL infusion bag containing 0.9% sodium chloride solution. Add the corresponding volume of TQB2450 injection to the intravenous (IV) infusion bags. Gently invert the solution without shaking to ensure adequate mixing.

The entire contents of the IV bag are intravenously delivered via a 0.2 μm or 0.22 μm in-line filter for 60±10 minutes. Flush immediately after the infusion. The start time and end time of medication will be recorded. If an infusion reaction occurs, appropriate intervention measures should be taken according to the protocol.

Notes: The volume of fluid is extracted in mL for infusion configuration. Different batches of drugs should not be mixed in a single infusion. Ensure that the TQB2450 infusion is transparent, without turbidity and precipitation.

TQB2450 does not contain preservatives and IV bags containing TQB2450 must be used immediately after dilution/mixing. If the TQB2450 dilution cannot be used immediately, the method of storage is as follows:

- ✓ The total storage time from opening the TQB2450 vial to the end of injection should not exceed 6 hours at room temperature;
- ✓ The total storage time should not exceed 24 hours in the refrigerator of 2 ℃-8 ℃ (36 °F-46 °F) without freezing and shaking;
- ✓ Mixing with other medications and intravenous injections should be avoided.

Anlotinib hydrochloride/placebo capsule:

Before breakfast, take anlotinib orally on an empty stomach, 1 tablet (12 mg)/once/day. Continue the drug administration for 2 weeks and stop for 1 week; thus, 3 weeks (21 days) for a treatment cycle. If there are no special circumstances, medication should be administered at a fixed time every day. If blood collection or safety tests are required, medication should be administered after blood collection. If there are missed medications and the interval from the next medication is <12 h, no medication is supplemented.

Carboplatin injection:

Dose of carboplatin (mg)=5 (AUC) (mg/mL/min)×[creatinine clearance within 7 days before the first dose (mL/min)+25]

Injection dispensing method and notes:

After calculating the dosage, the product is dissolved with 5% glucose injection at the concentration of 10 mg/mL and then added into the 250-500 mL of 5% glucose injection for intravenous infusion. The product must be used immediately within 8 hours after dilution. The intravenous infusion and

storage must avoid light.

Creatinine clearance Ccr (unit: mL/min) is calculated according to the Scr value of creatinine (calculation formula: Ccr=(140-age)×body weight (kg)/[72×Scr (mg/dl)] or Ccr=[(140-age)×body weight (kg)]/[0.818×Scr (μ mol/L)]. Attention should be paid to the unit of creatinine in the calculation of creatinine clearance. For women, the calculation result is ×0.85.

The U.S. Food and Drug Administration has recommended that physicians should consider setting an upper limit on the dose of carboplatin to reach the required exposure level (AUC) and avoid potential toxicity from overdoses. For patients with normal renal function, the maximum dose is based on GFR, with an estimated upper limit of 125 mL/min. AUC is 5 and the maximum dose of carboplatin is 750 mg.

Etoposide injection:

Dosage: 100 mg/m^2 on days 1-3;

Injection dispensing method and notes:

After calculating the dosage, the product is diluted with sodium chloride injection, and the concentration should not exceed 0.25 mg/mL. The intravenous infusion should not be less than 30 minutes.

Combined administration sequence:

Induction stage:

On the first day of each cycle, the anlotinib hydrochloride/placebo capsule is first administered orally on an empty stomach, and then TQB2450/placebo injection is intravenously given after at least 5 min, then carboplatin is intravenously given following at least 30 min, and etoposide is finally intravenously given after flushing.

On the second and third days of each cycle, the anlotinib hydrochloride/placebo capsule is first administered orally on an empty stomach, and then etoposide is finally intravenously given.

Maintenance stage:

On the first day of each cycle, anlotinib hydrochloride/placebo capsule is first administered orally on an empty stomach, and then TQB2450/placebo injection is intravenously given after at least 5 min.

Treatment cycle:

Treatment cycles are repeated every 21 days, with no other anticancer therapy during the medication. Patients with the disease control (CR+PR+SD) and who can tolerate adverse effects continue the medication until disease progression or intolerance.

5.3.2 Dose Delay and Adjustment Criteria

General principles

- ✓ The severity of AEs will be graded according to the National Cancer Institute Common Terminology Criteria for Adverse Events (NCI-CTCAE) v5.0 grading system. Given the trial

of the drug combination, the study drugs unrelated to AEs can be identified and the medication regimen can be maintained.

- ✓ When severe toxicity of different severity levels occurs simultaneously, adjustments should be performed according to the highest level observed;
- ✓ When toxic reactions occur, the investigators may refer to the following rules for delay or dose adjustment. Reasons for dose adjustments or delays, the supportive measures taken, and the outcomes will be documented in the patient's chart and recorded on the eCRF;
- ✓ If the investigators determine that the dose adjustment differs from the proposed adjustment rule, the appropriate adjustment will be performed by the investigators regarding clinical practice guidelines or previous clinical experience after consultation and communication with the sponsor; the relevant reasons will be recorded in the subject's medical record;
- ✓ If subjects require permanent discontinuation due to toxic reactions caused by chemotherapy, TQB2450 and anlotinib should be continued until disease progression or unacceptable toxicity (whichever occurs first);
- ✓ If subjects require permanent discontinuation due to immune-related toxicity of TQB2450, anlotinib and chemotherapy should be continued until disease progression or unacceptable toxicity (whichever occurs first).

Criteria for Dose Delay of TQB2450

If the medication is delayed due to AEs caused by TQB2450 injection and cannot be resumed over 12 weeks, TQB2450 injection treatment should be permanently terminated. The management of AEs induced by TQB2450 injection refers to Recommendations for the Management of immune-related Adverse Events (irAEs) Caused by Immune Checkpoint Inhibitor Therapy. TQB2450 dose delays are allowed, but no dose adjustment.

Criteria for Dose Delay and Adjustment of anlotinib

During the administration period (1-14 days) of anlotinib every cycle, if anlotinib-related AEs occur and the medication needs to be delayed, the maximum delay time should not exceed 5 days. If the medication cannot be continued for more than 5 days, the remaining anlotinib will not be administrated in this cycle. Until the start of the next cycle of medication, if the medication can continue to be delayed due to AEs, the maximum delay time shall not exceed 8 weeks.

Patients who remain unable to take anlotinib (including dose reduction) for more than 8 weeks need to permanently discontinue anlotinib (except for events caused by non-safety reasons). Dose delay and adjustment of anlotinib are allowed if necessary during the trial.

When anlotinib-related AEs occur during the trial, the dosages of anlotinib are allowed to be reduced (12/0 mg-10/0 mg-8/0 mg in turn). If the dose of 8/0 mg/d is not tolerated, then treatment should be terminated. If there is potential for disease progression and safety is manageable after the dose is reduced for a period of time, one dose level can be up-regulated to the initial dose from the next cycle.

Table. Dose levels of anlotinib

Dose levels	Usage	Specific amounts of drugs
1	12 mg oral, once a day	12 mg anlotinib hydrochloride capsules, 1 pill
2	10 mg oral, once a day	10 mg anlotinib hydrochloride capsules, 1 pill
3	8 mg oral, once a day	8 mg anlotinib hydrochloride capsules, 1 pill

When the non-bleeding AEs occur, dose adjustment can be performed according to the table below for general principles.

Table. The general principle of dose adjustment based on the grades of AEs

Grades of AEs (NCI-CTCAE v5.0)	Time of administration	Dose adjustment
Grade 3	Dose delay until recovery to grade 2	Continue administration after a reduced dose level; if the toxicity is not recovered after 8 weeks, the treatment should be terminated permanently.
Grade 4	Dose delay until recovery to <grade 2	Continue administration after a reduced dose level; if the toxicity is not recovered after 8 weeks, treatment should be terminated permanently; the investigator can stop the treatment permanently judging by the treatment.

When bleeding AEs occur, dose adjustment can be performed according to the table below.

Table. The principle of dose adjustment in the event of bleeding AEs

Bleeding AEs *	Dose adjustment
Grade 2	Suspended administration and active symptomatic treatment; restore to <grade 2 within 8 weeks, reduce a dose level; if necessary, treatment should be terminated permanently.
≥Grade 3	Permanent termination of treatment and emergency medical intervention.

* Bleeding AEs include hemoptysis, gastrointestinal bleeding, nosebleed, bronchial bleeding, gingival bleeding, gross hematuria, fecal occult blood, and cerebral hemorrhage.

Criteria for dose delay and dose adjustment of carboplatin and etoposide

Dose adjustment should be based on the severity of drug-related toxicity, which will be graded according to the NCI-CTCAE v5.0, and the potential clinical benefits in patients. A maximum of 2 dose reductions will be allowed; otherwise, treatment will be discontinued. Dose up-regulation is not considered in each patient.

When several AEs occur at the same time, the dose should be reduced according to the most severe

AE.

Table. The general principle of dose adjustment for carboplatin and etoposide

Dose levels	Carboplatin	Etoposide
1 initial dose	AUC 5	100 mg/m^2
2 reduced dose 1	AUC 4	75 mg/m^2
3 reduced dose 2	AUC 3	50 mg/m^2

Table. The principle of dose delay and adjustment for carboplatin and etoposide

AEs	Dose adjustment
ANC<1.5×10^9/L and/or platelet<100×10^9/L before the next dose	Dose delay until ANC>1.5×10^9/L; platelet> 100×10^9/L.
Grade 4 neutropenia with fever or infection, or duration ≥7 days; grade 3 neutropenia was present in the previous cycle, lasting until after day 21; grade 4 thrombocytopenia;	Intravenous infusion dose is lowered by one dose per day or the initial dose is maintained in the next dose, and G-CSF is given on day 4 (+2) of the treatment (24 hours after the carboplatin and etoposide treatment). If G-CSF does not control neutropenia, the dose should be reduced.
Grade 3/4 non-hematologic toxicity (excluding grade 3 nausea)	The intravenous infusion dose of carboplatin and etoposide should be reduced to one dose or the treatment should be discontinued.
Dose delay >6 weeks	Discontinuance.

Criteria for reapplying medication of carboplatin and etoposide:

(1) When drug-related AEs restore to ≤grade 1 or baseline, subjects can resume carboplatin and etoposide, excluding:

- when ANC >1.5×10^9/L, hemoglobin ≥90g/L and platelet >100×10^9/L;
- Treatment can be restarted in the event of grade 2 fatigue;
- Treatment can be restarted with grade 2 dermal toxicity and without grade 3 drug-related dermal AEs;
- Treatment can be restarted when drug-related pulmonary toxicity has returned to baseline;

(2) If the criteria for reapplying medication are not met, the next cycle of chemotherapy must be delayed. If the criteria are not met after a delayed dose of more than 6 weeks, one or both chemotherapies should be discontinued;

(3) If the next cycle of administration must be delayed due to carboplatin or etoposide-related toxicity, another chemotherapy should also be delayed.

5.4 Disease Progression

Since similar drugs of TQB2450 induced pseudo-progression in the clinical administration, the efficacy evaluation will be assessed by RECIST v1.1 and further confirmed by iRECIST criteria. That is, patients identified as having disease progression as per RECIST v1.1 will be confirmed by iRECIST to determine further treatment.

6. Biological Samples

6.1 Serum Anti-TQB2450 Antibody (ADA) Assessment

The scheduled intervals for immunogenicity monitoring will be based on the administration time of TQB2450. If the administration time of TQB2450 is delayed, blood sampling for immunogenicity will be delayed accordingly.

For subjects with positive ADA, the neutralization antibody will be detected.

Blood samples (5 mL) will be collected pre-dose (within -15 min) at cycles 1, 2, 5, 7, and day 90 (±7) after the last administration. Samples will be collected in separation gel procoagulant tubes for 30 min and then centrifuged at 3000 g for 10 min. The obtained serum will be stored for measuring the immunogenicity and blood concentration of TQB2450. Detailed procedure is listed in the SOP provided by the central laboratory.

Additional blood samples (5 mL) will be considered for subjects with unexpected irAEs to measure the immunogenicity and blood concentration of TQB2450. However, if the time of sampling is less than 24 hours from the latest collection, the additional sampling could be exempted.

6.2 Biomarker Assessment

Tumor tissues will be required for the biomarkers analysis, including PD-L1 expression, tumor mutation burden (TMB) detection, etc.

Fresh biopsy tissue samples within 1 month prior to enrollment are preferred for the biomarkers analysis. One or more percutaneous punctures are required for the fresh tissue sampling procedure. If fresh biopsy samples are not available, archived tissue samples are also allowed. Ten unstained pathological tissue sections (4 μm) are required for this study. If the sections can not be detected within 1 month, they should be embedded with paraffin. Detailed procedure is listed in the SOP provided by the central laboratory.

Blood samples (10 mL) will be collected before enrollment (within 7 days before administration) and at the end of the trial visit (±3 days) for the liquid biopsy-based biomarkers analysis, such as the ctDNA measurement of bTMB levels.

7. Assessments

Screening

- **Assessments within 14 days before study initiation:**

✓ Sign the informed consent form;

✓ Collection of medical history and baseline characteristics, including subjects' ID, gender, age,

mailing address, and telephone;

- ✓ Collection of medical history (if the initial diagnosis is ES-SCLC, the time of initial diagnosis should be recorded; if the initial diagnosis is limited-stage SCLC and developed into ES-SCLC later, two of time points should be recorded; TNM staging), and smoking history;
- ✓ Collection of previous anti-tumor therapies (e.g., the date and types of previous surgery; the start and end date of chemotherapy and radiotherapy, best response, treatment duration, and the date of the last dose);
- ✓ Echocardiography;
- ✓ Screening for infection, including human immunodeficiency virus (HIV) test, hepatitis B virus (HBV) test (if HBsAg is positive, quantitative serum HBV DNA levels will be detected), hepatitis C virus (HCV) antibody test (if HCV antibody is positive, quantitative HCV RNA levels will be detected);
- ✓ Assessment of liver function;
- ✓ Imaging examination (CT or MRI): PET will not be used as a routine method in the imaging assessment. Enhanced CT or MRI of the chest, abdomen, and pelvis is required for all subjects before administration. For subjects with bone metastases, a whole-body bone scan should be performed. All suspected lesions will be evaluated by imaging. During the screening period, a cranial plain scan plus enhanced MRI, and bone scan is required for all subjects (However, for subjects without bone metastases, whether the bone scan is performed will be decided by the investigator according to the symptoms);
- ✓ Collection of archived or fresh tumor tissue samples;
- ✓ Questionnaire for health-related quality of life (HRQOL);
- ✓ Concomitant medications and adverse events.

- **Assessments within 7 days before study initiation:**

- ✓ Systematic physical examination: ECOG PS, height, weight, vital signs, and physical examination;
- ✓ Serum pregnancy test: HCG testing (applicable to women of childbearing age);
- ✓ Serum carcinoembryonic antigen (CEA) and neural-specific enolase (NSE);
- ✓ 12-lead electrocardiogram;
- ✓ Hemanalysis, urinalysis and stool analysis (including occult blood), thyroid function (T3, T4, FT3, FT4, TSH), coagulation function (PT, APTT, TT, FBG, D-Dimer, INR);
- ✓ Liver function (TP, A, G, ALT, AST, LDH, ALP, TBil, DBil, IBil), renal function (BUN or UREA, Cr, UA), blood lipid profile (TC, TG, HDL, LDL), electrolyte (K^+, Na^+, CL^-, Ca^{2+}, Mg^{2+}, P), blood lipase, blood amylase, fasting blood glucose, etc.
- ✓ Biomarkers of myocardial injury;

✓ Adrenal cortex function;

✓ Blood samples for liquid biopsy-based biomarkers analysis;

✓ Concomitant medications and adverse events.

Treatment Period

Cycle 1

- Cycle 1 day 1 (C1D1):

  Vital signs and physical examination before administration;

  Concomitant medications, AEs, and medication allocation.

- C1D8:

  Vital signs and physical examination;

  12-lead electrocardiogram;

  Blood biochemistry and hemanalysis;

  Concomitant medications and AEs.

- C1D15:

  Vital signs and physical examination;

  12-lead electrocardiogram;

  Blood biochemistry and hemanalysis;

  Concomitant medications and AEs.

- C1D21:

  Vital signs and physical examination;

  12-lead electrocardiogram;

  Blood biochemistry, hemanalysis, urinalysis and stool tests (including occult blood), thyroid function, coagulation function, blood lipase, and amylase;

  Biomarkers of myocardial injury;

  Echocardiography;

  Adrenal cortex function;

  Concomitant medications, AEs, medication allocation, and recall.

Cycle 2

- Cycle 2 day 1 (C2D1):

  Vital signs and physical examination before administration;

Concomitant medications, AEs, and medication allocation.

- C2D8:

  Vital signs and physical examination;

  12-lead electrocardiogram;

  Blood biochemistry and hemanalysis;

  Concomitant medications and AEs.

- C2D15:

  Vital signs, physical examination;

  12-lead electrocardiogram;

  Blood biochemistry and hemanalysis;

  Concomitant medications and AEs.

- C2D21:

  Vital signs, ECOG PS, and physical examination;

  Imaging examination (CT or MRI), CEA, and NSE;

  12-lead electrocardiogram;

  Blood biochemistry, hemanalysis, urinalysis and stool tests (including occult blood), thyroid function, coagulation function, blood lipase, and amylase;

  Biomarkers of myocardial injury;

  Echocardiography;

  Adrenal cortex function;

  Questionnaire for HRQOL;

  Concomitant medications, AEs, medication allocation, and recall.

Cycle 3 and subsequent odd cycles (Vital signs and physical examination will be measured before administration on C3D1)

- Day 21 on every cycle:

  Vital signs and physical examination;

  12-lead electrocardiogram;

  Blood biochemistry, hemanalysis, urinalysis, and stool tests (including occult blood);

  Biomarkers of myocardial injury;

  Adrenal cortex function;

Concomitant medications, AEs, medication allocation, and recall.

Cycle 4 and subsequent even cycles (Vital signs and physical examination will be measured before administration on C4D1)

- Day 21 on every cycle:

Vital signs, ECOG PS, physical examination;

Imaging examination (CT or MRI), CEA, NSE;

12-lead electrocardiogram;

Blood biochemistry, hemanalysis, urinalysis and stool tests (including occult blood), thyroid function, coagulation function, blood lipase, and amylase;

Biomarkers of myocardial injury;

Echocardiography;

Adrenal cortex function;

Questionnaire for HRQOL;

Concomitant medications, AEs, medication allocation, and recall.

End-of-treatment (EOT)

- ✓ Medical history: if any PCI or palliative chest radiotherapy during the trial period (if so, record start and end time, treatment duration, etc.);
- ✓ Vital signs, ECOG PS, and physical examination;
- ✓ Imaging examination (CT or MRI), CEA, and NSE;
- ✓ 12-lead electrocardiogram;
- ✓ Blood biochemistry, hemanalysis, urinalysis and stool tests (including occult blood), thyroid function, coagulation function, blood lipase, and amylase;
- ✓ Biomarkers of myocardial injury;
- ✓ Echocardiography;
- ✓ Adrenal cortex function;
- ✓ Questionnaire for HRQOL;
- ✓ Blood samples for the liquid biopsy-based biomarkers analysis;
- ✓ Concomitant medications, AEs, and medication recall.

Post-treatment Follow-ups

All subjects will be followed up every 8 weeks (including telephone follow-up) to collect data on other antitumor therapy and disease status. The treatment regimens, cycles, and outcomes of any other treatment must be recorded during the trial. Post-treatment follow-up will be conducted until

death. Reasons and the date of death will be recorded for overall survival analysis.

8. Concomitant Medications

Any protocol-allowed medications taken by subjects for concomitant disease (except for study drugs) during the trial are concomitant medications and will be recorded on the medical record and eCRF. The following list of the recordkeeping time and requirements for concomitant medications is provided as guidance.

Recordkeeping time	Recordkeeping requirements
From the ICF signature to the first dose of the study drug	All medications or meaningful nondrug treatments: generic name, doses, the reason for treatment with this medication, start and stop date of this medication, or whether this medication was continued at study enrollment.
From the first dose of the study drug to the study withdrawal	All medications or meaningful nondrug treatments.
From the study withdrawal to 28 days after the last dose or starting other antitumor therapy (whichever occurs first)	All medications related to AE disposition.
Post-treatment phase (from 28 days after the last dose or starting other antitumor therapy to the end of the trial)	All medications related to anti-tumor therapy (in the OS follow-up section after study withdrawal).

8.1 Prohibited Medications

Subjects should not receive any approved medications with antitumor effects during the treatment period, such as chemotherapy, traditional Chinese medicine, and immunoregulator (thymosin, interferon, interleukin-2, purple dragon, lentinan, etc.).

Subjects should not receive live vaccines from 14 days before the first dosing of the study treatment to 60 days after the last dosing, including measles, mumps, rubella, chickenpox, yellow fever, seasonal influenza, H1N1 influenza, rabies, Bacille Calmette-Guerin vaccine (BCG), and typhoid vaccines.

8.2 Medications Given Cautiously

Medications/food that affect anlotinib will be used cautiously:

- Anticoagulant or clot-preventing medications, including but not limited to: Salicylic acid derivatives (e.g., aspirin); heparins (e.g., low molecular weight heparin, enoxaparin, dalteparin sodium, ardeparin sodium, etc.); Preventive anticoagulant drugs after cardiovascular events (e.g., clopidogrel and ticagrelor);
- Medications that interfere with hepatic cytochrome P450 enzymes, including but not limited to CYP3A inducers (carbamazepine, rifampicin); phenobarbital inhibitors (ketoconazole, itraconazole, erythromycin, and clarithromycin); CYP3A4 substrate (simvastatin, cyclosporine,

and piperidine); other medications metabolized by CYP3A4 (benzodiazepines, dihydropyridine calcium antagonist [calcium antagonism may be selected as appropriate for uncontrolled ACEI hypertension]; HMG-COA reductase inhibitor); CYP2C9 substrate (diclofenac, phenytoin sodium, piroxicam, S-warfarin, and tolbutamide); CYP2C19 substrate (diazepam, imipramine, lansoprazole, and S-mephenytoin);

- ➢ Citrus, star fruit, grapefruit, and grapefruit juice;
- ➢ Medications that prolong the cardiac QTc, including but not limited to:
  - ✓ Antimicrobials (clarithromycin, azithromycin, erythromycin, roxithromycin, metronidazole, moxifloxacin);
  - ✓ Antiarrhythmic drugs (quinidine, sotalol, amiodarone, disopyramide, procaine amide);
  - ✓ Antipsychotics (risperidone, fluphenazine, haloperidol, haloperidol, thioridazine, pimozide, olanzapine, clozapine);
  - ✓ Antifungal drugs (fluconazole, ketoconazole);
  - ✓ Antimalarial drugs (mefloquine, chloroquine);
  - ✓ Antidepressants (amitriptyline, imipramine, clomipramine, dosulepin, doxepin).

8.3 Permitted Concomitant Medications/Treatments

All treatments that the investigator considers no effects on study endpoints may be administered, such as unconventional therapies (e.g., herbal or acupuncture) and vitamin/mineral supplements.

Bisphosphonates are allowed for subjects with bone metastases during the trial. Palliative radiation therapy in a small area (<5% bone marrow region) will be allowed for subjects with uncontrolled pain of bone metastases after systemic therapy or topical analgesia.

To prevent an infusion reaction of TQB2450, H1 blockers (Diphenhydramine 50 mg, IV or equivalent) and acetaminophen (500-650 mg, oral or IV) may be premedicated 30 to 60 minutes prior to infusion of TQB2450. Systemic corticosteroid therapy for the management of infusion reactions or irAEs should be reduced gradually for at least 4 weeks. The reduced dose would not suppress immune system function (prednisone or equivalent ≤10 mg/day).

9. Efficacy Evaluation

Since similar drugs of TQB2450 induced pseudo-progression in the clinical administration, the efficacy evaluation will be assessed by RECIST v1.1 and further confirmed by iRECIST criteria. That is, patients identified as having disease progression as per RECIST v1.1 will be confirmed by iRECIST to determine further treatment.

Efficacy will be assessed every 2 cycles from cycle 1 day 1 until the radiologically confirmed disease progression. The frequency of radiographic assessments is independent of dose delays and/or dose interruptions. For subjects who discontinue the study drug for reasons other than disease progression, the tumor assessment will still be conducted every 2 cycles until starting a new antitumor therapy, disease progression, withdrawal of consent, or death, whichever occurs first.

The methods of radiographic assessment (CT or MRI) will be determined by the investigator. However, the assessment methods, machines, and technical parameters should be consistent throughout the study period. Contrast media is required for subjects without contraindications. For subjects who had received a radiographic assessment within 14 days before the first dose using the same procedure in the same center, the radiographic assessment could be used as the baseline data in this trial. The baseline tumor assessment should include enhanced CT or MRI of the chest, abdomen, and pelvis (except for subjects with an allergy to contrast media). During the screening period, a cranial plain scan plus enhanced MRI, and bone scan is required for all subjects. However, for subjects without bone metastases, whether the bone scan is performed will be decided by the investigator according to the symptoms. All suspected lesions will be evaluated by imaging. For subjects with bone metastases, the lesions will be monitored using a bone scan. If there is no exacerbation of clinical symptoms in these subjects, re-examination is not required at each visit for tumor assessment; otherwise, a timely re-examination is necessary. An unplanned tumor evaluation should be performed for subjects with suspected disease progression before the next planned evaluation. The radiographic assessment will only be conducted for lesions, but suspected lesions will also be evaluated by imaging.

10. Safety Analysis

10.1 AEs

AEs are defined as any untoward medical occurrence in a patient or clinical investigation subject administered a pharmaceutical product and which does not necessarily have to have a causal relationship with this treatment. Thus, AEs could be any unfavorable and unintended sign (including an abnormal laboratory finding), symptom, or disease temporarily associated with the use of a medical treatment or procedure regardless of whether it is considered related.

AEs also include the complications resulting from the protocol-defined interventions, such as complications caused by tissue biopsy or other invasive procedures; and the worsening of underlying disease (except for tumor progression) assessed by the investigator(s) during the reporting period of AEs.

Investigator(s) are required to record any AEs in subjects, including the events, AEs-related symptoms, time of occurrence, severity, duration, management, and outcomes.

10.2 AEs Grade

The severity grade of any AEs will be assessed according to the definitions in NCI-CTCAE v5.0.

10.3 Record of AEs

During the reporting period of AEs, the investigator(s) are required to record any AEs, including SAEs, in the CRF/eCRF. For the report of AEs, the investigator(s) are required to use the correctly standardized medical terminology rather than colloquialism and abbreviations. The start date, severity grade as per NCI-CTCAE v5.0, stop date, causality to study drugs, effects on the trial, concomitant therapy, and recovery will be recorded.

Diagnosis vs. signs and symptoms

A diagnosis (if known) should be recorded on the CRF/eCRF rather than individual signs and

symptoms (e.g., record only liver failure or hepatitis rather than jaundice, asterixis, and elevated transaminases). However, if a constellation of signs and/or symptoms cannot be medically characterized as a single diagnosis or syndrome at the time of reporting, each event should be recorded on the CRF/eCRF as AEs. If a diagnosis is subsequently established, all previously reported AEs based on signs and symptoms should be nullified and replaced by 1 AE report based on the single diagnosis.

AEs occurring secondary to other events

In general, AEs occurring secondary to other events (e.g., cascade events or clinical sequelae) should be identified by their primary cause, except for severe or serious secondary events. However, medically significant AEs occurring secondary to an initiating event that are separated in time should be recorded as independent events on the CRF/eCRF. All AEs should be recorded separately as primary or secondary events if it is unclear as to whether the events are associated.

Persistent, intermittent, or single AEs

Persistent AE extends continuously, without resolution between cycles/courses, such as the upper respiratory tract infection lasting 5 days. The event must only be reported once unless the grade becomes more severe. For severity grade, the highest severity grade should be recorded.

Intermittent AE occurs and resolves during a cycle/course of therapy, but without clinically significant outcomes, such as nausea and vomiting lasting for several days with the intermittent resolution, and persistent hypertension with intermittent resolution. The event must only be reported once. For severity grade, the highest severity grade should be recorded.

Single AE is one that occurs independently or only once during therapy, such as falls and one vomiting event during the trial. The event must only be reported once.

It should be noted that these above events are recurrent after clinically significant resolution and meanwhile have no course continuity with the former, each recurrence of an AE should be recorded separately on the CRF/eCRF.

Abnormal laboratory tests or vital signs

Laboratory test results will be recorded on the laboratory results pages of CRF. Not all abnormal laboratory tests and vital signs will be reported as AEs. Investigator(s) have the responsibility to review all laboratory findings and vital signs. Medical and scientific judgment should be exercised in deciding whether an isolated laboratory abnormality should be classified as an AE. Any abnormalities that meet one or more of the following conditions for clinical significance will be reported as AEs:

- Accompanied by clinical symptoms;
- Leading to a treatment change (e.g., dose modification, interruption, or discontinuation);
- Requiring medical intervention or the change of concomitant therapy (e.g., concomitant medication, new treatment, treatment interruption, discontinuation, or any other change);
- Have significant clinical significance as judged by the investigator.

If a clinically significant laboratory abnormality is a sign of a disease or syndrome (e.g., increased ALT/AST and bilirubin caused by hepatic dysfunction), only the diagnosis should be recorded on the CRF/eCRF (hepatic dysfunction). Otherwise, the abnormality should be recorded along with a descriptor indicating if the test result is above or below the normal range. If the abnormalities have corresponding standard clinical terms, the standard clinical terms should be recorded (e.g., an increase in blood potassium to 7.0 mmol/L should be recorded as hyperkalemia).

Death

For reporting death events, the death due to AE should be recorded as the single medical concept on the CRF/eCRF and reported as SAE. If the cause of death is unknown, "unexplained death" should be recorded on the CRF/eCRF, reported as SAE provisionally, and confirmed by further investigation. If the cause of death later becomes available, the "unexplained death" should be replaced by the established cause of death.

Pre-existing medical conditions

A preexisting medical condition should be recorded as an AE only if the frequency, severity, or character of the condition worsens during the study. When recording such events, it is important to convey the concept that the preexisting condition has changed by including applicable descriptors (e.g., more frequent headaches, and aggravated hypertension).

Hospitalization or prolongation of existing hospitalization

The following conditions leading to hospitalization or prolongation of existing hospitalization are not classified as SAEs:

- The hospitalization or prolongation of existing hospitalization required by protocol (e.g., administration and efficacy evaluation);
- An elective hospitalization for a pre-existing condition unrelated to the study indication. For example, the planned surgery or treatment before study or the scheduled surgery or treatment after enrollment. However, hospitalization for surgery or treatment due to disease worsening (surgery or treatment in advance) will be classified as SAEs.

Surgery

If the disease for surgery is definite, the disease should be recorded as AE rather than surgery (e.g., for subjects who underwent inguinal herniorrhaphy, AE is inguinal hernia rather than inguinal herniorrhaphy). Otherwise, the surgery should be recorded as AE (e.g., for subjects who underwent abbreviated laparotomy, abbreviated laparotomy is AE).

Pregnancy

If a female subject or a female partner(s) of a male subject becomes pregnant during the trial, the investigator(s) should be informed immediately. Investigator(s) are required to report to the sponsor within 24 hours of learning of its occurrence. Pregnant subjects should immediately stop using the study drug. The investigator should counsel the patient, and discuss the risks of continuing the pregnancy and the possible effects on the fetus. Pregnant subjects will be monitored until the end of pregnancy. All pregnancies within 30 days of the last administration will be reported to the

investigator(s).

Both induced and spontaneous abortions should be reported as SAEs. Any congenital anomalies/birth defects in infants born to female subjects or female partners of male subjects who had taken study drugs should be reported as SAEs.

Disease progression

If the observed progression is confirmed to be consistent with the expected pattern of primary tumor progression, it will not be considered an AE. The hospitalization due to this progression will also not be considered an SAE. If symptoms cannot be confirmed to be caused by tumor progression, or consistent with the expected pattern, the events will be considered an AE or an SAE.

10.4 Follow-up of AEs

Investigator(s) are required to follow up all AEs until any of the following occurs:

- AEs resolve to baseline;
- No further remission will be expected by the investigator(s);
- Death;
- Lost to follow-up;
- AEs that are not related to the study treatment confirmed by the investigator(s);
- Starting new antitumor treatment;
- End of clinical or safety data collection, or the database lock;
- No clinical or safety data are collected, or the database is eventually closed.

The outcomes of each AE (including the date of resolution and death) need to be recorded in the CRF/eCRF.

10.5 Causality to Study Drugs

Investigator(s) are required to assess the causality of AEs to study drugs, according to the following 5 criteria:

(1) Whether the administration time and the suspected AEs exhibit a reasonable relationship;

(2) Whether the suspected AEs fulfill the criteria for the typical reactions of the drug;

(3) Whether the suspected AEs can be explained by the effects of the combined drug, patient's clinical condition, or other therapies;

(4) Whether the suspected AEs disappear or are mitigated after drug discontinuation;

(5) Whether the same AEs recurred after repetitive administration of the study drugs.

	1	2	3	4	5
Definite	+	+	-	+	+

Probable	+	+	-	+	?
Possible	+	+	±	±	?
Unlikely	+	-	±	±	?
Unrelated	-	-	+	-	-

Note: +, Yes; -, No; ±, probably Yes or No; ?, unknown.

AEs will be calculated as the sum of definitely-related, probably-related, and possibly-related events.

10.6 SAEs

For this trial, AEs that meet one or more of the following criteria are classified as SAE: death, life-threatening events, in-patient hospitalization or prolongation of existing hospitalization, a persistent or severe disability or incapacity, congenital anomalies/birth defects, or any AEs that, based upon appropriate medical judgment, may jeopardize the subject and may require medical or surgical intervention to prevent one of the outcomes listed above. Pregnancy in patients or their spouses will be reported as SAEs to the sponsor or their representative.

Disease progression (including signs and symptoms of progression) under the trial will not be reported as an SAE, but death due to disease progression during the trial or safety reporting period will be reported as an SAE. Hospitalization due to signs and symptoms of disease progression will also not be reported as an SAE. During the trial or safety reporting period, events leading to death must be reported as SAE in dead subjects.

10.7 Management of SAEs

Any SAE, whether or not related to the study drug, occurring must provide the written report to the sponsor or their representative (TQB2450@cttq.com) within 24 hours of being aware of its occurrence, followed by the written follow-up report in detail. For death events, the investigator(s) have to provide the documents (e.g., autopsy and medical report) to the sponsor and ethics committee. The sponsor is required to immediately assess the severity of SAEs, their causality to the study drug, and whether it is an expected event. For suspected unexpected SAEs, the sponsor should quickly report to all participating investigators, clinical trial institutions, and the ethics committee, as well as the Drug Administration; Investigators are also required to inform these suspected unexpected SAEs of the ethics committee.

10.8 Management of Common AEs

The dose adjustment of anlotinib due to AEs is listed in section 5.3.2. The type and management of irAEs caused by TQB2450 are listed in the Appendix.

11. QoL

The QoL will be evaluated from various aspects through questionnaires. The exploratory objective of this trial is to evaluate the HRQOL, aiming to collect information on drug application as early as possible. The questionnaires for HRQOL will be completed at the scheduled time point.

To avoid being influenced by the investigator(s) or new conditions, the questionnaire should be

completed before any clinical examination and evaluation, as well as any new disease information and new treatment. The questionnaire will be stored as source documents.

Investigator(s) will review the questionnaire and evaluate the clinical signs. Any unobserved AEs in clinical evaluation will be recorded in the CRF.

The EuroQol five-dimensional (EQ-5D) descriptive system consists of a questionnaire and utility value conversion table. The questionnaire is reported for health conditions and EQ visual analog scale (EQ-VAS) score, while the utility value conversion table is reported for EQ-5D index values.

The questionnaire contains EQ-5D health state classifications and EQ-VAS. EQ-5D health state classification contains 5 dimensions, including mobility, self-care, usual activities, pain discomfort, and anxiety/depression. Each dimension contains 3 levels, including no problems, some problems, and extreme problems. EQ-VAS is a 20 cm vertical visual analog scale. 100 at the top indicates the best health you can imagine and 0 at the bottom indicates the worst imaginable health state.

12. Data Management

12.1 Data Entry

The data collected in this trial will be conducted using the electronic data capture (EDC) system. Investigator(s) or designated representative should complete the source document/eCRF timely, accurately, completely, and normatively. Data entry must be performed by authorized personnel who are qualified in EDC operation training.

12.2 Data Verification

Clinical research associates (CRA) have to conduct the source data verification to ensure the accuracy, completeness, consistency, and standardization of the data. Data administrators have to verify the data in the EDC system according to the verification protocol to ensure the accuracy, completeness, and standardization of the data. If any entries into the eCRF are incorrect or incomplete, any discrepancies will be noted in the EDC system by means of electronic data queries. The CRA and data administrators will ask the investigator or CRC to make appropriate corrections. The corrected data will again be reviewed for completeness and consistency.

12.3 Data Cleaning

Data cleaning includes EDC system automatic verification, manual verification, and SAS program cleaning. The investigator or CRC will confirm the questionable data and make appropriate corrections. The corrected data will again be reviewed by CRA and the data administrator for completeness and consistency. If the data queries are addressed, they will be closed, otherwise, they will be noted in the EDC system again until the queries solving.

12.4 Electronic Signature

The investigator is responsible for verifying the authenticity and accuracy of the data reported in the EDC system and making an electronic signature in EDC. The investigator must sign and date the Investigator’s Statement of electronic signature before any study-related procedures. It is required to declare that an electronic signature has the same legal effect as a written signature.

12.5 Database Locking

The database locking will be confirmed after the completion of data entry, data verification, electronic signature, medical verification, and data verification report. The data administrators will carry out the database locking after completing the listing procedure. The locked database will be not opened in principle.

12.6 Data Transfer

After database locking, the data set will be generated by the data administrators and be summited to the statistician. The eCRF of subjects will be archived in each center.

13. Statistical Analysis

13.1 Analysis Datasets

Intention-to-treat (ITT) Analysis Set: All randomized patients are included for efficacy analysis, according to the ITT principle. For the ITT population, patients will be considered in the treatment group as randomized.

ITT population is the primary analysis set and will be used for all efficacy and baseline characteristics analysis.

Per-protocol Set (PPS): All patients of the ITT population who receive the pre-defined minimum cycle of study treatment, had at least one imaging evaluation, without any major protocol deviation, with favorable compliance, and complete CRF.

PPS will be used for the per-protocol analysis of the primary efficacy variable and key secondary efficacy variables. The final PPS will be determined by the principal investigator, statisticians, and sponsors before the database lock.

Safety Analysis Set (SS): All randomized patients who receive at least one study treatment and have at least one safety assessment.

SS will be used for the safety analysis. For the SS population, patients will be considered in the treatment group as treated.

Baseline characteristics analysis will be performed for the ITT population. The analysis of the primary efficacy variable will be performed for the ITT and PPS population. The primary set for efficacy is the ITT. The analysis of laboratory data, AEs, and adverse reactions will be performed for the SS population.

13.2 Statistical Analysis Plans

13.2.1 General Approach

For quantitative variables, summary statistics will include the number of subjects, mean, standard deviation (SD), median, minimum, and maximum. The decimal places for the minimum or maximum will be the same as that recorded in the database. The decimal places for mean and median will report one more decimal place than the original data values in the database, and standard deviation will report two more decimal places than the original data values.

For categorical variables, frequency counts and percentage of patients will be presented. The percentage will be presented with two decimal places.

13.2.2 Size of a Test

(1) Type I error control in the multiple-group comparison

There are 3 arms in this trial:

Intervention arm 1: TQB2450, Anlotinib, Carboplatin, and Etoposide

Intervention arm 2: TQB2450 placebo, Anlotinib, Carboplatin, and Etoposide

Control arm: TQB2450 placebo, Anlotinib placebo, Carboplatin, and Etoposide

The primary efficacy endpoints of this trial are PFS and OS. In this trial, a fixed-sequence test will be used for comparisons between treatment groups:

a) The PFS between intervention arm 1 and the control arm (PFS1) will be first tested with the two-sided significance level of 0.05 (one-side, 0.025). If PFS1 is statistically significant, the OS between intervention arm 1 and the control arm (OS1) will be then tested.

b) If OS1 is statistically significant, the PFS between intervention arm 2 and the control arm (PFS2) will be tested.

c) If PFS2 is statistically significant, the OS between intervention arm 1 and intervention arm 2 (OS3) will be then tested.

d) If OS3 is statistically significant, the OS between intervention arm 2 and the control arm (OS2) will be tested.

The overall significance level is set as 0.05 at two-side (one-side, 0.025). Familywise error rate (FWER) will be controlled by the fixed order test when each hypothesis presupposes the rejection of all previous tests.

(2) Type I error control for the re-estimation of sample size based on PFS

The safety analysis, re-estimation of sample size, and efficacy analysis will be conducted by the iDMC. The re-estimation of sample size based on PFS is planned after 50% of the planned PFS events (174 cases). At the same time, a safety analysis will be performed. The interim analysis will be conducted by iDMC.

The non-blind re-estimation of sample size is a procedure with multiple tests, with a concern on data dependency in different stages, which would increase the inflation of the type I error rate. To address this problem, P values in two stages will be combined using the MSP.

According to the preliminary test, in the PFS interim analysis based on the re-estimation of sample size, the one-side significance level is set as α_1=0.005, and the futility boundary is set as b_1=0.45 in the first stage; the one-side significance level is set as α_2=0.2050 in the secondary stage. No early discontinuation of the study due to PFS efficacy would be considered.

- Either $P_1 \leq \alpha_1$ or $P_1 > \alpha_1$, once $P_1 \leq b_1$, the trial will continue to the second stage, without early discontinuation due to PFS efficacy;

- If $P_1>b_1$, the trial will terminate early for futility;
- If the trial could proceed into the second stage, $P_1+P_2\leq\alpha_2$ [that is, adjust P value=$\alpha_1+(P_1+P_2)(b_1-\alpha_1)-1/2\ (b_1^2-\alpha_1^2)\leq 0.025$], the between-group difference in PFS will be considered as statistically significant.

(3) Type I error control for interim efficacy analysis based on OS

One interim analysis for OS is planned for this trial after 70% of the planned OS events (348 cases). If expected efficacy is observed, early declaration of the trial due to efficacy would be considered. The interim analysis will be conducted by iDMC.

Type I error will be controlled by the O'Brien-Fleming spending function approach. According to the O'Brien-Fleming spending function approach, in the interim efficacy analysis based on OS, the one-side significance level is set as α_1=0.0074 in the interim analysis and α_2=0.0228 in the final analysis.

- In the interim analysis: $P_1\leq\alpha_1$, early declaration of the trial will be considered according to actual results;
- In the final analysis: $P_2\leq\alpha_2$, the between-group difference in OS will be considered statistically significant.

P_1=the one-side P value in the interim analysis; P_2=the one-side P value in the final analysis;

The tests sequence and type I error in the final analysis are shown in the following figure:

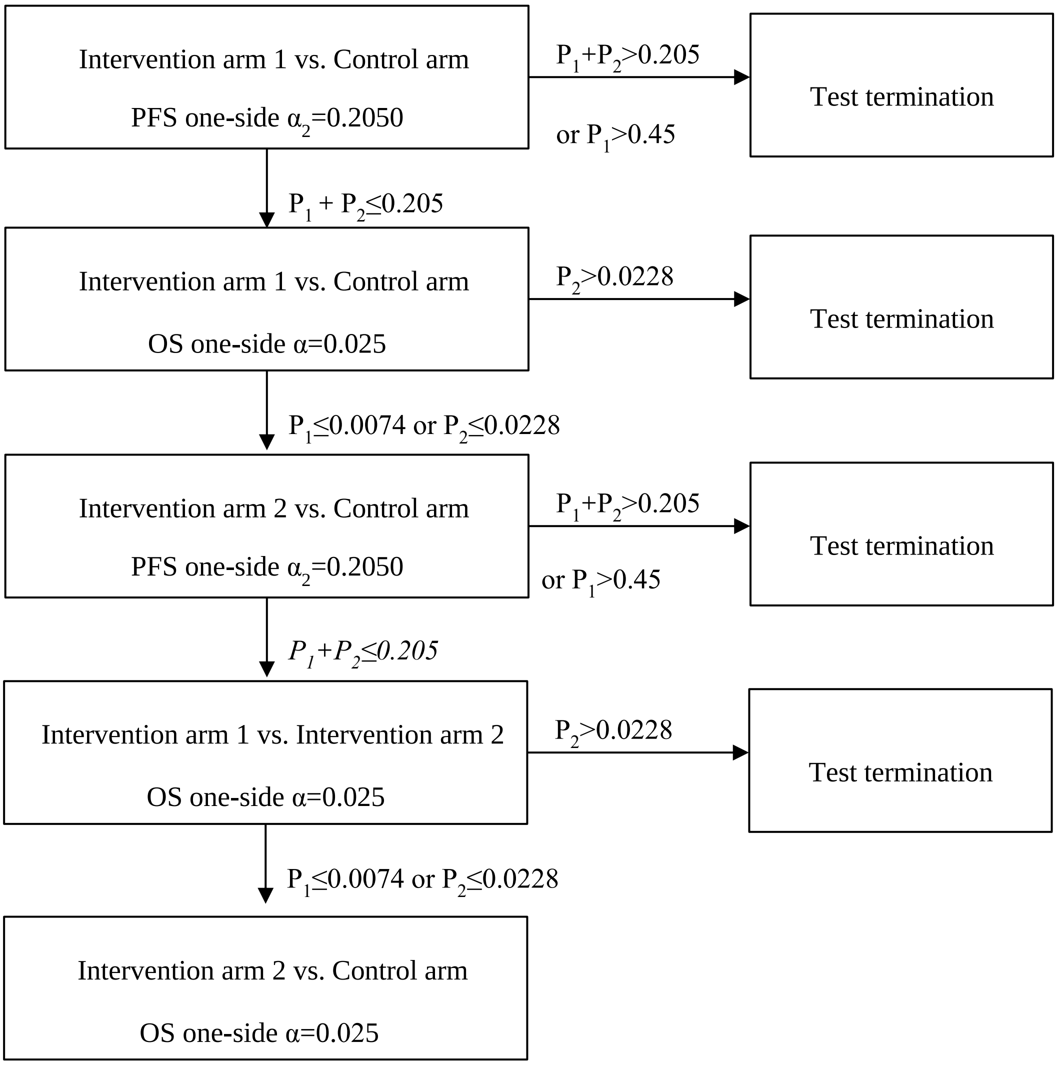

A two-sided test with a significance level of α=0.05 will be used for secondary efficacy endpoints and safety analysis. Two-sided $P \le 0.05$ will be considered statistically significant with a 95%CI.

13.2.3 Hypothesis

The primary efficacy endpoints of this trial are PFS and OS.

Comparison between groups will be performed using a stratified log-rank test:

- PFS hypothesis 1 **(**Intervention arm 1 vs. Control arm**)**

$H0_{(PFS1)}$: PFS in intervention arm 1=PFS in the control arm, that is, $S_1(t)=S_2(t)$;

$H1_{(PFS1)}$: PFS in intervention arm 1≠PFS in the control arm, that is, $S_1(t) \neq S_2(t)$;

If the test for PFS does not reject the null hypothesis $H0_{(PFS1)}$, all subsequent tests will be terminated; if $H0_{(PFS2)}$ is rejected, tests will be continued. This result will demonstrate the superiority of PFS in intervention arm 1 compared with the control arm.

- OS hypothesis 1 (Intervention arm 1 vs. Control arm)

$H0_{(OS1)}$: OS in intervention arm 1=OS in the control arm, that is, $S_1(t)=S_2(t)$;

$H1_{(OS1)}$: OS in intervention arm 1≠OS in the control arm, that is, $S_1(t)≠S_2(t)$;

If the test for OS does not reject the null hypothesis $H0_{(OS1)}$, all subsequent tests will be terminated; if $H0_{(OS1)}$ is rejected, tests will be continued.

- PFS hypothesis 2 (Intervention arm 2 vs. Control arm)

$H0_{(PFS2)}$: PFS in intervention arm 2=PFS in the control arm, that is, $S_1(t)=S_2(t)$;

$H1_{(PFS2)}$: PFS in intervention arm 2≠PFS in the control arm, that is, $S_1(t)≠S_2(t)$;

If the test for PFS does not reject the null hypothesis $H0_{(PFS2)}$, all subsequent tests will be terminated; if $H0_{(PFS2)}$ is rejected, tests will be continued.

- OS hypothesis 3 (Intervention arm 1 vs. Intervention arm 2)

$H0_{(OS3)}$: OS in intervention arm 1=OS in the control arm, that is, $S_1(t)=S_2(t)$;

$H1_{(OS3)}$: OS in intervention arm 1≠OS in the control arm, that is, $S_1(t)≠S_2(t)$;

If the test for OS does not reject the null hypothesis $H0_{(OS3)}$, all subsequent tests will be terminated; if $H0_{(OS3)}$ is rejected, tests will be continued.

- OS hypothesis 2 (Intervention arm 2 vs. Control arm)

$H0_{(OS2)}$: OS in intervention arm 2=OS in the control arm, that is, $S_1(t)=S_2(t)$;

$H1_{(OS2)}$: OS in intervention arm 2≠OS in the control arm, that is, $S_1(t)≠S_2(t)$;

13.3 Study Population

13.3.1 Subject Disposition

Subject disposition (analysis of population allocation, enrollment at each center, loss to follow-up, treatment period, and treatment discontinuation) will be summarized using frequency and percentage.

Subject allocation and reasons for analysis exclusion in each analysis set will be summarized.

Listings will be provided for subjects who are excluded for analysis (specific protocol deviations and reasons for exclusion).

A by-subject listing will be provided to describe the treatment and reasons for treatment discontinuation.

13.3.2 Protocol Deviation

A by-subject listing of subjects with protocol deviations will be provided by the intervention arm in the ITT population.

13.3.3 Demographics and Baseline Characteristics

Age will be summarized by mean, SD, minimum, maximum, and median.

Gender will be summarized by frequency counts.

Height and weight will be summarized by mean, SD, minimum, maximum, and median.

Tumor history, including TNM stage, history of antitumor therapy, and smoking history, will be summarized by frequency counts.

Baseline vital signs (temperature, heart rate, breathing, and blood pressure): baseline is defined as the latest non-missing assessment before the first dose.

Baseline ECOG PS will be summarized by mean, SD, minimum, maximum, and median.

13.4 Efficacy Analysis

13.4.1 Analysis of Primary Efficacy Endpoints

- **PFS**

The median PFS will be estimated using the Kaplan-Meier method and presented with the Kaplan-Meier curve.

The comparison of PFS between intervention arms will be performed using a stratified log-rank test. The HR and the corresponding 95%CI will also be provided based on a Cox proportional hazard model, using ECOG PS (0 vs. 1), brain metastases (yes vs. no), and liver metastases (yes vs. no) as concomitant variables. The results of the unstratified log-rank test and results of the Cox proportional hazard model without covariates adjustment will also be provided.

Subgroup analysis of PFS will be performed based on stratification factors.

- **OS**

The median OS will be estimated using the Kaplan-Meier method and presented with the Kaplan-Meier curve.

The comparison of OS between intervention arms will be performed using a stratified log-rank test. The HR and the corresponding 95%CI will also be provided based on a Cox proportional hazard model, using ECOG PS (0 vs. 1), brain metastases (yes vs. no), liver metastases (yes vs. no), and treatment after patient withdrawal (yes vs. no) as concomitant variables. The results of the unstratified log-rank test and results of the Cox proportional hazard model without covariates adjustment will also be provided.

Subgroup analysis of OS will be performed based on stratification factors.

13.4.2 Analysis of Secondary Efficacy Endpoints

- **ORR**

The number and percentage of subjects with an objective response (PR or CR) will be presented. The ORR and corresponding 95%CI will be summarized using F-distribution by binomial probabilities. The difference in ORR between intervention arms will be compared by the stratified Cochran-Mantel-Haenszel (CMH) test. Stratification factors as items specified in section 4.3.

- **DOR**

The median DOR will be estimated using the Kaplan-Meier method and presented with the Kaplan-

Meier curve. The comparison of DOR between intervention arms will be performed using a stratified log-rank test. DOR will be only analyzed in responders.

- **DCR**

The number and percentage of subjects with the disease control (PR, CR, or SD) will be presented. The DCR and corresponding 95%CI will be summarized using F-distribution by binomial probabilities. The difference in DCR between intervention arms will be compared by the stratified CMH test. Stratification factors as items specified in section 4.3.

- **6-month and 12-month PFS rates**

The PFS rate and the corresponding 95%CI for each intervention arm will be estimated using the Kaplan-Meier method and compared with Fisher's Exact Test.

- **12-month and 18-month OS rates**

The OS rate and the corresponding 95%CI for each intervention arm will be estimated using the Kaplan-Meier method and compared with Fisher's Exact Test.

- **HRQOL**

HRQOL will be summarized by descriptive statistics. The changes of each item from baseline at each scheduled visit will be summarized by mean, SD, median, minimum, and maximum within each intervention arm. The grade of each item will be presented by the number and percentage and compared with the Wilcoxon rank sum test between intervention arms.

13.5 Safety Analysis

13.5.1 Treatment Exposure and Compliance

Treatment exposure will be summarized by mean, SD, minimum, maximum, and median in each intervention arm.

Treatment exposure, the completed treatment cycle, dose adjustment and reasons, and the cumulative number of dose adjustments will be summarized.

Treatment duration, cumulative dose, dose intensity, and dosing compliance during the treatment will be statistically described.

The dosing compliance will be calculated based on the sum of all actual doses (defined as the values entered on the actual dose administered field on the eCRF) taken across the treatment period and the sum of protocol-defined doses.

13.5.2 AEs

All AEs will be coded using the Medical Dictionary for Regulatory Affairs (MedDRA) for safety analysis.

An overview table of AEs will be provided for each intervention arm by the MedDRA preferred term (PT) and primary system organ class (SOC), including the number of events and the number (incidence) of subjects with the following: all AEs, AEs before the first dose, treatment-emergent AEs (TEAEs), significant TEAEs, TEAEs of special interest, TEAEs with grade 3 or more, serious

TEAEs (SAEs), treatment-related TEAEs, treatment-related SAEs, TEAEs leading to dose adjustment, treatment discontinuation, study termination, and death.

TEAEs or treatment-related TEAEs with ≥5% incidence will also be summarized by PT.

Treatment-related TEAEs with grade 3 or above (as per NCI-CTCAE criteria) will also be summarized by PT.

TEAEs or treatment-related TEAEs with ≥ 10% incidence will also be summarized by PT.

A frequency table of AEs will be provided for each intervention arm by SOC and PT. The incidence will be calculated by the system, symptoms, and vital signs (the number of events is defined as the number of patients with at least one event).

The number (percentage) of patients with TEAEs will also be provided by severity grade per NCI-CTCAE v5.0 and the causality to the study drugs. A patient will be counted only once by the worst severity grade per NCI-CTCAE v5.0.

Listings of TEAEs and SAEs will be provided.

The incidence of TEAEs and treatment-related TEAEs between each arm will be compared with the Fisher's Exact Test, when necessary.

TEAEs of special interest and the median time of the first occurrence.

13.5.3 Vital Signs

The values and changes in vital signs before and after the treatment will be summarized by mean, SD, minimum, maximum, and median in each intervention arm.

13.5.4 Laboratory Test Abnormalities

Laboratory values will be descriptively summarized by the intervention arm. Quantitative variables (eg., hemanalysis and biochemistry) will be summarized by mean, SD, minimum, maximum, and median, as well as their changes from baseline within each intervention arm.

Laboratory values will be classified into lower or higher than normal according to the range of normal values. The abnormal changes from baseline will be presented with the crosstab. The clinically meaningful abnormalities will be judged by investigators and summarized by the number and percentage.

13.5.5 ECG

ECG will be identified to be normal or abnormal by investigators' judgment. The changes from baseline in ECG will be summarized.

The values and changes from the baseline of heart rate, PR interval, QRS interval, QT interval, and QTc will be summarized by mean, SD, minimum, maximum, and median. The overall results of ECG will be presented with the crosstab. The clinically meaningful abnormalities will be judged by investigators and summarized by the number and percentage.

Listings of ECG results will be provided in each arm.

13.5.6 Concomitant Medications

The number and percentage of concomitant medications during screening, treatment, and follow-up periods will be summarized. The Anatomical Therapeutic Chemical (ATC) codes in WHODrug Global will be used for coding concomitant medications. The frequency of concomitant medications will be listed by main anatomical groups and therapeutic subgroups in the SS population.

13.6 Safety Analysis Software

The data management will be provided by Chia Tai Tianqing Pharmaceutical Group Co., Ltd. The statistical analysis will be conducted by the Department of Biostatistics, School of Public Health, Nanjing Medical University. The statistical analysis will be performed using the SAS statistical package, version 9.4.

14. Data Keeping

To ensure the evaluation and supervision by the State Food and Drug Administration and sponsor, all documents must be archived by the research center and treated as confidential material, including confirmed documents (those that can effectively check different records, such as medical records), signed informed consent forms, and detailed source records of drug allocation. Data will be stored under lock and key for 5 years. The ownership of all documents will belong to Chia Tai Tianqing Pharmaceutical Group Co., Ltd. Investigator(s) shall not provide it to any third party in any form without the written consent of the sponsor, except for State Food and Drug Administration.

15. Sponsor/Investigator Responsibilities

15.1 Sponsor

(1) Provide support for the investigator(s) and be responsible for familiarising the investigator(s) involved in the trial with all study procedures before study initiation;

(2) Provide clinical research associates (CRA) for clinical monitoring and regular visits;

(3) CRA should ensure that they can keep in touch with the investigator(s) at any time by telephone, fax, and mail;

(4) CRA must supervise investigator(s) to conduct the investigation following approved protocol, ensure the standard allocation and recovery of investigational drugs according to specifications, and conduct the source data verification and review to ensure data consistency.

15.2 Investigator

(1) Investigator(s) must receive training for GCP and this protocol and have time to conduct this trial according to the protocol;

(2) Investigator(s) must inform detailed information for subjects and obtain informed consent before enrollment;

(3) Investigator(s) have the obligation to take necessary measures to ensure the safety of patients. Any AEs should be reported to the principal investigator immediately and followed up with the SAEs;

(4) Investigator(s) must complete medical records carefully and timely;

(5) Investigator(s) must actively cooperate with CRA for regular visits;

(6) Investigator(s) must ensure the completeness of laboratory records, clinical records, and original medical records.

16. Ethical Considerations and Informed Consent

This trial will be conducted in accordance with the ethical principle of the Declaration of Helsinki (version 2010) and guidelines for clinical trials in China. The study protocol will be determined and signed by the sponsor and investigator and submitted for approval to the ethical committee of Jilin Tumor Hospital before any study-related procedures. Any significant modifications to the study protocol will be submitted for approval to the local medical ethical committee, and meanwhile require a formal amendment to the protocol. Any updated information on investigational drugs will be amended in informed consent and submitted for approval to the local medical ethical committee. The approved informed consent must be obtained again.

Investigator(s) at each center will ensure that the subject is given full and adequate oral and written information about the nature, purpose, possible risks and benefits of the trial, any alternative treatments, and the rights and obligations of subjects. After the trial has been fully explained, written informed consent will be obtained from either the subject or their legal representative prior to study participation.

17. Study Summary

A reasonable statistical analysis, a detailed summary, and the completed chart will be provided according to the results. All documents must be signed by the investigator(s) and inspectors.

18. References

(1) Guiding Principles for Pharmacokinetic Study of Chemical Drugs, SFDA, 2005.

(2) Guiding Principles for Clinical Trial for Antitumor Drugs, SFDA, 2007.03.

(3) Chinese Society of Clinical Oncology (CSCO) Guidelines for Diagnosis and Treatment of Primary Lung Cancer 2019.

(4) Puzanov, A, et al. Managing Toxicities Associated with Immune Checkpoint Inhibitors: Consensus Recommendations from the Society for Immunotherapy of Cancer (SITC) Toxicity Management Working Group. Journal for immuno therapy of cancer (2017) 5: 95.

(5) L.Horn, et al. First-line Atezolizumab plus Chemotherapy in Extensive-stage Small-Cell Lung Cancer. The New England Journal of Medicine, Sep 25, 2018.

(6) Phase III Trial Comparing Supportive Care Alone with Supportive Care with Oral Topotecan in Patients with Relapsed Small-Cell Lung Cancer, 2006, J Clin Oncol 24: 5441-5447.

(7) Randomized Phase III Trial of Amrubicin Versus Topotecan as Second-Line Treatment for Patients with Small-Cell Lung Cancer. Journal of Clinical Oncology, 2014, 32 (35): 4012-4020.

(8) Randomized Phase III trial of Amrubicin/Cisplatin Versus Etoposide/Cisplatin as First-Line

Treatment for Extensive Small-Cell Lung Cancer. BMC Cancer (2016) 16:265.

(9) Randomized Phase II Trial of Single-Agent Amrubicin or Topotecan as Second-Line Treatment in Patients with Small-Cell Lung Cancer Sensitive to First-Line Platinum-Based Chemotherapy. Journal of Clinical Oncology, 2011, 29 (3): 287-293.

(10) Randomized Phase II Trial Comparing Amrubicin with Topotecan in Patients with Previously Treated Small-Cell Lung Cancer: North Japan Lung Cancer Study Group Trial 0402. Journal of Clinical Oncology, 2008, 26 (33): 5401-5406.

(11) Prophylactic Cranial Irradiation for Patients with Small-Cell Cancer in Complete Remission. Prophylactic Cranial Irradiation Overview Collaborative Group. N Engl J Med,1999, 341 (7): 476-484.

(12) Prophylactic Cranial Irradiation in Extensive Small-Cell Lung Cancer. N Engl J Med, 2007, 357 (7): 664-672.

(13) Prophylactic Cranial Irradiation in Small-Cell Lung Cancer: A Systematic Review of Literature with Meta-Analysis. BMC Cancer, 2001, 1:5. Epub 2001 Jun 19.

(14) Standard-Dose Versus Higher-Dose Prophylactic Cranial Irradiation (PCI) in Patients with Limited-Stage Small-Cell Lung Cancer in Complete Remission after Chemotherapy and Thoracic Radiotherapy (PCI 99-01, EORTC 22003-08004, RTOG 0212, and IFCT 99-01): A Randomised Clinical Trial. Lancet Oncol, 2009, 10 (5): 467-474.

(15) Prophylactic Cranial Irradiation in Extensive Disease Small-Cell Lung Cancer: Short-Term Health-Related Quality of Life and Patient Reported Symptoms: Results of An International Phase III Randomized Controlled Trial by EORTC Radiation Oncology and Lung Cancer Groups. J Clin Oncol, 2009, 27 (1): 78-84.

(16) NCCN Guideline Version 1.2016 Small Cell Lung Cancer.

(17) First-Line Atezolizumab plus Chemotherapy in Extensive-Stage Small-Cell Lung Cancer. N ENGL J MED, 2018.
