# Supplementary material for: Benmelstobart, anlotinib and chemotherapy in extensive-stage small-cell lung cancer: a randomized phase 3 trial
Source: Nat Med. 2024 Jul 11;30(10):2967–76. doi: 10.1038/s41591-024-03132-1 (PMC11485241; doi:10.1038/s41591-024-03132-1)
Supplement: Supplementary file 2 — Reporting Summary [file 41591_2024_3132_MOESM2_ESM.pdf]

nature portfolio

Corresponding author(s): Ying Cheng

Last updated by author(s): Jun 29, 2024

Reporting Summary

Nature Portfolio wishes to improve the reproducibility of the work that we publish. This form provides structure for consistency and transparency in reporting. For further information on Nature Portfolio policies, see our Editorial Policies and the Editorial Policy Checklist.

Statistics

For all statistical analyses, confirm that the following items are present in the figure legend, table legend, main text, or Methods section.

n/a	Confirmed	
☐	☒	The exact sample size (*n*) for each experimental group/condition, given as a discrete number and unit of measurement
☐	☒	A statement on whether measurements were taken from distinct samples or whether the same sample was measured repeatedly
☐	☒	The statistical test(s) used AND whether they are one- or two-sided<br>*Only common tests should be described solely by name; describe more complex techniques in the Methods section.*
☐	☒	A description of all covariates tested
☐	☒	A description of any assumptions or corrections, such as tests of normality and adjustment for multiple comparisons
☐	☒	A full description of the statistical parameters including central tendency (e.g. means) or other basic estimates (e.g. regression coefficient) AND variation (e.g. standard deviation) or associated estimates of uncertainty (e.g. confidence intervals)
☐	☒	For null hypothesis testing, the test statistic (e.g. *F*, *t*, *r*) with confidence intervals, effect sizes, degrees of freedom and *P* value noted<br>*Give P values as exact values whenever suitable.*
☒	☐	For Bayesian analysis, information on the choice of priors and Markov chain Monte Carlo settings
☐	☒	For hierarchical and complex designs, identification of the appropriate level for tests and full reporting of outcomes
☒	☐	Estimates of effect sizes (e.g. Cohen's *d*, Pearson's *r*), indicating how they were calculated

Our web collection on statistics for biologists contains articles on many of the points above.

Software and code

Policy information about availability of computer code

Data collection	Investigator(s) or designated representative record all data for each patient through eCRFs using the Electronic Data Capture (EDC) system.
Data analysis	The statistical analysis will be performed using the SAS statistical package, version 9.4.

For manuscripts utilizing custom algorithms or software that are central to the research but not yet described in published literature, software must be made available to editors and reviewers. We strongly encourage code deposition in a community repository (e.g. GitHub). See the Nature Portfolio guidelines for submitting code & software for further information.

Data

Policy information about availability of data

All manuscripts must include a data availability statement. This statement should provide the following information, where applicable:

- Accession codes, unique identifiers, or web links for publicly available datasets
- A description of any restrictions on data availability
- For clinical datasets or third party data, please ensure that the statement adheres to our policy

All data required to interpret, verify, or build new research on the published claims are included in the article or uploaded in the Supplementary Information. We cannot share individual de-identified participant data due to the risk of re-identification and loss of patient confidentiality.

Research involving human participants, their data, or biological material

Policy information about studies with human participants or human data. See also policy information about sex, gender (identity/presentation), and sexual orientation and race, ethnicity and racism.

Reporting on sex and gender	Both male and female patients were eligible for enrollment. Sex was self-reported and recorded by the practicing clinician. As lung cancer mainly affected male patients, which was consistent with out trial enrollment (85% male in Benmelstobart +Anlotinib group, 83.3% male in Anlotinib group; 83.8% male in Chemotherapy group). The protocol explicitly includes "sex" as one of the factors for survival subgroup analysis (Fig. 2b and Fig. 3b).
Reporting on race, ethnicity, or other socially relevant groupings	There were no analyses based on race or ethnicity in this manuscript.
Population characteristics	Baseline demographic and disease characteristics were reported in Table 1.
Recruitment	From March 18, 2020, through December 18, 2021, patients were recruited by investigators at 72 hospitals across China. Eligible participants were enrolled without selection. Eligibility criteria and screening procedures minimized the potential of selection bias.. Potential patients were identified by the investigators or staff at the participating centers based their diagnosis. The investigator at each center ensured that all patients were given full and adequate oral and written information about the nature, purpose, possible risk, and benefit of the study. Patients were also notified that they were free to discontinue from the study at any time. Patients were recruited according to the stud protocol and provided written informed consent before recruitment.
Ethics oversight	The trial was conducted per the principles of Good Clinical Practice guidelines and the Declaration of Helsinki. Approvals for the trial protocol (and any protocol modifications) were obtained from independent ethics committees of each participating center (listed in supplementary information) and central approval was obtained from the Jinlin Province Cancer Hospital Institutional Review Board (Ethics Approval No. 201909-053-01). None of the study participants received compensation for participation in the study.

Note that full information on the approval of the study protocol must also be provided in the manuscript.

Field-specific reporting

Please select the one below that is the best fit for your research. If you are not sure, read the appropriate sections before making your selection.

☒ Life sciences ☐ Behavioural & social sciences ☐ Ecological, evolutionary & environmental sciences

For a reference copy of the document with all sections, see nature.com/documents/nr-reporting-summary-flat.pdf

Life sciences study design

All studies must disclose on these points even when the disclosure is negative.

Sample size	The statistical plan is provided in Supplementary protocol. A fixed-sequence test was used for multiple testing between treatment groups. Testing begins with the first hypothesis, H1, and each test is carried out without a multiplicity adjustment, provided that significant results are observed in all preceding tests. The fixed-sequence procedure controls the family-wise error rate because, for each hypothesis, testing is conditional upon rejecting all hypotheses earlier in the sequence. The median PFS of 4 months and median OS of 10 months were expected in the EC alone group based on existing data. We assumed a 12-month recruitment period, 18 months of follow-up, 10% dropout rate, a power of 85% at a two-sided significance level of 0.05.<br>Sample size estimation in the benmelstobart and anlotinib plus EC group: Assuming that the HR for PFS in the benmelstobart and anlotinib plus EC group compared with the EC alone group is 0.6, the hazard rate in the benmelstobart and anlotinib plus EC group is 0.1040, and that in the EC alone group is 0.1733 along with exponential distribution. Approximately 165 PFS events are expected if 174 patients are enrolled. A total of 194 patients are recruited and randomly assigned (1:1) to benmelstobart and anlotinib plus EC group and control arm, considering an approximate dropout incidence of 10%. The interim analysis will be conducted when the planned PFS events are ~83. The final analysis will be performed after about 165 of the planned PFS events. Assuming that the HR for OS in benmelstobart and anlotinib plus EC group compared with the EC alone group is 0.7, 284 OS events are expected with the type I error rate of 0.050 if 381 patients are enrolled. A total of 424 patients were recruited and randomly assigned (1:1) to benmelstobart and anlotinib plus EC group and control arm, considering an approximate dropout incidence of 10%.<br>Sample size estimation in the anlotinib plus EC group: Assuming that the HR for PFS in the anlotinib plus EC group compared with the EC alone group is 0.65, the hazard rate in the anlotinib plus EC group is 0.1126, and that in the EC alone group is 0.1733 along with exponential distribution. Approximately 231 PFS events are expected if 242 patients are enrolled. A total of 270 patients are recruited and randomly assigned (1:1) to the anlotinib plus EC group and control arm, considering an approximate dropout incidence of 10%. The interim analysis will be conducted when the planned PFS events are ~116. The final analysis will be performed after about 231 of the planned PFS events. Assuming that the HR for OS in the anlotinib plus EC group compared with the EC alone group is 0.72, 331 OS events are expected with the type I error rate of 0.050 if 442 patients are enrolled. A total of 492 patients were recruited and randomly assigned (1:1) to the anlotinib plus EC group and control arm, considering an approximate dropout incidence of 10%.<br>Combined the sample size estimation for PFS and OS, 738 subjects will be finally enrolled in this trial.
Data exclusions	Data were incorporated in accordance with protocol requirements; no data were excluded from the efficacy and safety analysis.

Replication	This is a clinical trial. No replication was done.
Randomization	The study was conducted in a double-blind, double-dummy design. All eligible patients were centrally randomly assigned in a 1:1:1 ratio to receive one of the three regimens: benmelstobart, anlotinib, and etoposide/carboplatin followed by benmelstobart and anlotinib maintenance (benmelstobart plus anlotinib group); benmelstobart placebo, anlotinib, and etoposide/carboplatin followed by anlotinib maintenance (anlotinib group); or placebos for benmelstobart and anlotinib, plus etoposide/carboplatin followed by placebo maintenance (etoposide/carboplatin group). Randomization was conducted by the central stratified randomization method, and stratified by ECOG PS (0 vs. 1), brain metastases (yes vs. no), and liver metastases (yes vs. no). Randomization list was generated by an independent statistician using an SAS statistical package (version 9.4, SAS Institute, Cary, NC, USA) and secured in the eRand central randomization system with restricted access to only designated unblinded independent statistician. Subjects enrolled in this trial and meeting the inclusion/exclusion criteria will be sequentially assigned to individual groups through the eRand central randomization system according to their randomization numbers.
Blinding	Patients and their families or guardians, investigators, local and central radiological reviewers, the study statistician, data management personnel who will be involved in data cleaning and analysis of the data, and the study sponsor were masked to treatment allocation until the final database was locked.

Reporting for specific materials, systems and methods

We require information from authors about some types of materials, experimental systems and methods used in many studies. Here, indicate whether each material, system or method listed is relevant to your study. If you are not sure if a list item applies to your research, read the appropriate section before selecting a response.

Materials & experimental systems

n/a	Involved in the study
☒	☐ Antibodies
☒	☐ Eukaryotic cell lines
☒	☐ Palaeontology and archaeology
☒	☐ Animals and other organisms
☐	☒ Clinical data
☒	☐ Dual use research of concern
☒	☐ Plants

Methods

n/a	Involved in the study
☒	☐ ChIP-seq
☒	☐ Flow cytometry
☒	☐ MRI-based neuroimaging

Clinical data

Policy information about clinical studies

All manuscripts should comply with the ICMJE guidelines for publication of clinical research and a completed CONSORT checklist must be included with all submissions.

Clinical trial registration	ClinicalTrials.gov: NCT04234607.
Study protocol	Study protocol was included in the submission.
Data collection	From March 18, 2020, to December 18, 2021, 1005 patients at 72 sites were assessed for eligibility. 738 were enrolled in the ETER701 trial and randomized to treatment. The data cut-off date was May 14, 2022. All clinical sites are listed in the supplementary information. The data collection were conducted using the electronic data capture system. The clinical data were collected in Electronic Case Report Forms. Tumor assessments per RECIST 1.1 and iRECIST were performed using computed tomography or magnetic resonance imaging at screening and every 2 cycles from randomization until imaging-based disease progression, the start of new antitumor treatments, consent withdrawal, or death.). Adverse events were monitored and graded according to the National Cancer Institute Common Terminology Criteria for Adverse Events (version 5.0).
Outcomes	The ETER701 trial assessed two primary efficacy end points: progression-free survival (PFS) assessed by an independent review committee (IRC) per RECIST 1.1 (defined as the time from randomization to the first documented progressive disease or death from any cause) and OS (defined as the time from randomization to death from any cause). Secondary end points were PFS assessed by the investigator per RECIST 1.1 and iRECIST; the IRC-assessed objective response rate (ORR) as per RECIST 1.1 (defined as the percentage of participants achieving complete response and partial response); disease control rate (DCR, defined as the percentage of participants achieving a complete response, partial response, and stable disease); duration of response (DoR, defined as the time from first documented evidence of complete response or partial response until progressive disease or death, whichever occurred first); 6-month and 12-month PFS probability; 12-month and 18-month OS probability; the health-related quality of life (HRQoL) evaluated with the EQ-5D visual analogue scale (EQ-VAS) questionnaire; and safety.

Plants

Seed stocks	Not applicable.
Novel plant genotypes	Not applicable.
Authentication	Not applicable.
